# Costs and benefits of early access to new cancer drugs through the US Food and Drug Administration’s accelerated approval pathway: retrospective observational study and economic evaluation

**DOI:** 10.1136/bmjmed-2025-001934

**Published:** 2025-12-16

**Authors:** Huseyin Naci, Mahnum Shahzad, Peter Murphy, Yichen Zhang, Rebecca Costa, Joseph S Ross, Anita K Wagner

**Affiliations:** 1 Pharmaceutical Policy Lab, London, UK; 2 Department of Health Policy, London School of Economics and Political Science, London, UK; 3 Division of Health Policy and Insurance Research, Department of Population Medicine, Harvard Pilgrim Health Care Institute, Boston, MA, USA; 4 Harvard Medical School, Boston, MA, USA; 5 Centre for Health Economics, University of York, York, UK; 6 Department of Pharmacy Administration and Clinical Pharmacy, Peking University, Beijing, China; 7 Internal Medicine, Yale School of Medicine, New Haven, CT, USA; 8 Yale Collaboration for Regulatory Rigor Integrity and Transparency, New Haven, CT, USA; 9 Department of Health Policy and Management, Yale School of Public Health, New Haven, CT, USA; 10 Yale School of Medicine Center for Outcomes Research & Evaluation, New Haven, CT, USA

**Keywords:** Health policy, Medical oncology

## Abstract

**Objective:**

To evaluate the survival gains and additional Medicare spending associated with early access to new cancer drugs granted accelerated approval by the US Food and Drug Administration (FDA), compared with access to cancer drugs following completion of confirmatory trials.

**Design:**

Retrospective observational study and economic evaluation.

**Setting:**

US Medicare programme, which provides health insurance for adults aged 65 years and older.

**Participants:**

Medicare beneficiaries who received new cancer drug indications from the time of FDA accelerated approval to conversion to regular approval, withdrawal from the market, or 31 December 2020.

**Interventions:**

Use of cancer drugs for indications that initially received FDA accelerated approval between 1 January 2012 and 31 December 2020.

**Main outcomes and measures:**

Primary outcomes were life year gains and additional Medicare spending associated with cancer drug indications that initially received FDA accelerated approval. Each indications’s overall survival benefit to patients, if present, was determined, and a partitioned survival model was developed to estimate the life year gains and incremental Medicare spending attributable to early drug access. Beneficiaries who received accelerated approval indications were followed for survival and spending outcomes until 31 December 2022.

**Results:**

An estimated 178 708 Medicare beneficiaries received access to 90 new cancer drug indications from the time of accelerated approval to conversion to regular approval, withdrawal from the market, or 31 December 2020. Seventeen per cent of beneficiaries (n=30 374) received drugs for indications that were ultimately withdrawn, 5574 (3.1%) received drugs for indications that remained under accelerated approval, and 142 760 (79.9%) received drugs for indications that were subsequently converted to regular approval. Overall, 80 885 (45.3%) beneficiaries received drugs for indications that provided an overall survival benefit to patients. Between 2012 and 2022, use of these drug indications was associated with an estimated 76 164 life years gained. Only three accelerated approvals (nivolumab to treat melanoma, pembrolizumab to treat non-small cell lung cancer, and pemetrexed to treat non-small cell lung cancer) accounted for 68.4% (n=52 107 years) of the total life year gains. The estimated additional cost of early access to these drugs to Medicare was $20.1bn (£15.4bn; €17.4bn) corresponding to a mean spending of $263 371 (95% confidence interval $180 139 to $373 884) per life year gained. Additional mean Medicare spending per life year gained ranged from $25 947 ($18 174 to $39 829) for melanoma indications to $4.5m ($3.1m to $12.2m) for breast cancer indications.

**Conclusions:**

This study examined the trade-off between the benefits of earlier access to cancer drugs through the FDA's accelerated approval pathway and the uncertainty surrounding their clinical efficacy at the time of market entry. Accelerated approval yielded uneven survival returns, with a small number of drugs accounting for the majority of life year gains. Medicare incurred substantial costs for treatments that, in over half of cases, did not provide an overall survival benefit to patients.

**WHAT IS ALREADY KNOWN ON THIS TOPIC**

- Cancer drugs account for the majority of US Food and Drug Administration (FDA) accelerated approvals, a pathway that facilitates earlier access to new drugs based on trials using surrogate markers as efficacy endpoints
- The implementation of the accelerated approval pathway has been controversial for cancer drugs, because most drug indications have not demonstrated overall survival benefits in confirmatory trials

**WHAT THIS STUDY ADDS**

- This analysis examines the trade-off between the benefit of early access to cancer drug indications against the costs to Medicare associated with greater uncertainty around the drug’s efficacy
- Most Medicare beneficiaries received treatments that provided minimal or no overall survival gains
- The additional Medicare spending per life year gained was substantial and varied considerably across cancer types

**HOW THIS STUDY MIGHT AFFECT RESEARCH, PRACTICE, OR POLICY**

- Timely completion of confirmatory trials measuring overall patient survival, followed by appropriate regulatory action, would help direct Medicare funds toward treatments with proven clinical benefits
- The FDA should clearly communicate the degree of uncertainty about drugs granted accelerated approval to support informed decision making by clinicians and patients

##  Introduction

The US Food and Drug Administration (FDA) faces a challenge in balancing timely access to new therapeutics with the need for robust evidence demonstrating their safety and efficacy. The accelerated approval pathway, introduced by the FDA in 1992, was designed to expedite access to drugs for patients with serious conditions and unmet medical needs.^1^ This pathway allows drug approval based on surrogate endpoints—interim measures such as laboratory tests or imaging results—that are reasonably likely to predict clinical benefit.^2^ While surrogate endpoints enable faster clinical trial testing and drug development,^3^ they introduce substantial uncertainty regarding the clinical benefits of approved drugs, as positive outcomes from trials using surrogate endpoints may not reliably correlate with how patients feel, function, or survive.^4 5^ Characteristics of clinical studies supporting accelerated approvals also contribute to this uncertainty; they often lack control groups, have small patient populations, and rarely use features like blinding.^6^,^8^

To reduce this uncertainty, the FDA requires post-marketing confirmatory trials to verify the clinical benefit of drugs granted accelerated approval. However, the implementation of this requirement has been inconsistent:^9^,^12^ between 1992 and 2020, nearly half of the drug indications that have received accelerated approval did not have completed confirmatory trials.^13^ Trial completion is often delayed,^14^ and some drugs without verified benefit remain on the market for extended periods. Even when confirmatory trials are completed, concerns persist about the quality of evidence used to determine clinical benefit. In more than half of accelerated approval, surrogate endpoints were used not only to grant accelerated approval but also to establish benefit in confirmatory trials.^15^ Enforcing regulatory action after the completion of confirmatory trials has also been delayed, including in cases where trials failed to demonstrate clinical benefit.^16^

The implementation of the accelerated approval pathway has been particularly controversial for cancer drugs, which accounted for more than 80% of accelerated approvals between 2010 and 2020. A weak to moderate association exists between surrogate endpoints used in cancer drug trials, including patient response rate and progression-free survival, and patient survival or quality of life.^17^,^20^ Thus, drugs that have positive results in trials using surrogate endpoints often do not confer survival gains or improvements in how patients feel or function.^21^ Between 1992 and 2017, only 20% of cancer drug indications judged to have verified clinical benefit by the FDA provided an overall survival benefit to patients.^22^ Moreover, 22% of cancer drug indications with accelerated approvals between 2013 and 2023 were subsequently withdrawn from the market because of a lack evidence for their clinical benefit.^23^

The accelerated approval pathway must balance the benefits of early patient access to drugs against the risks of approving drugs with uncertain evidence for their efficacy.^24^ The FDA states that this pathway has enabled cancer drugs to enter the market sooner than they otherwise would.^3 25 26^ Earlier access has benefitted some patients for whom the survival gains associated with treatment were substantial, such as those treated with imatinib, which improved outcomes for patients with chronic myeloid leukaemia.^27^ However, not all patients benefit from the accelerated approval pathway: some receive drugs later shown to lack clinical benefit, and in some cases, drugs that may cause harm.^28^ Early access to expensive new cancer treatment also incurs substantial healthcare costs for patients and the healthcare system.^29^

Earlier research has not evaluated the cost-benefit balance of the accelerated approval pathway. It is not known whether the FDA strikes an optimal balance between expedited approval and demonstration of efficacy for new cancer drugs that initially receive accelerated approval. The cost-benefit balance of the accelerated approval pathway is particularly important for the federal Medicare programme, which provides health insurance for Americans aged 65 years and older. Medicare consists of two main components: traditional Medicare, administered by the government, and Medicare Advantage, offered by private insurers.^30^ More than 50% of US cancer diagnoses are received by Medicare beneficiaries, with substantial cost implications to the programme.^31^ Between 2016 and 2020, the share of Medicare drug spending on cancer drugs increased from 33.7% to 43.1% in part B, which covers injectable drugs, and from 9.1% to 13.2% in part D, which covers outpatient drugs.^32^ Spending for drugs with accelerated approvals comprises a growing portion of this increase in Medicare’s overall cancer drug spending.^33^

In this study, we evaluated the expected patient survival benefits and additional Medicare spending associated with early access to new cancer drug indications granted accelerated approval by the FDA, compared with access following regular approval.

## Methods

### Overview

**Figure 1:**
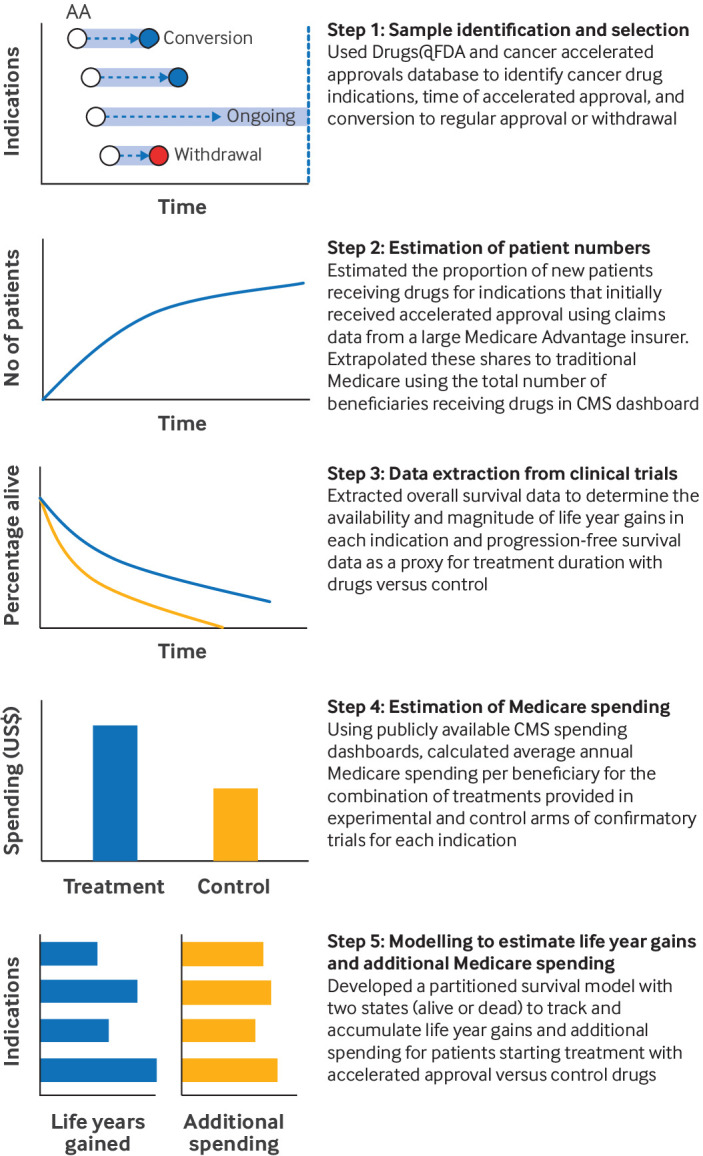
Overview of our methodological approach to evaluating survival gains and additional Medicare spending associated with early access to new cancer drugs through FDA accelerated approval pathway. AA=accelerated approval. CMS=Centres for Medicare & Medicaid Services

We implemented our methodological approach in five steps (figure 1). Firstly, we identified the cancer drug indications that initially received FDA accelerated approval and determined their current status: the drugs either remained under accelerated approval, were converted to regular approval, or had been withdrawn from the market. The period between accelerated approval and conversion to either regular approval or withdrawal represented the period of early access, during which a patient would have access to a drug which would not have been possible without the accelerated approval pathway (online supplemental appendix figure 1). Secondly, we estimated the number of patients who had gained access to new cancer drugs for indications that had initially received FDA accelerated approval. Thirdly, we determined whether cancer drug indications that had initially been granted accelerated approval had demonstrated overall survival benefit to patients compared with control treatments in clinical trials. We then collected Medicare spending data, and finally, we developed a decision analytical model to estimate life year gains to patients and additional Medicare spending incurred during the early access period for cancer drug indications that had initially received accelerated approval. In the model, patients receiving treatment with accelerated approval drugs could accrue survival benefits, and could also incur additional Medicare spending compared with patients receiving control treatments during the same period.

The study period began in 2012, the first year for which Medicare spending data were available. We considered drugs given accelerated approval up to 31 December 2020 to ensure that we could observe at least four years of follow-up data on overall survival from confirmatory trials on overall survival (up to 30 November 2024). Beneficiaries who gained early access to new cancer drug indications were followed up until 31 December 2022 (online supplemental appendix figure 2).

Because our analysis compared accelerated approval of cancer drug indications with regular approval, rather than evaluating a specific intervention, no single reporting guideline directly applied to our study. However, our reporting aligns with the Consolidated Health Economic Evaluation Reporting Standards 2022 (CHEERS 2022) statement.^34^

### Sample identification and selection

We identified cancer drug indications with FDA accelerated approval from 1 January 2012 to 31 December 2020 using the Center for Drug Evaluation and Research list of Drug and Biologic Accelerated Approvals based on a surrogate endpoint. Our sample included original indication approvals for new drugs and supplementary indication approvals for previously approved drugs. The status of accelerated approvals was determined using the Oncology Accelerated Approvals database maintained by the FDA Oncology Center of Excellence. We recorded whether cancer drug indications that initially received accelerated approvals had been verified for clinical benefit by the FDA and converted to regular approval, had been withdrawn from the market, or if confirmatory trials for these indications were ongoing as of 30 November 2024. If applicable, dates of conversion to regular approval or dates of withdrawal were recorded.

### Estimation of patient numbers

We estimated the number of patients who received new cancer drugs for indications that initially received FDA accelerated approval through conversion to regular approval, withdrawal from the market, or by 31 December 2020. Since 2012, the Centers for Medicare & Medicaid Services (CMS) has reported the total number of Medicare beneficiaries per year using individual drugs in part B insurance and part D .^35 36^ However, data for indication-specific use are not publicly accessible.

To estimate the proportion of Medicare beneficiaries who used new cancer drugs for indications granted FDA accelerated approval, we developed algorithms using claims data.^37 38^ We based our estimation of indication-specific use on Medicare Advantage plans offered by a large US commercial health insurer, and then extrapolated the proportion of use attributable to each indication for each drug to all Medicare claims, as in previous studies.^38^

We assumed that usage patterns of accelerated approval drugs are broadly similar between beneficiaries with Medicare Advantage and those with traditional Medicare. Although these populations differ by geographical distribution, race and ethnicity, income, and education level,^39^ earlier research has found no systematic differences between them in cancer prevalence or spending on injectable drugs.^40 41^

We used Optum Insights Clinformatics to extract the annual de-identified medical and pharmacy claims for each drug from 1 January 2012 to 31 December 2020. To link each patient to a specific indication, we identified the proportion of patients starting treatment with the drug for a given indication each year, which involved first distinguishing patients who were new to the treatment from those who had used the drug previously. For the subpopulation of new users, we then calculated indication-specific proportions using data from the first claim for each drug.

We based our algorithms to estimate indication-specific use on ICD-9 (international classification of diseases, 9th revision) and ICD-10 (10th revision) codes and label-indicated concomitant medications (defined as treatments used within 30 days of the dispensing of interest) (online supplemental appendix table 1). When multiple indications were present within the same set of ICD-9 and ICD-10 codes and concurrent treatments, we also used information on label-indicated prior therapies to identify indication-specific use. In instances where a single dispensing of a particular drug could be used to treat multiple indications, we attributed the drug’s use to the indication associated with an overall survival benefit to the patient. If no indication had an overall survival benefit, we attributed use to the indication with the earliest approval date, and tested the sensitivity of our findings to this assumption.

After calculating the proportion of users receiving new cancer drugs for indications that initially received FDA accelerated approval, we applied the proportion to the total numbers of Medicare beneficiaries receiving each drug in CMS data. For part D, we used the total number of beneficiaries, as reported by CMS. For part B, CMS data include only traditional Medicare. We therefore adjusted the CMS data for part B using publicly available enrolment proportion to account for Medicare Advantage coverage, similar to previous studies.^32^

For example, we estimated that in 2013, the first year with CMS data available for brentuximab vedotin, 85% of all patients prescribed this drug were new users, and 50% of those new users received it for an indication with accelerated approval. CMS reported that 645 Medicare beneficiaries received brentuximab vedotin in 2013; we estimated that 548 (85%) of these were new users, and 274 (50%) of these 548 patients were treated for the accelerated approval indication. Because this is a part B drug and CMS data reflect only traditional Medicare, we adjusted for the fact that traditional Medicare accounted for 72% of total Medicare enrolment in 2013. The adjusted denominator was therefore 896, and an estimate of 381 new patients used the drug for the accelerated approval indication.

Our stepwise approach was implemented in Stata (version 18) to estimate the total annual number of patients receiving new cancer drugs for indications that initially received FDA accelerated approval across all Medicare beneficiaries.

### Data extraction from clinical trials

For each indication, we reviewed section 14 (clinical studies) of the drug’s labelling approved by the FDA at the time of accelerated approval to identify the pivotal trial or trials supporting the FDA's decision. We then systematically reviewed approval letters in the Drugs@FDA database to identify post-marketing confirmatory trials requested at the time of accelerated approval, and their projected completion dates. We used national clinical trial (NCT) numbers to search ClinicalTrials.gov for each confirmatory trial and identify linked articles. When NCT numbers were unavailable, we searched ClinicalTrials.gov using the drug name and approved indication. We also searched company press releases and industry news outlets to identify confirmatory trials.

For indications that were converted to regular approval, we identified completed confirmatory trials by reviewing FDA approved labelling at the time of conversion to regular approval. We extracted information on treatment and control groups, study design, primary endpoint or endpoints, and results from pivotal trials and confirmatory trials. All data were independently extracted by two investigators and confirmed by a third.

We assessed the availability of overall survival results for each indication, and extracted data on the median duration of overall survival in treatment and control groups, hazard ratios, and 95% confidence intervals (CI), using the most recent results available from confirmatory trials. When the median was not reached in clinical trials, we extracted data on survival rates up to the latest available time point. When data on overall survival were not available in confirmatory trials, we used information available from pivotal trials. We categorised each indication based on whether it showed a statistically significant overall survival benefit, as in earlier studies.^42 43^ We also extracted data on progression-free survival using this same approach.

### Calculation of Medicare spending

We used publicly available prescription drug event data from the CMS drug spending dashboards to evaluate Medicare spending for cancer drug indications that were initially given FDA accelerated approval, as in earlier research.^33 44^ We calculated the difference in Medicare spending between the treatment strategy (including the accelerated approval drug) and the standard of care strategy (without the accelerated approval drug) as additional spending, as tested in the control arms of clinical trials.

We first identified the drugs included in the experimental and control arms of clinical trials. When only single arm trials were available, we assumed no available treatments in our base case analysis. For each drug, we extracted annual average spending per beneficiary using spending dashboards for either part B or part D. When drugs were listed in both parts B and D, we assigned them to either part B or part D following the approach used in previous studies.^32^ When spending data for different formulations of the same drug were available, we calculated a weighted average using annual beneficiary numbers. We adjusted spending for inflation to 2022 US dollars, based on the US Consumer Price Index for Medical Care.^45^

For each indication, we then separately summed up the average annual Medicare spending per beneficiary for all drugs in the experimental arms and those in the control arms. The difference between these sums represented the additional yearly spending allocated to drugs with earlier patient access.

### Modelling to estimate life year gains and additional spending

To estimate life year gains and additional spending associated with new cancer drug indications with FDA accelerated approval, we developed a partitioned survival model in Microsoft Excel with two states: alive and dead (online supplemental appendix figure 3).^46^ Partitioned survival models are often used to evaluate the costs and benefits of treatments for advanced and metastatic cancers.^47 48^ Our model incorporated monthly cohorts of patients starting treatment with either the accelerated approval drug or the usual standard of care, from the date of accelerated approval to the end of 2020, with patient follow-up extending to December 2022.

We tracked life year gains associated with the accelerated approval drug and the control treatment based on overall survival data extracted from clinical trials. We estimated the proportion of surviving patients over time by fitting an exponential curve to the median survival data, a pragmatic approach for long term data extrapolation in the absence of individual participant level data.^49 50^ The exponential distribution is widely used in economic evaluations of cancer treatments.^51 52^ We assumed proportional hazards throughout the model to reflect a constant treatment effect on patient survival.

To maximise use of the available data, the model incorporated the most recent overall survival data, irrespective of statistical significance. By multiplying the proportion of living patients by the total number of patients in each month, the model accumulated the months lived with the accelerated approval drug versus months lived with the control treatment.

Patients who were alive and receiving treatment, either with the accelerated approval drug or the control treatment, accrued additional spending in the Medicare programme. The model had a payer perspective, because we only considered Medicare spending in our study. The model estimated the proportion of patients remaining on treatment (the accelerated approval drug or the control treatment) over time by fitting an exponential curve to the progression-free survival data extracted from clinical trials. Given the retrospective nature of the analysis (estimating life year gains and costs up to December 2022), the model did not discount life year gains or additional spending.

We compared the estimated spending per life year gained against commonly used US cost effectiveness thresholds, which range from $100 000 (£75 991; €86 399) to $150 000 per quality adjusted life year (QALY) or equal value life year gained, as recommended by the Institute for Clinical and Economic Review.^53 54^ Since no formal threshold exists for life years gained, we use this range as a proxy for the amount a decision maker might be willing to pay for a drug with associated survival benefits.

To estimate uncertainty, we performed probabilistic sensitivity analyses using Monte Carlo simulations with 10 000 iterations per drug indication. Overall survival and progression-free survival parameters were modelled using normal distributions, while costs were modelled using a gamma distribution. Results were reported as 95% CIs.

### Scenario analyses

To assess the robustness of our findings against the data limitations, we performed several scenario analyses. The scenario analyses included varying comparator treatments for indications supported by single arm trials or trials with multiple comparators (which affected estimated accrued additional spending), reallocating drug use across similar indications to account for limitations in the claims data (which influenced both Medicare spending and patient survival outcomes), and modelling patient survival benefits in the absence of overall survival data (which affected estimated life years gained). We also varied the period of time that constituted early access, which we hypothesised would have the greatest impact on our results, as this assumption applied to all indications in our sample.

In the first set of scenario analyses, we tested the sensitivity of our findings to different spending estimates, accounting for the various drugs included in the treatment and control arms of clinical trials. In the base case analysis, we assumed no relevant comparator treatment when a drug was evaluated in a single arm trial. In the analysis (spending scenario 1), we identified potentially relevant comparators by searching ClinicalTrials.gov for the drug name and its accelerated approval indication, and by assessing FDA medical review documents available on the Drugs@FDA data repository for references to existing alternative treatments. Furthermore, we varied the additional spending for the treatment and control arms by creating high cost (spending scenario 2) and low cost (spending scenario 3) scenarios, especially when the control arm allowed for physician's choice.

In the second set of analyses, we allocated drug use to different indications when our algorithms (based on claims data) could not clearly distinguish between a drug's similar indications. For example, nivolumab had three accelerated approvals for metastatic melanoma. In our base case, we allocated all observed nivolumab use to the indication with the most substantial overall survival gain in patients. In scenario analyses (utilisation scenarios 1 and 2), we tested the impact of allocating the observed drug use to indications with less substantial survival gains.

In the third set of scenario analyses, we adopted different assumptions about the survival benefits in indications with no overall survival data. In our base case analysis, we assumed patients who received drugs for indications that had no overall survival data (eg, owing to the the single arm trial design) derived no life year gains from earlier access. When data on overall survival were not available, we identified existing systematic reviews of trial level meta-analyses that evaluated the association between the surrogate marker(s) used as the primary efficacy endpoint(s) in key trials or confirmatory trials, and overall survival for the same clinical indication.^17 18^ When available, we used linear regression equations reported in trial level meta-analyses to estimate the magnitude and statistical significance of overall survival results, using available data from surrogate endpoints (survival scenario 1). In a separate analysis, we assumed that the median overall survival benefit of indications with verified clinical benefit applied to similar indications still undergoing confirmatory trials (survival scenario 2).

In the fourth set of scenario analyses, we varied the definition of early access to reflect the median duration of a confirmatory trial until its completion (duration of early access scenario 1)^55^ and the time until the original projected completion date of a confirmatory trial (duration of early access scenario 2).

### Patient and public involvement

No members of the public were formally involved in the design or implementation of this study, because no funding was available for public or patient involvement. However, we have discussed our findings with members of the National Breast Cancer Coalition, a network of patient organisations from across the US interested in improving the FDA’s regulation of pharmaceuticals. We have included their comments about the policy and practice implications of our findings in the discussion. Results will be shared via the press and public policy offices at the London School of Economics and Political Science, Harvard Pilgrim Health Care Institute, University of York, Peking University, and Yale University. Typical mediums include press releases, social media posts (X, Bluesky, and LinkedIn), and policy briefs for policy makers. We will continue engaging with members of the National Breast Cancer Coalition to develop and publish a plain language summary of the study and its implications for the public and policy makers.

## Results

### Sample characteristics

Between 2012 and 2020, the FDA granted accelerated approval for 97 cancer drug indications. We excluded seven of these indications for which Medicare spending data were unavailable, corresponding to five drugs, giving a final study sample of 90 indications. Of the 90 indications, 15 (16.7%) were for lymphoma, 13 (14.4%) for lung cancer, nine (10.0%) for leukaemia, eight (8.9%) for urothelial carcinoma, and six (6.7%) for breast cancer (table 1, online supplemental appendix table 1).

**Table 1:** Characteristics of cancer drug indications included in study

Indications (n=90)	No (%)
**Accelerated approval indication**
Lymphoma	15 (16.7)
Lung cancer	13 (14.4)
Leukaemia	9 (10.0)
Urothelial carcinoma	8 (8.9)
Breast cancer	6 (6.7)
Melanoma	6 (6.7)
Multiple myeloma	5 (5.5)
Hepatocellular carcinoma	4 (4.4)
Other*	24 (26.7)
**Accelerated approval status**
Verified†	51 (56.7)
Ongoing	23 (25.6)
Withdrawn	16 (17.8)
**Availability of overall survival benefit**
Evidence of overall survival benefit	27 (30.0)
No evidence of overall survival benefit	30 (33.3)
Unknown	33 (36.7)

Percentages may not add up to 100% owing to rounding.

* Includes: cervical cancer (n=1), cholangiocarcinoma (n=1), colorectal cancer (n=3), endometrial carcinoma (n=2), gastric or gastro-oesophageal junction adenocarcinoma (n=1), head and neck squamous cell carcinoma (n=1), Merkel cell carcinoma (n=2), ovarian cancer (n=2), prostate cancer (n=1), sarcoma (n=2), Kaposi's sarcoma (n=2), thyroid cancer (n=2), and tumour agnostic cancers (n=4). Online supplemental appendix includes the list of cancer drug indications with accelerated approvals.

† Cancer drug indications judged to have verified clinical benefit by the US Food and Drug Administration and converted to regular approval.

Among the 90 indications initially given FDA accelerated approval, 51 (56.7%) were converted to regular approval, 16 (17.8%) were withdrawn from the market, and 23 (25.6%) had post-marketing requirements for ongoing confirmatory trials and remained under accelerated approval (table 1).

Overall, 27 (30.0%) of 90 indications that initially received FDA accelerated approval demonstrated an overall survival benefit to patients in randomised controlled trials (online supplemental appendix table 2 and 3). Of the 51 indications converted to regular approval, 25 had evidence of overall survival benefit to patients, while the remaining 26 were converted to regular approval based on surrogate endpoints: 15 indications showed no overall survival benefit and the overall survival benefit of the remaining 11 indications was unknown (six due to single arm trial designs and five due to immature survival data). Two indications remained under accelerated approval that had evidence of overall survival benefit. Randomised controlled trials found evidence of no overall survival benefit for 30 (33.3%) indications, including 11 that were converted to regular approvals. Overall survival benefit was unknown for 33 (36.7%) indications: 11 because of single arm trial designs, 9 because of immature survival data, and the remaining 13 because of other reasons.

### Use of cancer drugs

**Figure 2:**
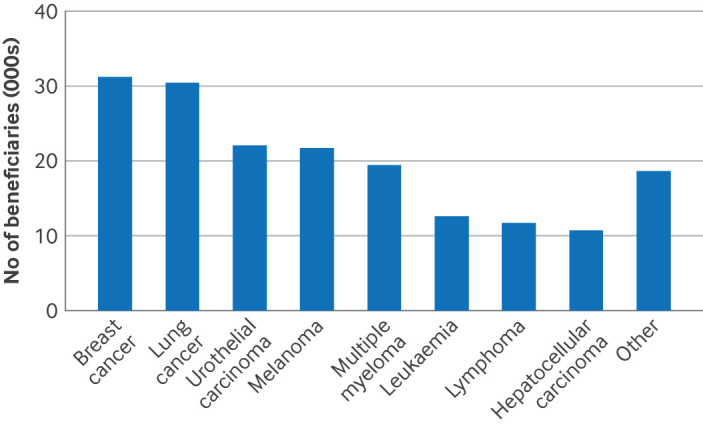
Estimated number of Medicare beneficiaries who accessed new cancer drugs for indications that initially received US Food and Drug Administration accelerated approval (by cancer type) and used the new drugs in the period from accelerated approval to conversion to regular approval, withdrawal, or 31 December 2020. Other=cervical cancer; cholangiocarcinoma; colorectal cancer; endometrial carcinoma; gastric or gastro-oesophageal junction adenocarcinoma; head and neck squamous cell carcinoma; Merkel cell carcinoma; ovarian cancer; prostate cancer; sarcoma; Kaposi's sarcoma; thyroid cancer; and tumour agnostic cancers

An estimated 178 708 Medicare beneficiaries received early access to new cancer drugs through the FDA's accelerated approval pathway. Overall, an estimated 31 245 (17.5%) beneficiaries received drugs for breast cancer, 30 501 (17.1%) for lung cancer, 22 098 (12.4%) for urothelial carcinoma, 21 695 (12.1%) for melanoma, and 19 455 (10.9%) for multiple myeloma (figure 2).

**Figure 3:**
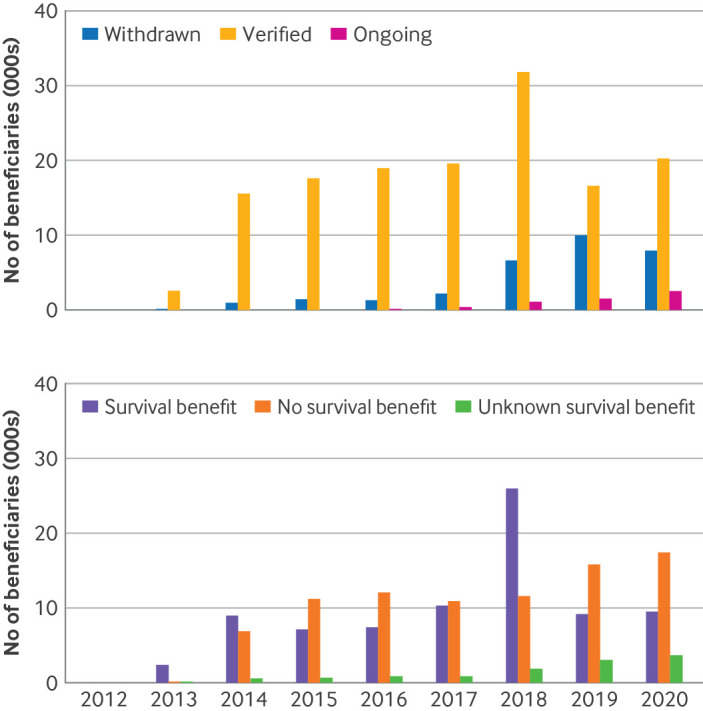
Estimated number of Medicare beneficiaries who accessed new cancer drugs for indications that initially received US Food and Drug Adminstration (FDA) accelerated approval according to (top) accelerated approval status and (bottom) availability of overall survival benefit. Verified=cancer drug indications with FDA verified clinical benefit and converted to regular approval. Nearly half of these indications did not have evidence of overall survival benefit to patients

A total of 142 760 (79.9%) beneficiaries received drugs for indications that were ultimately converted to regular approval, 5574 (3.1%) for indications still under accelerated approval, and 30 374 (18.0%) for indications that were later withdrawn. The number of beneficiaries receiving treatment for indications that were withdrawn has increased in recent years, comprising 25.8% of beneficiaries with early access to cancer drugs in 2020 (figure 3).

Among all beneficiaries who received the new cancer drugs for accelerated approval indications, 80 885 (45.3%) received them for indications that were eventually shown to have overall survival benefit, whereas 86 085 (48.2%) received the new drugs for indications with no overall survival benefit, and 11 738 (6.5%) for indications where overall survival benefit was unknown. In 2020, 17 455 beneficiaries received drugs for indications that had no overall survival benefit, nearly twice as many as the 9517 beneficiaries who received drugs for indications that did show an overall survival benefit (figure 3).

### Life year gains and additional spending

**Figure 4:**
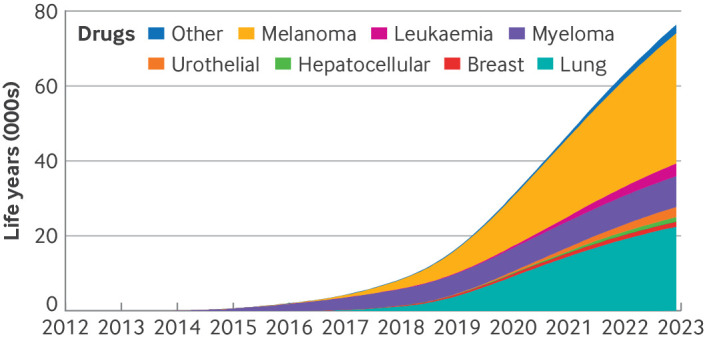
Estimated cumulative life year gains to Medicare beneficiaries who used new cancer drugs for indications initially given accelerated approval by the US Food and Drug Administration, by cancer type. Lymphoma indication category is not shown, because new drug use in lymphoma indications resulted in no life year gains but resulted in life year losses to patients

Our model, which applied trial data to Medicare drug use, estimated that early access to cancer drugs for indications that initially received accelerated approval resulted in 76 164 (95% CI 71 520 to 80 658) life years gained by Medicare beneficiaries between 2012 and 2022. Life year gains in patients with melanoma accounted for an estimated 34 965 (95% CI 28 425 to 42 497; 45.5%) of the total number of life years gained by Medicare beneficiaries, followed by patients with lung cancer (22 286 life years; 95% CI 21 282 to 22 765; 29.4%), and patients with multiple myeloma (8159 life years; 95% CI 7929 to 8220; 10.8%; figure 4). The use of nivolumab to treat melanoma, pembrolizumab to treat non-small cell lung cancer, and pemetrexed in combination with pembrolizumab and carboplatin for non-squamous, non-small cell lung cancer accounted for 52 107 (68.4%) of life years gained by beneficiaries.

**Figure 5:**
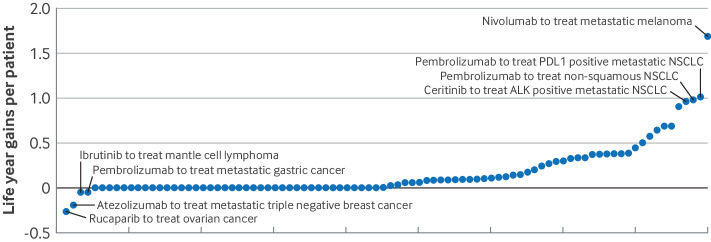
Estimated life year gains per patient owing to early access to new cancer drugs for 90 indications initially given accelerated approval by the US Food and Drug Administration. Cancer drug indications are ordered by estimated mean life year gains per patient. NSCLC=non-small cell lung cancer. ALK=anaplastic lymphoma kinase

Between 2012 and 2022, nearly half (n=41, 45.6%) of new cancer drug indications initially given accelerated approval resulted in no life year gains to patients (figure 5). Thirty five (38.9%) indications resulted in estimated life year gains from 0.01 to 0.5 years per patient, 10 (11.1%) indications gave life year gains of 0.5 years or more per patient, and four resulted in life year losses to the patient.

**Figure 6:**
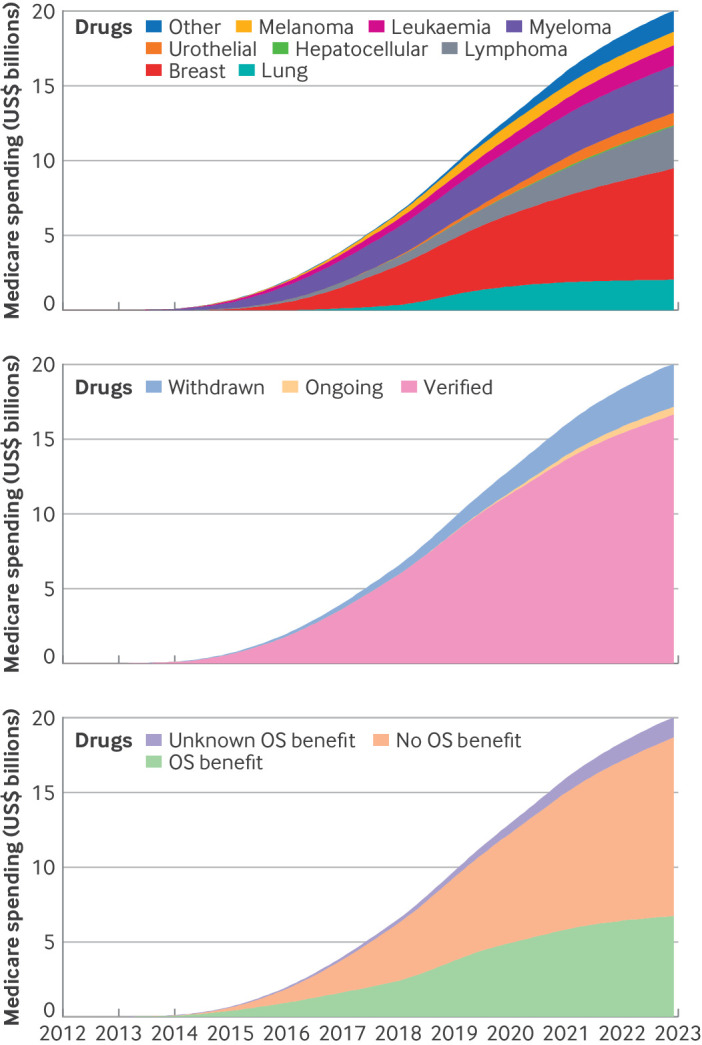
Estimated additional Medicare spending for new cancer drugs for indications that initially received accelerated approval by the US Food and Drug Administration, according to (top) cancer type, (middle) accelerated approval status, and (bottom) availability of overall survival benefit. OS=overall survival

Use of these cancer drugs was estimated to cost the Medicare programme $20.1bn (95% CI $12.9bn to $30.2bn) in additional spending, compared with standard of care treatment (comparator treatments listed in online supplemental appendix table 4). Additional Medicare spending for drugs indicated for breast cancer accounted for $7.4bn ($4.8bn to $10.6bn, 36.8%) of the total additional spending, followed by $3.1bn ($2.0bn to $4.5bn, 15.4%) for multiple myeloma, $2.8bn ($1.8bn to $4.1bn, 13.9%) for lymphoma, $2.0bn ($1.3bn to $3.0bn, 9.9%) for lung cancer, and $1.4bn ($0.9bn to $1.9bn, 6.9%) for leukaemia (figure 6).

An estimated $16.6bn (95% CI $10.7bn to $25.1bn, 82.6%) of the total additional spending was attributable to indications that were converted to regular approval, while $2.9bn ($1.9bn to $4.2bn, 14.4%) was for indications that were later withdrawn from the market (figure 6). Early access to cancer drugs in indications demonstrated to result in overall survival benefits to patients accounted for an estimated $6.7bn ($4.3bn to $10.1 bn, 33.3%) of additional Medicare spending (figure 6). The remaining additional spending was for indications with no overall survival benefit to patients ($11.9bn, $7.7bn to $17.8bn, 59.2%) or indications with unknown overall survival benefit to patients ($1.4bn, $0.9bn to $2.2bn, 7.0%).

**Figure 7:**
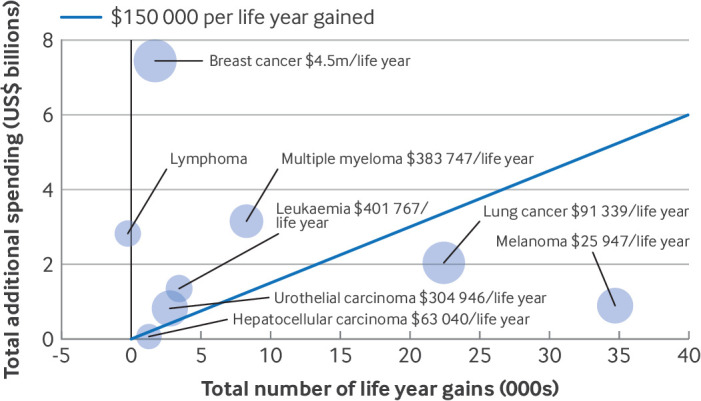
Estimated additional Medicare spending and life year gains, by cancer type. Node size is proportional to the number of Medicare beneficiaries receiving access to new cancer drugs for indications that initially received accelerated approval. Blue diagonal line from 0 indicates $150 000 per life year gained, which is a conventional cut-off value used to define cost effectiveness of healthcare spending in the US; results below the line require spending of less than $150 000 per life year gained, and are therefore considered more cost effective. Results for lymphoma indications suggest that additional Medicare spending did not provide life year gains but correlated with potential loss of life

Our model estimated the additional Medicare spending per life year gained to be $263 371 (95% CI $180 139 to $373 884), ranging from an additional $25 947 ($18 174 to $39 829) per life year gained for drugs with melanoma indications to $4.5m ($3.1m to $12.2m) per life year gained for drugs with breast cancer indications (figure 7). The additional Medicare spending for lymphoma drug indications ($9.5m, $5.1m to $18.9m) was not associated with life year gains but was associated with a potential loss of life. Drug indications that were converted to regular approval resulted in an additional cost of $221 545 ($153 589 to $311 417) per life year gained.

### Scenario analyses

**Figure 8:**
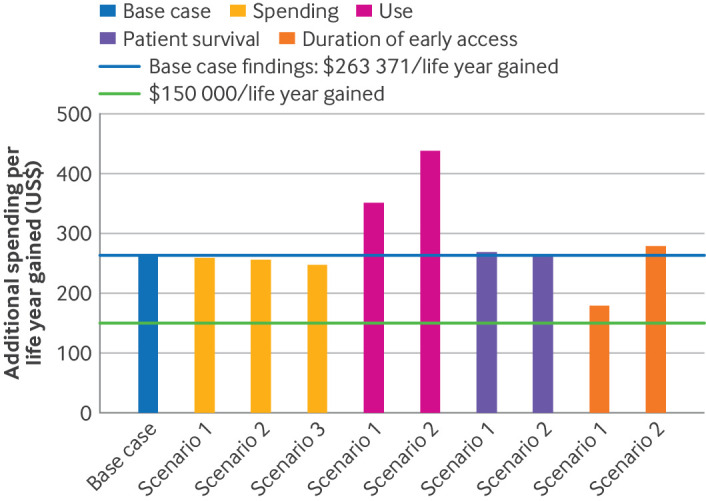
Sensitivity of results on additional Medicare spending per life year gained to different assumptions and scenarios

Figure 8 shows the sensitivity of our findings to different assumptions and scenarios. Our findings remained stable across a range of assumptions. Allocating the observed drug use to indications with less substantial survival gains led to higher estimated Medicare spending per life year gained. Capping the duration of early access to 17 months, the median duration of a confirmatory trial, resulted in an estimated cost to Medicare of $178 728 per life year gained.

## Discussion

### Principal findings

Between 2012 and 2020, the FDA's accelerated approval pathway enabled an estimated 178 708 Medicare beneficiaries to gain earlier access to 90 cancer drug indications. This early access was associated with an estimated 76 164 additional life years gained by patients. However, these gains came at a substantial cost: approximately $20.1bn in additional Medicare spending. On average, the additional cost to Medicare was $263 371 per life year gained, substantially more than conventional cost effectiveness thresholds in the US healthcare system.^53 54^

Most beneficiaries received cancer drugs for indications that were eventually converted to regular approval by the FDA; these indications accounted for 79.9% of observed use and 82.6% of additional Medicare spending. Although most early access drug use and additional spending were for indications that were ultimately converted from accelerated approval to regular approval by the FDA, the FDA often converted accelerated approvals to regular approvals without evidence of overall survival benefit to patients, consistent with findings from earlier studies.^22 23^ During our study period, only 25 of 51 converted indications had evidence of overall survival benefit.

We found a growing trend in the use of drugs for indications that were later withdrawn following negative confirmatory trials. This trend might reflect declining standards for evidence quality at the time of accelerated approval, delays in completing confirmatory trials, or delays in regulatory action. From 2018 to 2020, about 25% of new cancer drug indications to which patients were given early access were eventually withdrawn. Overall, most access to new cancer drugs through the accelerated approval pathway were in indications that did not demonstrate evidence for a survival benefit to patients within at least four years after approval.

When Medicare beneficiaries derived survival gains from earlier access to new cancer drugs with accelerated approval, the benefit they gained was often small. Only 10 indications resulted in life year gains of six months or more per patient. Three factors determined the magnitude of life year gains: the availability of evidence of overall survival benefit to patients, the length of the period between an indication being given accelerated approval and either conversion to regular approval or withdrawal from the market, and the number of beneficiaries receiving the treatment during that period. When a drug provided substantial survival benefit to patients, a short interval between accelerated approval and regular approval limited the number of patients who could access the drug earlier than they would have under the standard approval pathway, thereby limiting the life year gains attributable to accelerated approval. Conversely, when a drug did not give survival benefit to patients but remained on the market for a long period before conversion or withdrawal, more patients received the drug without deriving survival benefit. Our findings in relation to new drug indications to treat breast cancer illustrate this: despite three of six breast cancer indications having evidence of overall survival benefit to patients, the average time to conversion or withdrawal was considerably longer for indications without survival benefit to patients (36.4 months) compared to indications with survival benefit (16.3 months). As a result, most instances of early access occurred in indications without survival benefit to patients, resulting in few life year gains attributable to accelerated approval.

Early access through accelerated approval yielded uneven survival outcomes, and Medicare incurred substantial costs for treatments that, in some cases, did not deliver meaningful clinical benefit to beneficiaries. The variation in survival gains across indications meant that substantial Medicare spending sometimes provided patients with minimal or no life year gains. For example, an estimated 31 245 Medicare beneficiaries who received breast cancer drugs between accelerated approval and either conversion to regular approval, withdrawal from the market, or December 2020, gained only 1648 life years by the end of 2022 at an additional cost of $7.4bn, resulting in an additional cost to Medicare of $4.5m per life year gained. These findings raise important questions about the value Medicare beneficiaries receive from taxpayer investments and premiums paid by beneficiaries in cancer drug indications approved through the accelerated approval pathway. Furthermore, resources allocated to treatments with uncertain or limited benefit to patients may divert funding from more effective interventions, or contribute to higher premiums and out-of-pocket costs for beneficiaries, which can have adverse consequences on their health.

### Policy and practice implications

The FDA's accelerated approval pathway was established to provide earlier access to treatments for patients with serious or life threatening conditions, particularly when no alternative treatment options exist. The pathway was designed to balance the potential benefits of earlier treatment access against the risks of clinical uncertainty associated with the use of surrogate endpoints in clinical trials^4^, allowing people to access treatments sooner than they would under traditional approval processes.^26 56^ While accelerated approval can lead to the use of treatments that later prove to be ineffective or only marginally effective, some patients with advanced cancers may choose to accept this uncertainty; waiting for complete information on a drug’s clinical benefit may harm patients because of delayed access to potentially effective treatments.^57^ However, individuals vary in their preferences: many patients express a strong desire for certainty that a cancer drug will improve their chance of survival, and some are willing to wait longer for that evidence.^58^ Balancing the risks of clinical uncertainty with the potential harms of delayed access to treatment remains central to the accelerated approval pathway’s rationale.^24 59^

A fundamental condition of this pathway is the timely completion of trials after a drug has been given accelerated approval to resolve clinical uncertainty. Our findings suggest that limiting the duration of post-approval trials would better align Medicare spending associated with treatments that offer proven clinical benefit to beneficiaries. Recent initiatives encourage pharmaceutical manufacturers to have confirmatory trials underway at the time of accelerated approval,^60^ which is associated with shorter times to conversion or withdrawal.^61 62^ However, our findings indicate that timely conversion to regular approval is insufficient if it is based on weak clinical evidence. During our study period, most indications were converted without demonstrating an overall survival benefit to patients.

Surrogate endpoints used to grant accelerated approval should have a reliable and strong association with overall survival benefit, ideally evaluated using meta-analysis methods.^63^ Bivariate meta-analysis, which accounts for the correlation between treatment effects and uncertainty around that relationship, is the preferred approach.^64^ In addition, confirmatory trials should measure overall survival whenever feasible and avoid features that may compromise survival assessment (eg, treatment switching).^43^ Despite a growing interest in using real world evidence for regulatory decision making, existing claims and electronic health record systems are not sufficient to achieve this,^65^ and findings from non-randomised studies may differ from those of randomised trials in unpredictable ways.^66^

The FDA could further strengthen the accelerated approval pathway by improving communication about clinical uncertainty. The agency should explicitly disclose both the nature and magnitude of the uncertainty associated with treatments without verified clinical benefits and the use of surrogate endpoints in trials. Currently, FDA approved labelling for drugs, including those granted accelerated approval, often does not clearly communicate this uncertainty, limiting clinicians' and patients' ability to make fully informed treatment decisions.^67^

Beyond the FDA, other stakeholders can encourage the timely generation of robust evidence for drugs given accelerated approval.^68^ Firstly, drug prices should reflect the scale of clinical uncertainty:^29^ currently, no consistent association exists between a drug’s price and its demonstrated clinical benefit.^69^ Even when confirmatory trials report improvements in overall survival, prices for cancer drugs granted accelerated approval do not increase accordingly, thus pharmaceutical companies are not financially incentivised to provide evidence on overall survival.^70^ CMS could implement reimbursement policies that lower payment rates until drugs demonstrate clinical benefit, thereby encouraging drug manufacturers to conduct rigorous confirmatory trials.^71 72^ Secondly, clinical practice guidelines should communicate the uncertainty of evidence supporting accelerated approvals more effectively. At present, guidelines quickly incorporate recommendations for drugs with accelerated approval indications, facilitating their rapid uptake in clinical practice.^73 74^ However, these guidelines do not always indicate when there is prolonged uncertainty about a drug's benefit to patients, nor when a drug indication has been withdrawn,^73 75^ which can lead to continued patient exposure to ineffective treatments and undermine trust in clinical recommendations.

These challenges are not confined to the US healthcare system, and regulatory decisions made by the FDA often influence drug availability and clinical practice in other countries. Our findings have implications beyond the US;^76 77^ drugs given FDA accelerated approval may also receive marketing authorisation by regulatory agencies in other settings, but without similar requirements for confirmatory trials after the drug is marketed. For example, some indications that initially received FDA accelerated approval may not receive conditional marketing authorisation by the European Medicines Agency and therefore may not be required to verify clinical benefit in the same way.^78^ As a result, clinical uncertainty may persist for longer periods in settings outside of the US, further complicating decisions to fund and use these new treatments in other health systems.^79^ In some cases, drug indications withdrawn in the US following confirmatory trials with negative results remain available elsewhere.^80^

### Comparison with other studies

Earlier studies have described the characteristics of pivotal trials and confirmatory trials,^15 25^ evaluated the duration until completion of confirmatory trials (and subsequent conversion to regular approval or withdrawal),^12^ ^14^ documented overall survival benefits,^16 22 23^ and quantified spending associated with accelerated approvals.^33 37 44 81 82^ Our study offers a novel contribution to the literature by bringing together these strands and building on previous analyses to provide a comprehensive evaluation of the accelerated approval pathway for cancer drugs.

Our findings differ from those of a recent industry sponsored study, which estimated that early access to cancer drugs resulted in an estimated 263 000 life year gains in the US.^83^ This study focused on a selected subset of accelerated approvals after excluding 47% of eligible indications, estimated patient numbers using a proprietary database without providing details, identified overall survival benefits using data from randomised controlled trials and non-randomised studies (which may differ in their findings in unpredictable ways),^66 84^ and did not consider cost implications to Medicare.

### Limitations

Our analysis had several limitations. Firstly, we estimated the proportion of patients receiving treatment with cancer drugs for accelerated approval indications using data from Medicare Advantage plans managed by a large health insurer. While previous research has found no systematic differences in cancer prevalence or treatment strategies between Medicare Advantage and traditional Medicare populations, including spending on injectable drugs,^40 41^ important differences remain that may affect treatment patterns.^85^ For example, a recent study suggested that use of expensive cancer drugs may be lower in Medicare Advantage for indications with inexpensive treatment alternatives.^86^ Although this is unlikely to substantially affect our estimates (accelerated approval indications involve novel treatments for which cheaper alternatives are often unavailable), the findings may not be fully generalisable to the broader Medicare population. Additionally, reliance on data from a single insurer may further limit generalisability. Nonetheless, our approach is consistent with earlier studies that use the same nationwide data source.^38^

Secondly, extrapolating the patient survival data relied on the exponential distribution. While this approach is standard in economic evaluations of treatments for advanced cancers,^51 52 83^ alternative parametric distributions may provide a better fit to the underlying data.^87^ However, applying such alternatives would require individual participant level data and clinical validation, which were not available.^88^ In many cases, even Kaplan-Meier curves were not available (eg, when the most recent overall survival data were extracted from ClinicalTrials.gov). Additionally, the model adopted a limited time horizon, which may have resulted in an underestimation of total life years gained.

Thirdly, in our calculation of additional Medicare spending associated with early access to new cancer drugs, we assumed that the treatment strategies tested in clinical trials represented those used in clinical practice.

Our assessment of patient benefit was limited to overall survival, and we did not consider potential quality of life improvements for drug indications that did not show a survival benefit to patients. Previous research has shown that quality of life improvements are rare,^89^ with fewer than 5% of cancer drugs approved by the FDA between 2000 and 2020 offering quality of life benefits to recipients.^90^

Furthermore, we may have overestimated the patient survival benefits of the accelerated approval treatments. Our model assigned life year gains to beneficiaries if overall survival data numerically favoured the accelerated approval drug, irrespective of the statistical significance of the findings.

Lastly, our assessment did not account for potential harms associated with exposure to drug indications without verified benefits or indications that were subsequently withdrawn from the market.^28^ Medicare beneficiaries are aged 65 or older and have greater medical complexity, and may be at greater risk of harm from treatment related toxicities.^91^ We also did not consider non-drug spending, such as costs related to testing, administration, patient monitoring, other service use, and adverse events, which can be substantial for the Medicare programme. Costs incurred by patients were also not considered.^92^

## Conclusion

From 2012 to 2020, nearly 200 000 Medicare beneficiaries received new cancer drugs during the period between accelerated approval and either conversion to regular approval or withdrawal from the market. More than half of beneficiaries received the new cancer drugs for indications without overall survival benefits, despite some being converted to regular approval, exposing many patients to drugs with limited or unverified clinical benefit. Early access to new cancer drugs through the accelerated approval pathway accounted for an estimated $20bn in additional Medicare spending, but most beneficiaries derived minimal or no survival gains on this pathway, with only three drug indications accounting for more than two thirds of the total life years gained. These findings highlight the trade-off between earlier access to new treatments and substantial clinical uncertainty at considerable cost. Strengthening evidence standards to enable timely completion of confirmatory trials may help ensure that early access delivers meaningful benefit to patients, Medicare beneficiaries, and American taxpayers.

## Supplementary material

10.1136/bmjmed-2025-001934 online supplemental file 1

## Data Availability

Data are available upon reasonable request. Data may be obtained from a third party and are not publicly available.
