# Supplementary material for: Costs and benefits of early access to new cancer drugs through the US Food and Drug Administration’s accelerated approval pathway: retrospective observational study and economic evaluation
Source: BMJ Med. 2025 Dec 16;4(1):e001934. doi: 10.1136/bmjmed-2025-001934 (PMC12718571; doi:10.1136/bmjmed-2025-001934)
Supplement: online supplemental file 1 [file bmjmed-4-1-s001.pdf]

Costs and Benefits of Early Access to New Cancer Drugs through the U.S. Food and Drug Administration Accelerated Approval

Huseyin Naci, Mahnum Shahzad, Peter Murphy, Yichen Zhang, Rebecca Costa, Joseph S. Ross, Anita K. Wagner

Appendix

Table of contents

Appendix figure 1. Methodological approach. 3
Appendix figure 2. Timeline for identification of study sample and modelling of outcomes. 4
Appendix figure 3. Overview of the modeling approach. 5
Appendix table 1. List of accelerated approval indications included in the analysis. 6
Appendix table 2. Characteristics of confirmatory trials. 17
Appendix table 3. Available data on overall survival. 33
Appendix table 4. List of drugs and comparators considered for the calculation of Medicare spending. 47
CHEERS 2022 Checklist 59

Appendix figure 1. Methodological approach.

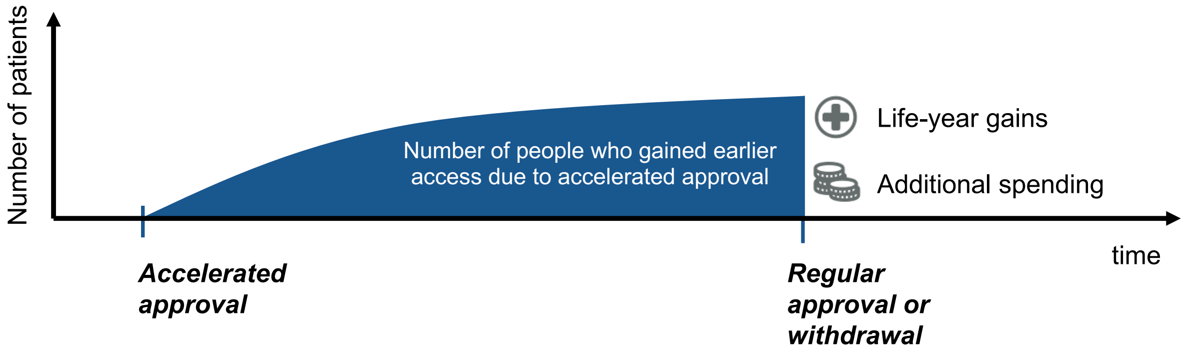

Our analysis focused on the period between accelerated approval and either conversion to regular approval or withdrawal, which represented the period of early access, which would not have been possible without the accelerated approval pathway. We estimated the life-year gains and additional spending for Medicare beneficiaries who gained access to drugs during this time for indications that initially received accelerated approval by the Food and Drug Administration (FDA).

Appendix figure 2. Timeline for identification of study sample and modelling of outcomes.

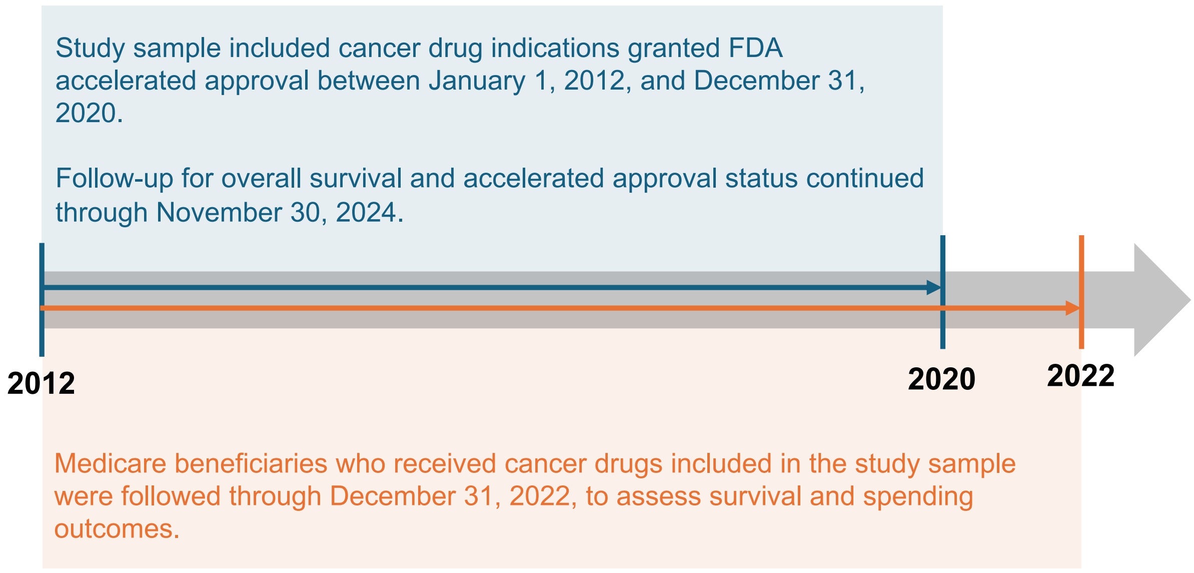

Appendix figure 3. Overview of the modeling approach.

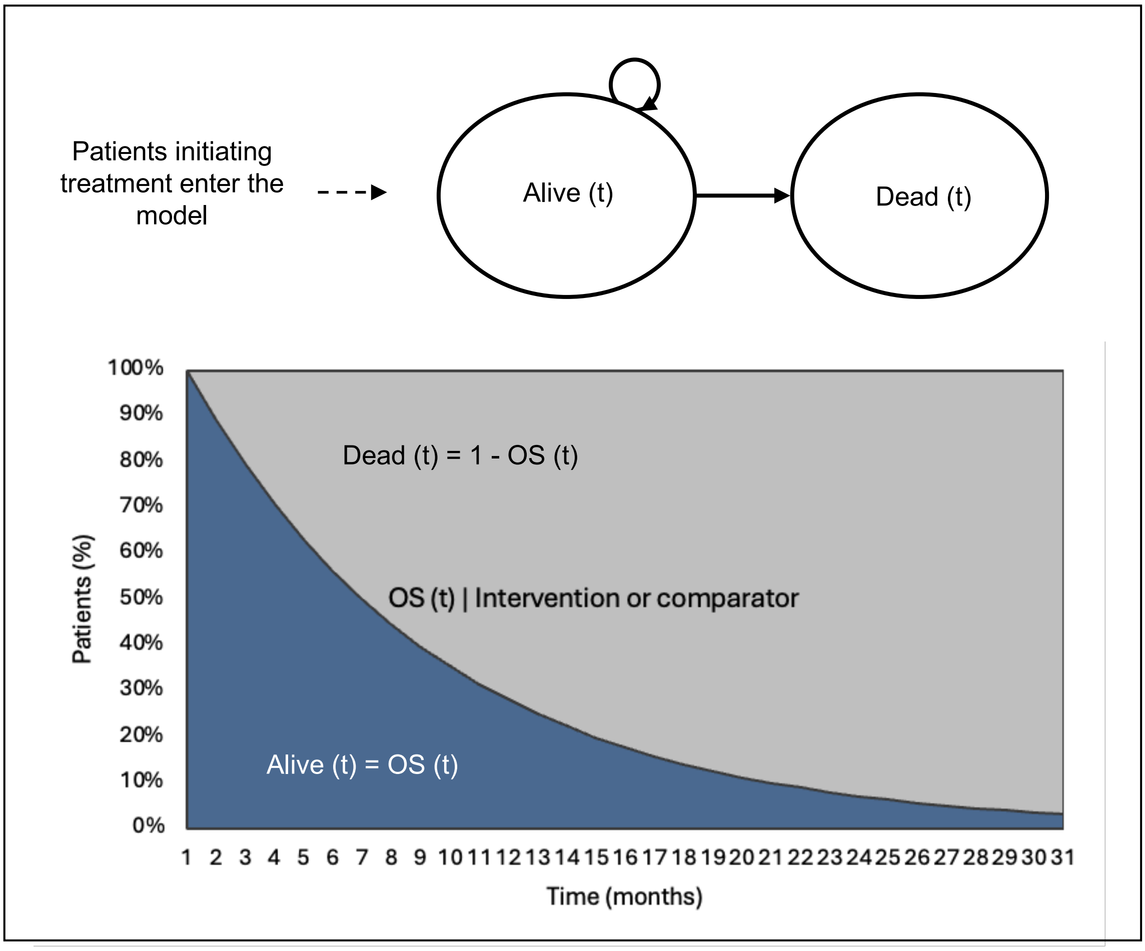

Partitioned survival modeling is commonly used in progressive diseases like cancer. In a partitioned survival model, a cohort of patients initiating therapy with the treatment of interest (and control) is tracked over time as they transition between mutually exclusive and exhaustive health states (e.g., alive and dead). The model estimates the proportion of patients in each state using a set of parametric survival equations. It utilises endpoints collected in clinical trials, such as data on overall survival. In the figure above, the cohort is divided into two sections: patients above the overall survival curve, who have died, and those below it, who are still alive.

Appendix table 1. List of accelerated approval indications included in the analysis.

ID	Drug name	Indication	Algorithm to identify patients	Accelerated approval date	Status	Conversion or withdrawal date
1	pembrolizumab	Metastatic small-cell lung-cancer (SCLC) with disease progression on or after platinum-based chemotherapy and at least one other prior line of therapy.	ICD 10: C34 (*assumed 15% of use attributable to SCLC*)	6/17/2019	Withdrawn	3/30/2021
2	atezolizumab	In combination with paclitaxel protein-bound for unresectable locally advanced or metastatic triple-negative breast cancer whose tumors express PD-L1 (PD-L1 stained tumor-infiltrating immune cells of any intensity covering = 1% of the tumor area), as determined by an FDA-approved test.	ICD 10: C50<br>ICD 9: 174<br><br>Use of paclitaxel 30 days within dispensing	3/8/2019	Withdrawn	10/6/2021
3	duvelisib	Treatment of adult patients with relapsed or refractory follicular lymphoma (FL) after at least 2 prior systemic therapies.	ICD 10: C82	9/24/2018	Withdrawn	12/17/2021
4	nivolumab	Metastatic small-cell lung cancer (SCLC) with progression after platinum-based chemotherapy and at least one other line of therapy.	ICD 10: C34 (*assumed 15% of use attributable to SSLC*)	8/16/2018	Withdrawn	12/29/2020
5	pembrolizumab	For patients with recurrent or locally advanced or metastatic gastric or gastroesophageal junction (GEJ) adenocarcinoma whose tumors express PD-L1 [CPS ≥1] as determined by an FDA-approved test, with disease progression on/after two or more prior lines of therapy including fluoropyrimidine and platinum containing chemotherapy and if appropriate, HER2/NEU targeted therapy.	ICD 10: C16<br><br>No concurrent use of trastuzumab, fluoropyrimidine- and platinum-containing chemotherapy	9/22/2017	Withdrawn	2/4/2022
6	nivolumab	Hepatocellular carcinoma previously treated with sorafenib.	ICD 10: C22.0<br><br>No concurrent use of ipilimumab	9/22/2017	Withdrawn	7/23/2021

ID	Drug name	Indication	Algorithm to identify patients	Accelerated approval date	Status	Conversion or withdrawal date
7	durvalumab	Locally advanced or metastatic urothelial carcinoma that progressed during or following platinum-containing chemotherapy or within 12 months of neoadjuvant or adjuvant treatment with platinum-containing chemotherapy.	ICD 10: C67<br>ICD 9: 188	5/1/2017	Withdrawn	2/19/2021
8	atezolizumab	Patients with locally advanced or metastatic urothelial carcinoma (mUC) who are not eligible for cisplatin-containing chemotherapy and whose tumors express PD-L1 as determined by an FDA approved test or who are not eligible for any platinum-containing chemotherapy regardless of PD-L1 status.	ICD 10: C67<br>ICD 9: 188<br><br>No prior or later platinum therapy	4/17/2017	Withdrawn	12/2/2022
9	ibrutinib	Adult patients with marginal zone lymphoma (MZL) who require systemic therapy and have received at least one prior anti-CD20-based therapy.	ICD 9: 200.3<br>ICD 10: C88.4	1/18/2017	Withdrawn	5/18/2023
10	olaratumab	In combination with doxorubicin for adults with soft tissue sarcoma with a histologic subtype for which an anthracycline-containing regimen is appropriate and which is not amenable to curative treatment with radiotherapy or surgery.	ICD 10: C48, C29, C53, C55, C54<br><br>Concurrent use of doxorubicin	10/19/2016	Withdrawn	2/25/2020
11	atezolizumab	Locally advanced or metastatic urothelial carcinoma that progressed during or following platinum-containing chemotherapy or within 12 months of neoadjuvant or adjuvant treatment with platinum-containing chemotherapy.	ICD 10: C67<br>ICD 9: 188<br><br>Prior or later platinum therapy	5/18/2016	Withdrawn	4/13/2021
12	panobinostat	In combination with bortezomib (BTZ) and dexamethasone (DEX) for the treatment of patients with multiple myeloma (MM) who have received at least 2 prior regimens, including BTZ and an immunomodulatory agent.	ICD 10: C900<br>ICD 9: 2030<br><br>Concurrent use of dexamethasone and bortezomib	2/23/2015	Withdrawn	3/24/2022
13	idelalisib	For the treatment of relapsed follicular B-cell Non-Hodgkin Lymphoma (FL) in patients who have received at least 2 prior systemic therapies and relapsed small lymphocytic lymphoma (SLL) in patients who have received at least 2 prior systemic therapies.	ICD 10: C82<br>ICD 9: 202	7/23/2014	Withdrawn	2/18/2022

ID	Drug name	Indication	Algorithm to identify patients	Accelerated approval date	Status	Conversion or withdrawal date
14	vincristine sulfate liposomal	Adults with Philadelphia (PH) chromosome negative (-) acute lymphoblastic leukemia (ALL) in second relapse or greater relapsed or whose disease has progressed following 2 or greater treatment lines of anti-leukemia therapies.	ICD 10: C91.0<br>ICD 9: 204.0	8/9/2012	Withdrawn	5/2/2022
15	ibrutinib	For the treatment of adult patients with mantle cell lymphoma (MCL) who have received at least one prior therapy.	CD 10: C83.1<br>ICD 9: 200.4	2/16/2018	Withdrawn	5/18/2023
16	ibrutinib	For the treatment of patients with mantle cell lymphoma (MCL).	CD 10: C83.1<br>ICD 9: 200.4	11/13/2013	Withdrawn	5/18/2023
17	erdafitinib	Treatment of adult patients with locally advanced or metastatic urothelial carcinoma (mUC), that has: susceptible FGFR3 or FGFR2 genetic alterations, and progressed during or following at least one line of prior platinum-containing chemotherapy, including within 12 months of neoadjuvant or adjuvant platinum-containing chemotherapy.	ICD 10: C67<br>ICD 9: 188	4/12/2019	Verified	1/19/2024
18	pembrolizumab	Treatment of adults and pediatric patients with recurrent locally advanced or metastatic Merkel cell carcinoma (MCC).	ICD 10: C4A	12/19/2018	Verified	10/12/2023
19	pembrolizumab	Treatment of patients with hepatocellular carcinoma (HCC) who have been previously treated with sorafenib.	ICD 10: C22.0	11/9/2018	Verified	1/25/2024
20	pembrolizumab	In combination with chemotherapy for locally recurrent unresectable or metastatic triple-negative breast cancer (TNBC) expressing PD L1 [CPS =10] as determined by an FDA-approved test.	ICD 10: C50<br><br>Concurrent chemotherapy: letrozole, platinum therapy, paclitaxel, or taxane-based	11/13/2020	Verified	7/26/2021
21	selpercatinib	Adult patients with metastatic RET fusion-positive non-small cell lung cancer (NSCLC).	ICD 10: C34	5/8/2020	Verified	09/21/2022
22	capmatinib	Adult patients with metastatic non-small cell lung cancer (mNSCLC) whose tumors have a mutation that leads to MET exon 14 skipping as detected by an FDA-approved test.	ICD 10: C34<br>ICD 9: 162	5/6/2020	Verified	8/10/2022
23	sacituzumab govitecan-hziy	Adults with metastatic triple-negative breast cancer (TNBC) following at least 2 prior therapies for metastatic disease.	ICD 10: C50	4/22/2020	Verified	4/7/2021

ID	Drug name	Indication	Algorithm to identify patients	Accelerated approval date	Status	Conversion or withdrawal date
24	Fam-trastuzumab deruxtecan-nxki	Adult patients with unresectable or metastatic HER2-positive breast cancer who have received two or more prior anti-HER2-based regimens in the metastatic setting.	ICD 10: C50<br>ICD 9: 174	12/20/2019	Verified	5/4/2022
25	enfortumab vedotin-ejfv	Adults with locally advanced or metastatic urothelial cancer who received a PD-1 or PD-L1 inhibitor and a platinum-containing chemotherapy in the neoadjuvant, locally advanced, or metastatic setting.	ICD 10: C67<br>ICD 9: 188<br><br>No concurrent use of pembrolizumab	12/18/2019	Verified	7/9/2021
26	pembrolizumab	In combination with lenvatinib for advanced endometrial carcinoma not MSI-H or dMMR, with progression following systemic therapy and not candidates for curative surgery or radiation.	ICD 10: C54.1<br><br>Concurrent use of lenvatinib	9/17/2019	Verified	7/21/2021
27	lenvatinib	In combination with pembrolizumab for advanced endometrial carcinoma not MSI-H or dMMR, with progression following systemic therapy and not candidates for curative surgery or radiation.	ICD 10: C54.0, C54.1, C54.2, C54.3, C54.8, C54.9, C55<br><br>Concurrent use of pembrolizumab	9/17/2019	Verified	7/21/2021
28	selinexor	In combination with dexamethasone for adults with relapsed/refractory multiple myeloma after at least 4 prior therapies and refractory to at least 2 proteasome inhibitors, 2 immunomodulatory agents, and an anti-CD38 monoclonal antibody.	ICD 10: C90.0<br>ICD 9: 2030<br><br>Concurrent use of dexamethasone<br>No concurrent use of bortezomib	7/3/2019	Verified	12/18/2020
29	polatuzumab vedotin-piiq	In combination with bendamustine and a rituximab product for adult patients with relapsed or refractory diffuse large B-cell lymphoma (DLBCL), not otherwise specified, after at least two prior therapies.	ICD: 10: C83.3	6/10/2019	Verified	4/19/2023

ID	Drug name	Indication	Algorithm to identify patients	Accelerated approval date	Status	Conversion or withdrawal date
			Concurrent use of bendamustine and rituximab			
30	venetoclax	In combination with azacitidine or decitabine or low-dose cytarabine for newly diagnosed acute myeloid leukemia (AML) in adults 75 years or older, or who have comorbidities that preclude use of intensive induction chemotherapy.	ICD 10: C920, C926, C92A	11/21/2018	Verified	16/10/2020
31	lorlatinib	ALK-positive metastatic non-small cell lung cancer (NSCLC) that has progressed on: crizotinib and at least one other ALK inhibitor for metastatic disease; or alectinib as the first ALK inhibitor therapy for metastatic disease; or ceritinib as the first ALK inhibitor therapy for metastatic disease.	ICD 10: C34	11/2/2018	Verified	3/3/2021
32	pembrolizumab	Adult and pediatric patients with refractory primary mediastinal large B-cell lymphoma, or who have relapsed after 2 or more prior lines of therapy.	ICD 10: C85.2	6/13/2018	Verified	10/14/2020
33	pembrolizumab	Treatment of patients with recurrent or metastatic cervical cancer with disease progression on or after chemotherapy whose tumors express PD-L1 (CPS >/=1) as determined by an FDA approved test.	ICD 10: C53<br><br>Chemotherapies excluded include carboplatin and cisplatin	6/12/2018	Verified	10/13/2021
34	pemetrexed disodium	In combination with pembrolizumab and carboplatin for first-line treatment of metastatic non-squamous non-small cell lung cancer (NSCLC).	ICD 10: C34<br>ICD 9: 162<br><br>Concurrent use of pembrolizumab and carboplatin	6/4/2018	Verified	1/30/2019
35	blinatumomab	Treatment of CD19-positive B-cell precursor acute lymphoblastic leukemia (ALL) in first or second complete remission with minimal residual disease (MRD) greater than or equal to 0.1% in adults and children.	ICD 10: C91.01<br>ICD 9: 204.01	3/29/2018	Verified	6/20/2023
36	bosutinib	Adults with newly diagnosed chronic phase Philadelphia chromosome positive chronic myeloid leukemia (CML).	ICD 10: C92.1; C92.2	12/19/2017	Verified	5/14/2021

ID	Drug name	Indication	Algorithm to identify patients	Accelerated approval date	Status	Conversion or withdrawal date
37	pembrolizumab	Treatment of adult and pediatric patients with unresectable or metastatic, microsatellite instability-high (MSI-H) or mismatch repair deficient (dMMR) solid tumors that have progressed following prior treatment and who have no satisfactory alternative treatment options, or metastatic MSI-H or dMMR colorectal cancer that have progressed following treatment with a fluoropyrimidine, oxaliplatin and irinotecan.	* Could not be identified	5/23/2017	Verified	3/28/2023
38	pembrolizumab	Locally advanced or metastatic urothelial carcinoma ineligible for cisplatin-containing chemotherapy.	ICD 10: C67<br><br>No concurrent or later cisplatin therapy	5/18/2017	Verified	8/31/2021
39	pembrolizumab	In combination with pemetrexed and carboplatin for first-line treatment of metastatic non-squamous non-small cell lung cancer (NSCLC).	ICD 10: C34<br><br>Concurrent use of pemetrexed and carboplatin	5/10/2017	Verified	8/20/2018
40	avelumab	Locally advanced or metastatic urothelial carcinoma following disease progression on platinum-containing chemotherapy or disease progression within 12 months of neoadjuvant or adjuvant treatment with platinum-containing chemotherapy.	ICD 10: C67<br>ICD 9: 188	5/9/2017	Verified	6/30/2020
41	brigatinib	Patients with ALK-positive metastatic non-small cell lung cancer (NSCLC) that have progressed or are intolerant to crizotinib.	ICD 10: C34<br>ICD 9: 162	4/28/2017	Verified	5/22/2020
42	avelumab	Treatment of adults and pediatrics patients 12 years and older with metastatic Merkel cell carcinoma (MCC).	ICD 10: C4A<br>ICD 9: 209.31	3/23/2017	Verified	9/6/2023
43	pembrolizumab	Adult and pediatric patients with refractory classical Hodgkin Lymphoma or who have relapsed after 3 or more prior lines of therapy.	ICD 10: C81.9	3/14/2017	Verified	10/14/2020
44	nivolumab	Locally advanced or metastatic urothelial carcinoma that: progressed during or following platinum-containing chemotherapy; progressed within 12 months of neoadjuvant or adjuvant platinum-containing chemotherapy.	ICD 10: C67<br><br>Prior or concurrent use of platinum therapy	2/2/2017	Verified	8/19/2021
45	rucaparib	Deleterious BRCA mutation (germline and/or somatic) associated advanced ovarian cancer treated with 2 or more chemotherapies.	ICD 10: C56	12/19/2016	Verified	4/6/2018

ID	Drug name	Indication	Algorithm to identify patients	Accelerated approval date	Status	Conversion or withdrawal date
46	pembrolizumab	Recurrent or metastatic head and neck squamous cell carcinoma that progressed on or after platinum-containing chemotherapy.	ICD 10: C76.0, C44.42	8/5/2016	Verified	6/10/2019
47	venetoclax	Chronic lymphocytic leukemia with 17P deletion as detected by an FDA-approved test, after at least one prior therapy.	ICD 10: C91.1	4/11/2016	Verified	6/8/2018
48	nivolumab	1) In combination with ipilimumab for unresectable or metastatic melanoma to remove the restriction for treatment of only patients with BRAF wild-type melanoma; 2) As a single agent for BRAF V600 mutation positive unresectable or metastatic melanoma to remove the restriction that such patients should have disease progression following ipilimumab and a BRAF inhibitor.	ICD 10: C43<br>ICD 9: 172	1/23/2016	Verified	3/7/2019
49	alectinib	ALK-positive metastatic non-small cell lung cancer (NSCLC) that progressed on or is intolerant to crizotinib.	ICD 10: C34<br>ICD 9: 162	12/11/2015	Verified	11/6/2017
50	daratumumab	Multiple myeloma after at least 3 prior lines of therapy including a proteasome inhibitor and an immunomodulatory agent or double refractory to a proteasome inhibitor and an immunomodulatory agent.	ICD 10: C900<br>ICD 9: 2030	11/16/2015	Verified	11/21/2016
51	osimertinib	Metastatic EGFR T790M mutation-positive non-small cell lung cancer (NSCLC), as detected by an FDA-approved test, that progressed on or after EGFR TKI therapy.	ICD 10: C34<br>ICD 9: 162	11/13/2015	Verified	3/30/2017
52	pembrolizumab	Metastatic PD-L1 positive non-small cell lung cancer (NSCLC), as determined by an FDA-approved test, with progression on or after platinum-containing chemotherapy.	ICD 10: C34<br>ICD 9: 162	10/2/2015	Verified	10/24/2016
53	nivolumab	In combination with ipilimumab for BRAF V600 wild-type unresectable or metastatic melanoma.	ICD 10: C43<br>ICD 9: 172	9/30/2015	Verified	3/7/2019
54	palbociclib	In combination with letrozole for postmenopausal women with ER-positive, HER2-negative advanced breast cancer as initial endocrine-based therapy for metastatic disease.	ICD 10: C50<br>ICD 9: 174<br><br>Concurrent use of letrozole	2/3/2015	Verified	3/31/2017
55	nivolumab	Unresectable or metastatic melanoma and progression following ipilimumab and, if BRAF V600 mutation positive, a BRAF inhibitor.	ICD 10: C43<br>ICD 9: 172	12/22/2014	Verified	3/7/2019
56	olaparib	Deleterious or suspected deleterious germline BRCA-mutated advanced ovarian cancer after treatment with 3 or more lines of chemotherapy.	ICD 10: C56<br>ICD 9: 183	12/19/2014	Verified	8/17/2017
57	blinatumomab	Philadelphia chromosome negative relapsed or refractory B-cell precursor acute lymphoblastic leukemia.	ICD 10: C91.00<br>C91.02	12/3/2014	Verified	7/11/2017

ID	Drug name	Indication	Algorithm to identify patients	Accelerated approval date	Status	Conversion or withdrawal date
			ICD 9: 204.00; 204.02			
58	pembrolizumab	Unresectable or metastatic melanoma and disease progression following ipilimumab and, if BRAF V600 mutation positive, a BRAF inhibitor.	ICD 10: C43<br>ICD 9: 172	9/4/2014	Verified	12/18/2015
59	ceritinib	ALK-positive metastatic non-small cell lung cancer (NSCLC) that progressed on or is intolerant to crizotinib.	ICD 10: C34<br>ICD 9: 162	4/29/2014	Verified	5/26/2017
60	ibrutinib	Chronic lymphocytic leukemia after at least one prior therapy.	ICD 9: 204.1<br>ICD 10: C91.1	2/12/2014	Verified	7/28/2014
61	dabrafenib	In combination with trametinib for unresectable or metastatic melanoma with BRAF V600E or V600K mutations, as detected by an FDA-approved test.	ICD 10: C43<br>ICD 9: 172<br><br>Concurrent use of trametinib	1/9/2014	Verified	11/20/2015
62	trametinib	In combination with dabrafenib for unresectable or metastatic melanoma with BRAF V600E or V600K mutations, as detected by an FDA-approved test.	ICD 10: C43<br><br>Concurrent use of dabrafenib	1/8/2014	Verified	11/20/2015
63	pertuzumab	In combination with trastuzumab and docetaxel for neoadjuvant treatment of HER2-positive locally advanced inflammatory or early-stage breast cancer (either greater than 2 cm in diameter or node-positive) as part of a complete treatment regimen for early breast cancer.	ICD 10: C50<br>ICD 9: 174<br><br>Concurrent use of trastuzumab and docetaxel	9/30/2013	Verified	12/20/2017
64	pomalidomide	Multiple myeloma after at least 2 prior therapies including lenalidomide and bortezomib and disease progression on or within 60 days of completion of the last therapy.	ICD 10: C900<br>ICD 9: 2030	2/8/2013	Verified	4/23/2015
65	ponatinib	Adults with chronic phase, accelerated phase, or blast phase chronic myeloid leukemia resistant or intolerant to TKI therapy or Philadelphia chromosome positive acute lymphoblastic leukemia resistant or intolerant to prior TKI therapy.	ICD 10: C92.1; C92.2; C91.0<br>ICD 9: 205.1; 204.0	12/14/2012	Verified	11/28/2016
66	omacetaxine mepesuccinate	Adults with chronic or accelerated phase chronic myeloid leukemia with resistance or intolerance to 2 or more TKIs.	ICD 10: C92.1; C92.2<br>ICD 9: 205.1	10/26/2012	Verified	2/10/2014

ID	Drug name	Indication	Algorithm to identify patients	Accelerated approval date	Status	Conversion or withdrawal date
67	carfilzomib	Multiple myeloma after at least 2 prior therapies including bortezomib and an immunomodulatory agent and disease progression on or within 60 days of completion of the last therapy.	ICD 10: C900<br>ICD 9: 2030	7/20/2012	Verified	1/21/2016
68	selinexor	Treatment of adult patients with relapsed or refractory diffuse large B-cell lymphoma (DLBCL), not otherwise specified, including DLBCL arising from follicular lymphoma (FL), after at least 2 lines of systemic therapy.	ICD: 10: C83.3	6/22/2020	Ongoing	/
69	tazemetostat	Treatment of adult patients with relapsed or refractory (R/R) follicular lymphoma (FL) whose tumors are positive for an EZH2 mutation as detected by an FDA-approved test and who have received at least 2 prior systemic therapies. Treatment of adult patients with R/R FL who have no satisfactory alternative treatment options.	ICD 10: C82	6/18/2020	Ongoing	/
70	pembrolizumab	Treatment of adult and pediatric patients with unresectable or metastatic tumor mutational burden high (TMB H) [=10 mutations/megabase (mut/Mb)] solid tumors, as determined by an FDA-approved test, that have progressed following prior treatment and who have no satisfactory alternative treatment options.	* Could not be identified	6/16/2020	Ongoing	/
71	lurbinectedin	Treatment of adult patients with metastatic small cell lung cancer (SCLC) with disease progression on or after prior platinum-based chemotherapy.	ICD 10: C34<br>ICD 9: 162	6/15/2020	Ongoing	/
72	rucaparib	Treatment of adult patients with a deleterious BRCA mutation (germline and/or somatic)-associated metastatic castration-resistant prostate cancer (mCRPC) who have been treated with androgen receptor-directed therapy and a taxane-based chemotherapy.	ICD 10: C61	5/15/2020	Ongoing	/
73	pomalidomide	Treatment of patients with AIDS-related Kaposi's Sarcoma after failure of highly active antiretroviral therapy (HAART).	ICD 10: C46	5/14/2020	Ongoing	/
74	pomalidomide	Treatment of Kaposi's Sarcoma in patients who are HIV-negative.	ICD 10: C46	5/14/2020	Ongoing	/
75	selpercatinib	Adult and pediatric patients 12 years of age and older with advanced or metastatic RET-mutant medullary thyroid cancer (MTC) who require systemic therapy.	ICD 10: C73	5/8/2020	Ongoing	/
76	selpercatinib	Adult and pediatric patients 12 years of age and older with advanced or metastatic RET fusion-positive thyroid cancer who require systemic therapy and who are radioactive iodine-refractory (if radioactive iodine is appropriate).	ICD 10: C73	5/8/2020	Ongoing	/

ID	Drug name	Indication	Algorithm to identify patients	Accelerated approval date	Status	Conversion or withdrawal date
77	pemigatinib	Treatment of adults with previously treated unresectable locally advanced or metastatic cholangiocarcinoma with a fibroblast growth factor receptor 2 (FGFR2) fusion or other rearrangement as detected by an FDA-approved test.	ICD 10: C22.1	4/17/2020	Ongoing	/
78	ipilimumab	In combination with nivolumab, for the treatment of patients with hepatocellular carcinoma (HCC) who have been previously treated with sorafenib.	ICD 10: C22.0<br>ICD 9: 155.0<br><br>Concurrent use of nivolumab	3/10/2020	Ongoing	/
79	nivolumab	In combination with ipilimumab, for the treatment of patients with hepatocellular carcinoma (HCC) who have been previously treated with sorafenib.	ICD 10: C22.0<br><br>Concurrent use of ipilimumab	3/10/2020	Ongoing	/
80	tazemetostat	Treatment of adults and pediatric patients aged 16 years and older with metastatic or locally advanced epithelioid sarcoma not eligible for complete resection.	ICD 10: C49.9	1/23/2020	Ongoing	/
81	zanubrutinib	Treatment of adult patients with mantle cell lymphoma (MCL) who have received at least one prior therapy.	ICD 10: C83.1<br>ICD 9: 200.4	11/14/2019	Ongoing	/
82	entrectinib	Treatment of adult and pediatric patients 12 years of age and older with solid tumors that have a neurotrophic tyrosine receptor kinase (NTRK) gene fusion without a known acquired resistance mutation, are metastatic or where surgical resection is likely to result in severe morbidity, and have progressed following treatment or have no satisfactory alternative therapy.	ICD 10: C0, C1, C2, C3, C4, C5, C6, C7, C80<br><br>Excluding lung cancer (C34)	8/15/2019	Ongoing	/
83	larotrectinib	Treatment of adult and pediatric patients with solid tumors that have a neurotrophic receptor tyrosine kinase (NTRK) gene fusion without a known acquired resistance mutation; are metastatic or where surgical resection is likely to result in severe morbidity; and have no satisfactory alternative treatments or that have progressed following treatment.	ICD 10: C0, C1, C2, C3, C4, C5, C6, C7, C80	11/26/2018	Ongoing	/
84	nivolumab	In combination with ipilimumab, is indicated for the treatment of adults and pediatric patients 12 years and older with microsatellite instability-high (MSI-H) or DNA mismatch repair deficient (dMMR), metastatic colorectal cancer that has progressed following treatment with a fluoropyrimidine, oxaliplatin, and irinotecan.	ICD 10: C18<br><br>Concurrent use of ipilimumab	7/10/2018	Ongoing	/

ID	Drug name	Indication	Algorithm to identify patients	Accelerated approval date	Status	Conversion or withdrawal date
85	ipilimumab	In combination with nivolumab, is indicated for the treatment of adults and pediatric patients 12 years and older with microsatellite instability-high (MSI-H) or DNA mismatch repair deficient (dMMR), metastatic colorectal cancer that has progressed following treatment with a fluoropyrimidine, oxaliplatin, and irinotecan.	ICD 10: C18<br>ICD 9: 153<br><br>Concurrent use of nivolumab	7/10/2018	Ongoing	/
86	acalabrutinib	Treatment of adult patients with mantle cell lymphoma (MCL) who have received at least one prior therapy.	ICD 10: C83.1<br>ICD 9: 200.4	10/31/2017	Ongoing	/
87	nivolumab	For the treatment of adult and pediatric patients 12 years and older with microsatellite instability-high (MSI-H) or mismatch repair deficient (dMMR) metastatic colorectal cancer (CRC) that has progressed following treatment with a fluoropyrimidine, oxaliplatin, and irinotecan.	ICD 10: C18<br><br>No concurrent use of ipilimumab	7/31/2017	Ongoing	/
88	nivolumab	Treatment of adult patients with classical Hodgkin lymphoma that has relapsed or progressed after: autologous hematopoietic stem cell transplantation (HSCT) and brentuximab vedotin, or 3 or more lines of systemic therapy that includes autologous HSCT.	ICD 10: C81.9	4/25/2017	Ongoing	/
89	nivolumab	For the treatment of classical Hodgkin Lymphoma that has relapsed or progressed after autologous hematopoietic stem cell transplantation (HSCT) and post-transplantation brentuximab vedotin.	ICD 10: C81.9	5/17/2016	Ongoing	/
90	belinostat	Treatment of relapsed or refractory peripheral T-Cell Lymphoma (PTCL).	ICD 10: C84.4<br>ICD 9: 202.7	7/3/2014	Ongoing	/

Indications are ordered by accelerated approval status (withdrawn, verified, ongoing) and approval date. Within each status category, indications are listed from most to least recent.

Appendix table 2. Characteristics of confirmatory trials.

ID	Drug name	Indication	Confirmatory trial identifier(s)	Design	Experimental treatment	Confirmatory control arm	Primary endpoint(s)
1	pembrolizumab	Metastatic small cell lung cancer (SCLC) with disease progression on or after platinum-based chemotherapy and at least one other prior line of therapy.	NCT03066778 KEYNOTE-604	RCT	pembrolizumab + etoposide + platinum therapy (carboplatin or cisplatin)	placebo + etoposide + platinum therapy (carboplatin or cisplatin)	PFS, OS
2	atezolizumab	In combination with paclitaxel protein-bound for unresectable locally advanced or metastatic triple-negative breast cancer whose tumors express PD-L1 (PD-L1 stained tumor-infiltrating immune cells of any intensity covering = 1% of the tumor area), as determined by an FDA-approved test.	NCT03125902 MO39196 (IMpassion131)	RCT	atezolizumab + paclitaxel	placebo + paclitaxel	PFS
3	duvelisib	Treatment of adult patients with relapsed or refractory follicular lymphoma (FL) after at least 2 prior systemic therapies.	NCT02204982 DYNAMO + R	RCT	duvelisib + rituximab	placebo + rituximab	PFS
4	nivolumab	Metastatic small cell lung cancer (SCLC) with progression after platinum-based chemotherapy and at least one other line of therapy.	NCT02538666<br>NCT02481830<br>CheckMate-451<br>CheckMate-331	RCT<br>RCT	CheckMate-451: nivolumab monotherapy/ nivolumab + ipilimumab<br><br>CheckMate-331: nivolumab	CheckMate-451: placebo<br><br>CheckMate-331: chemotherapy (topotecan/ amrubicin)	ORR<br>OS
5	pembrolizumab	For patients with recurrent or locally advanced or metastatic gastric or gastroesophageal junction (GEJ) adenocarcinoma whose tumors express PD-L1 [CPS ≥1] as determined by an FDA-approved test, with disease progression on/after two or more prior lines of therapy including fluoropyrimidine and platinum containing chemotherapy and if appropriate, HER2/NEU targeted therapy.	NCT02370498<br>NCT02494583<br>KEYNOTE-061 (second line)<br>KEYNOTE-062 (front line)	RCT<br>RCT	KEYNOTE-061: pembrolizumab<br><br>KEYNOTE-062: pembrolizumab monotherapy/ pembrolizumab + chemo (cisplatin +	KEYNOTE-061: paclitaxel<br><br>KEYNOTE-062: placebo + chemo (cisplatin + 5-FU + capecitabine)	PFS, OS

ID	Drug name	Indication	Confirmatory trial identifier(s)	Design	Experimental treatment	Confirmatory control arm	Primary endpoint(s)
					5-FU + capecitabine)		
6	nivolumab	Hepatocellular carcinoma previously treated with sorafenib.	NCT02576509 CheckMate-459	RCT	nivolumab	sorafenib	OS
7	durvalumab	Locally advanced or metastatic urothelial carcinoma that progressed during or following platinum-containing chemotherapy or within 12 months of neoadjuvant or adjuvant treatment with platinum-containing chemotherapy.	NCT02516241 DANUBE	RCT	durvalumab + tremelimumab/ durvalumab monotherapy	SoC chemotherapy (cisplatin + gemcitabine or carboplatin + gemcitabine)	OS
8	atezolizumab	Patients with locally advanced or metastatic urothelial carcinoma (mUC) who are not eligible for cisplatin-containing chemotherapy and whose tumors express PD-L1 as determined by an FDA approved test or who are not eligible for any platinum-containing chemotherapy regardless of PD-L1 status.	NCT02807636 IMvigor130	RCT	atezolizumab + gemcitabine + carboplatin/ atezolizumab monotherapy	placebo + gemcitabine + carboplatin/ cisplatin	PFS, OS
9	ibrutinib	Adult patients with marginal zone lymphoma (MZL) who require systemic therapy and have received at least one prior anti-CD20-based therapy.	NCT01974440 SELENE	RCT	chemotherapy (bendamustine + rituximab [BR] or rituximab, cyclophosphamide, doxorubicin, vincristine and prednisone [R-CHOP]) + ibrutinib	chemotherapy (bendamustine + rituximab [BR] or rituximab, cyclophosphamide, doxorubicin, vinscristine and prednisone [R-CHOP]) + placebo	PFS
10	olaratumab	In combination with doxorubicin for adults with soft tissue sarcoma with a histologic subtype for which an anthracycline-containing regimen is appropriate and which is not amenable to curative treatment with radiotherapy or surgery.	NCT02451943 ANNOUNCE	RCT	doxorubicin + olaratumab	placebo + doxorubicin	OS
11	atezolizumab	Locally advanced or metastatic urothelial carcinoma that progressed during or following platinum-containing chemotherapy or within 12	NCT02302807 GO29294	RCT	atezolizumab	chemotherapy (vinflunine, paclitaxel or docetaxel)	OS

ID	Drug name	Indication	Confirmatory trial identifier(s)	Design	Experimental treatment	Confirmatory control arm	Primary endpoint(s)
		months of neoadjuvant or adjuvant treatment with platinum-containing chemotherapy.					
12	panobinostat	In combination with bortezomib (BTZ) and dexamethasone (DEX) for the treatment of patients with multiple myeloma (MM) who have received at least 2 prior regimens, including BTZ and an immunomodulatory agent.	NCT01023308<br>PANORAMA-1	RCT	panobinostat + bortezomib + dexamethasone	placebo + bortezomib + dexamethasone	PFS
13	idelalisib	For the treatment of relapsed follicular B-cell Non-Hodgkin Lymphoma (FL) in patients who have received at least 2 prior systemic therapies and relapsed small lymphocytic lymphoma (SLL) in patients who have received at least 2 prior systemic therapies.	NCT01282424<br>DELTA	SAT	idelalisib	NA	ORR
14	vincristine sulfate liposomal	Adults with Philadelphia (PH) chromosome negative (-) acute lymphoblastic leukemia (ALL) in second relapse or greater relapsed or whose disease has progressed following 2 or greater treatment lines of anti-leukemia therapies.	NCT01439347<br>TTX404	RCT	vincristine sulfate liposomes injection	vincristine sulfate injection	OS
15	ibrutinib	For the treatment of adult patients with mantle cell lymphoma (MCL) who have received at least one prior therapy.	NCT01776840<br>PCI-32765MCL3002 (SHINE)	RCT	ibrutinib + BR (bendamustine, rituximab)	placebo + BR (bendamustine, rituximab)	PFS
16	ibrutinib	For the treatment of patients with mantle cell lymphoma (MCL).	NCT01236391<br>NCT01776840<br>PCYC-1104-CA<br>PCI-32765MCL3002 (SHINE)	SAT<br>RCT	 ibrutinib + BR (bendamustine, rituximab)	 placebo + BR (bendamustine, rituximab)	ORR<br>PFS
17	erdafitinib	Treatment of adult patients with locally advanced or metastatic urothelial carcinoma (mUC), that has: susceptible FGFR3 or FGFR2 genetic alterations, and progressed during or following at least one line of prior platinum-containing chemotherapy, including within 12	NCT03390504<br>Study BLC3001	RCT	erdafitnib	vinflunine or docetaxel	OS

ID	Drug name	Indication	Confirmatory trial identifier(s)	Design	Experimental treatment	Confirmatory control arm	Primary endpoint(s)
		months of neoadjuvant or adjuvant platinum-containing chemotherapy.					
18	pembrolizumab	Treatment of adults and pediatric patients with recurrent locally advanced or metastatic Merkel cell carcinoma (MCC).	NCT02267603<br>NCT03783078<br>KEYNOTE-017<br>KEYNOTE-913	SAT<br>SAT	pembrolizumab	NA	ORR<br>ORR
19	pembrolizumab	Treatment of patients with hepatocellular carcinoma (HCC) who have been previously treated with sorafenib.	NCT03062358<br>KEYNOTE-394	RCT	pembrolizumab + best supportive care	placebo + best supportive care	OS
20	pembrolizumab	In combination with chemotherapy for locally recurrent unresectable or metastatic triple-negative breast cancer (TNBC) expressing PD L1 [CPS =10] as determined by an FDA-approved test.	NCT02819518<br>KEYNOTE-355	RCT	pembrolizumab + chemo (nab-paclitaxel, paclitaxel or gemcitabine/ carboplatin)	placebo + chemo (nab-paclitaxel, paclitaxel or gemcitabine/ carboplatin)	PFS, OS
21	selpercatinib	Adult patients with metastatic RET fusion-positive non-small cell lung cancer (NSCLC).	NCT03157128<br>LIBRETTO-001	SAT	selpercatinib	pemetrexed and chemo (carboplatin or cisplatin) with or without pembrolizumab	ORR
22	capmatinib	Adult patients with metastatic non-small cell lung cancer (mNSCLC) whose tumors have a mutation that leads to MET exon 14 skipping as detected by an FDA-approved test.	NCT02414139<br>GEOMETRY mono-1	SAT	capmatinib	docetaxel	ORR
23	sacituzumab govitecan-hziy	Adults with metastatic triple-negative breast cancer (TNBC) following at least 2 prior therapies for metastatic disease.	NCT02574455<br>ASCENT	RCT	sacituzumab govitecan	treatment of physician's choice (eribulin, capecitabine, gemcitabine, or vinorelbine)	PFS
24	fam-trastuzumab deruxtecan-nxki	Adult patients with unresectable or metastatic HER2-positive breast cancer who have received two or more prior anti-HER2-based regimens in the metastatic setting.	NCT03529110<br>DESTINY-Breast03	RCT	trastuzumab deruxtecan (T-DXd)	ado-trastuzumab emtansine (T-DM1)	PFS

ID	Drug name	Indication	Confirmatory trial identifier(s)	Design	Experimental treatment	Confirmatory control arm	Primary endpoint(s)
25	enfortumab vedotin-ejfv	Adults with locally advanced or metastatic urothelial cancer who received a PD-1 or PD-L1 inhibitor and a platinum-containing chemotherapy in the neoadjuvant, locally advanced, or metastatic setting.	NCT03474107<br>EV-301	RCT	enfortumab vedotin	chemotherapy (docetaxel, vinflunine or paclitaxel)	OS
26	pembrolizumab	In combination with lenvatinib for advanced endometrial carcinoma not MSI-H or dMMR, with progression following systemic therapy and not candidates for curative surgery or radiation.	NCT03517449<br>KEYNOTE-775	RCT	lenvatinib + pembrolizumab	treatment of physician's choice (doxorubicin or paclitaxel)	PFS, OS
27	lenvatinib	In combination with pembrolizumab for advanced endometrial carcinoma not MSI-H or dMMR, with progression following systemic therapy and not candidates for curative surgery or radiation.	NCT03517449<br>KEYNOTE-775	RCT	lenvatinib + pembrolizumab	treatment of physician's choice (doxorubicin or paclitaxel)	PFS, OS
28	selinexor	In combination with dexamethasone for adults with relapsed/refractory multiple myeloma after at least 4 prior therapies and refractory to at least 2 proteasome inhibitors, 2 immunomodulatory agents, and an anti-CD38 monoclonal antibody.	NCT03110562<br>KCP-330-023 (BOSTON)	RCT	SVd (selinexor + bortezomib + dexamethasone)	Vd (bortezomib + dexamethasone)	PFS
29	polatuzumab vedotin- piiq	In combination with bendamustine and a rituximab product for adult patients with relapsed or refractory diffuse large B-cell lymphoma (DLBCL), not otherwise specified, after at least two prior therapies.	NCT03274492<br>NCT04182204<br>GO39942 (POLARIX)<br>MO40598	RCT<br>RCT	GO39942: pola-R-CHP (polatuzumab vedotin + rituximab + cyclophosphamide + doxorubicin + prednisone) + placebo for vincristine<br><br>MO40598: pola-R-GemOx (polatuzumab vedotin + rituximab	GO39942: R-CHOP (rituximab + cyclophosphamide + doxorubicin + vincristine + prednisone) + placebo for polatuzumab vedotin<br><br>MO40598: R-GemOx (rituximab + gemcitabine + oxaliplatin)	PFS<br>OS

ID	Drug name	Indication	Confirmatory trial identifier(s)	Design	Experimental treatment	Confirmatory control arm	Primary endpoint(s)
					+ gemcitabine + oxaliplatin)		
30	venetoclax	In combination with azacitidine or decitabine or low-dose cytarabine for newly diagnosed acute myeloid leukemia (AML) in adults 75 years or older, or who have comorbidities that preclude use of intensive induction chemotherapy.	NCT02993523<br>NCT03069352<br>VIALE-A (M15-656)<br>VIALE-C (M16-043)	RCT<br>RCT	VIALE-A: venetoclax + azacitidine<br><br>VIALE-C: venetoclax + cytarabine	VIALE-A: placebo + azacitidine<br><br>VIALE-C: placebo + cytarabine	OS, CR (complete response)<br>OS
31	lorlatinib	ALK-positive metastatic non-small cell lung cancer (NSCLC) that has progressed on: crizotinib and at least one other ALK inhibitor for metastatic disease; or alectinib as the first ALK inhibitor therapy for metastatic disease; or ceritinib as the first ALK inhibitor therapy for metastatic disease.	NCT03052608<br>CROWN (B7461006)	RCT	lorlatinib	crizotinib	PFS
32	pembrolizumab	Adult and pediatric patients with refractory primary mediastinal large B-cell lymphoma, or who have relapsed after 2 or more prior lines of therapy.	NCT02684292<br>KEYNOTE-204	RCT	pembrolizumab	brentuximab vedotin	PFS, OS
33	pembrolizumab	Treatment of patients with recurrent or metastatic cervical cancer with disease progression on or after chemotherapy whose tumors express PD-L1 (CPS >/=1) as determined by an FDA approved test.	NCT03635567<br>KEYNOTE-826	RCT	pembrolizumab + chemotherapy (paclitaxel + cisplatin/ carboplatin) with or without bevacizumab	placebo + chemotherapy (paclitaxel + cisplatin/ carboplatin) with or without bevacizumab	PFS, OS
34	pemetrexed disodium	In combination with pembrolizumab and carboplatin for first-line treatment of metastatic non-squamous non-small cell lung cancer (NSCLC).	NCT02578680<br>KEYNOTE-189	RCT	pembrolizumab + pemetrexed + chemo (cisplatin or carboplatin)	placebo + pemetrexed + chemo (cisplatin or carboplatin)	PFS, OS
35	blinatumomab	Treatment of CD19-positive B-cell precursor acute lymphoblastic leukemia (ALL) in first or second complete remission with minimal	NCT02003222<br>AALL1331	RCT	blinatumomab + cyclophosphamide + cytarabine + daunorubicin +	cyclophosphamide + cytarabine + daunorubicin + dexamethasone +	OS

ID	Drug name	Indication	Confirmatory trial identifier(s)	Design	Experimental treatment	Confirmatory control arm	Primary endpoint(s)
		residual disease (MRD) greater than or equal to 0.1% in adults and children.			dexamethasone + etoposide + leucovorin calcium + mercaptopurine + methotrexate + prednisone + vincristine	etoposide + leucovorin calcium + mercaptopurine + methotrexate + prednisone + vincristine	
36	bosutinib	Adults with newly diagnosed chronic phase Philadelphia chromosome positive chronic myeloid leukemia (CML).	NCT02130557<br>BFORE	RCT	bosutinib	imatinib	MMR (major molecular response)
37	pembrolizumab	Treatment of adult and pediatric patients with unresectable or metastatic, microsatellite instability-high (MSI-H) or mismatch repair deficient (dMMR) solid tumors that have progressed following prior treatment and who have no satisfactory alternative treatment options, or metastatic MSI-H or dMMR colorectal cancer that have progressed following treatment with a fluoropyrimidine, oxaliplatin and irinotecan.	NCT02460198<br>NCT02628067<br>NCT02332668<br>NCT02563002<br>KEYNOTE-164<br>KEYNOTE-158<br>KEYNOTE-051<br>KEYNOTE-177	SAT<br>RCT	pembrolizumab	standard of care (mFOLFOX6 or mFOLFOX6 + bevacizumab/cetuximab or FOLFIRI or FOLFIRI +bevacizumab/cetuximab)	ORR<br>ORR<br>ORR<br>PFS, OS
38	pembrolizumab	Locally advanced or metastatic urothelial carcinoma ineligible for cisplatin-containing chemotherapy.	NCT02853305<br>KEYNOTE-361	RCT	pembrolizumab + chemo (cisplatin or carboplatin + gemcitabine)/pembrolizumab	chemo (cisplatin or carboplatin + gemcitabine)	PFS, OS
39	pembrolizumab	In combination with pemetrexed and carboplatin for first-line treatment of metastatic non-squamous non-small cell lung cancer (NSCLC).	NCT02578680<br>KEYNOTE-189	RCT	pembrolizumab + pemetrexed + chemo (cisplatin or carboplatin)	placebo + pemetrexed + chemo (cisplatin or carboplatin)	PFS, OS
40	avelumab	Locally advanced or metastatic urothelial carcinoma following disease progression on platinum-containing chemotherapy or disease progression within 12 months of neoadjuvant or adjuvant treatment with platinum-containing chemotherapy.	NCT02603432<br>JAVELIN Bladder 100	RCT	avelumab + best supportive care	best supportive care	OS

ID	Drug name	Indication	Confirmatory trial identifier(s)	Design	Experimental treatment	Confirmatory control arm	Primary endpoint(s)
41	brigatinib	Patients with ALK-positive metastatic non-small cell lung cancer (NSCLC) that have progressed or are intolerant to crizotinib.	NCT02737501 ALTA 1L	RCT	brigatinib	crizotinib	PFS
42	avelumab	Treatment of adults and pediatrics patients 12 years and older with metastatic Merkel cell carcinoma (MCC).	NCT02155647 JAVELIN Merkel 200	SAT	avelumab	NA	BOR (best overall response), DRR (durable response rate)
43	pembrolizumab	Adult and pediatric patients with refractory classical Hodgkin Lymphoma or who have relapsed after 3 or more prior lines of therapy.	NCT02684292 KEYNOTE-204	RCT	pembrolizumab	brentuximab vedotin	PFS, OS
44	nivolumab	Locally advanced or metastatic urothelial carcinoma that: progressed during or following platinum-containing chemotherapy; progressed within 12 months of neoadjuvant or adjuvant platinum-containing chemotherapy.	NCT02632409 CHECKMATE-274	RCT	nivolumab	placebo	DFS (disease-free survival)
45	rucaparib	Deleterious BRCA mutation (germline and/or somatic) associated advanced ovarian cancer treated with 2 or more chemotherapies.	NCT01968213 ARIEL3	RCT	rucaparib	placebo	PFS
46	pembrolizumab	Recurrent or metastatic head and neck squamous cell carcinoma that progressed on or after platinum-containing chemotherapy.	NCT02358031 KEYNOTE-048	RCT	pembrolizumab/pembrolizumab + chemo (cisplatin or carboplatin; plus 5-FU)	cetuximab + chemo (cisplatin or carboplatin; plus 5-FU)	PFS, OS
47	venetoclax	Chronic lymphocytic leukemia with 17P deletion as detected by an FDA-approved test, after at least one prior therapy.	NCT02005471 MURANO (GO28667)	RCT	venetoclax + rituximab	bendamustine + rituximab	PFS
48	nivolumab	1) In combination with ipilimumab for unresectable or metastatic melanoma to remove the restriction for treatment of only patients with BRAF wild-type melanoma; 2) As a single agent for BRAF V600 mutation positive unresectable or metastatic melanoma to remove the	NCT01721772 CHECKMATE-066	RCT	nivolumab	dacarbazine	OS

ID	Drug name	Indication	Confirmatory trial identifier(s)	Design	Experimental treatment	Confirmatory control arm	Primary endpoint(s)
		restriction that such patients should have disease progression following ipilimumab and a BRAF inhibitor.					
49	alectinib	ALK-positive metastatic NSCLC that progressed on or is intolerant to crizotinib.	NCT02075840 ALEX	RCT	alectinib	crizotinib	PFS
50	daratumumab	Multiple myeloma after at least 3 prior lines of therapy including a proteasome inhibitor and an immunomodulatory agent or double refractory to a proteasome inhibitor and an immunomodulatory agent.	NCT02076009<br>NCT02136134<br>MMY3003 (POLLUX)<br>MMY3004 (CASTOR)	RCT<br>RCT	POLLUX: daratumumab + lenalidomide + dexamethasone<br><br>CASTOR: daratumumab + bortezomib + dexamethasone	POLLUX: lenalidomide + dexamethasone<br><br>CASTOR: bortezomib + dexamethasone	PFS<br>PFS
51	osimertinib	Metastatic EGFR T790M mutation-positive non-small cell lung cancer (NSCLC), as detected by an FDA-approved test, that progressed on or after EGFR TKI therapy.	NCT02151981 AURA3	RCT	osimertinib	Chemotherapy (pemetrexed + carboplatin/ cisplatin)	PFS
52	pembrolizumab	Metastatic PD-L1 positive non-small cell lung cancer (NSCLC), as determined by an FDA-approved test, with progression on or after platinum-containing chemotherapy.	NCT01905657 KEYNOTE-010	RCT	pembrolizumab	docetaxel	OS, PFS
53	nivolumab	In combination with ipilimumab for BRAF V600 wild-type unresectable or metastatic melanoma.	NCT01844505 CHECKMATE-067	RCT	nivolumab/ nivolumab + ipilimumab	ipilimumab	PFS, OS
54	palbociclib	In combination with letrozole for postmenopausal women with ER-positive, HER2-negative advanced breast cancer as initial endocrine-based therapy for metastatic disease.	NCT01740427 PALOMA-2	RCT	palbociclib + letrozole	placebo + letrozole	PFS
55	nivolumab	Unresectable or metastatic melanoma and progression following ipilimumab and, if BRAF V600 mutation positive, a BRAF inhibitor.	NCT01721746 CHECKMATE-037	RCT	nivolumab	dacarbazine or carboplatin + paclitaxel	ORR, OS

ID	Drug name	Indication	Confirmatory trial identifier(s)	Design	Experimental treatment	Confirmatory control arm	Primary endpoint(s)
56	olaparib	Deleterious or suspected deleterious germline BRCA-mutated advanced ovarian cancer after treatment with 3 or more lines of chemotherapy.	NCT01874353 D0818C00002 (SOLO-2)	RCT	olaparib	placebo	PFS
57	blinatumomab	Philadelphia chromosome negative relapsed or refractory B-cell precursor acute lymphoblastic leukemia.	NCT02013167 TOWER	RCT	blinatumomab	standard of care chemotherapy: 1. fludarabine, cytarabine arabinoside, and granulocyte colony-stimulating factor ± anthracycline-based regimen; 2. high-dose cytarabine arabinoside - based regimen ± anthracycline and/or in combination with other drugs; 3. high-dose methotrexate-based regimen; 4. clofarabine or clofarabine-based regimens	OS
58	pembrolizumab	Unresectable or metastatic melanoma and disease progression following ipilimumab and, if BRAF V600 mutation positive, a BRAF inhibitor.	NCT01866319 NCT01704287 KEYNOTE-006 (Ipilimumab-Naive Melanoma) KEYNOTE-002 (Ipilimumab-	RCT RCT	KEYNOTE-006: pembrolizumab<br><br>KEYNOTE-002: pembrolizumab	KEYNOTE-006: ipilimumab<br><br>KEYNOTE-002: chemo (carboplatin + paclitaxel/ paclitaxel/ dacarbazine/	PFS, OS PFS, OS

ID	Drug name	Indication	Confirmatory trial identifier(s)	Design	Experimental treatment	Confirmatory control arm	Primary endpoint(s)
			Refractory Melanoma)			temozolomide)	
59	ceritinib	ALK-positive metastatic non-small cell lung cancer (NSCLC) that progressed on or is intolerant to crizotinib.	NCT01828099 ASCEND-4	RCT	ceritinib	chemo (pemetrexed + cisplatin or carboplatin)	PFS
60	ibrutinib	Chronic lymphocytic leukemia after at least one prior therapy.	NCT01578707 PCYC-1112-CA (RESONATE)	RCT	ibrutinib	ofatumumab	PFS
61	dabrafenib	In combination with trametinib for unresectable or metastatic melanoma with BRAF V600E or V600K mutations, as detected by an FDA-approved test.	NCT01584648 MEK115306 (COMBI-d)	RCT	dabrafenib + trametinib	dabrafenib + placebo	PFS
62	trametinib	In combination with dabrafenib for unresectable or metastatic melanoma with BRAF V600E or V600K mutations, as detected by an FDA-approved test.	NCT01584648 MEK115306 (COMBI-d)	RCT	dabrafenib + trametinib	dabrafenib + placebo	PFS
63	pertuzumab	In combination with trastuzumab and docetaxel for neoadjuvant treatment of HER2-positive locally advanced inflammatory or early-stage breast cancer (either greater than 2 cm in diameter or node-positive) as part of a complete treatment regimen for early breast cancer.	NCT01358877 BO25126 (APHINITY)	RCT	pertuzumab + trastuzumab + chemotherapy (5-fluorouracil, carboplatin, cyclophosphamide, docetaxel, doxorubicin, epirubicin, paclitaxel)	placebo + trastuzumab + chemotherapy (5-fluorouracil, carboplatin, cyclophosphamide, docetaxel, doxorubicin, epirubicin, paclitaxel)	IDFS (invasive disease-free survival)
64	pomalidomide	Multiple myeloma after at least 2 prior therapies including lenalidomide and bortezomib and disease progression on or within 60 days of completion of the last therapy.	NCT01311687 NIMBUS	RCT	pomalidomide + dexamethasone (low-dose)	dexamethasone (high-dose)	PFS
65	ponatinib	Adults with chronic phase, accelerated phase, or blast phase chronic myeloid leukemia resistant or intolerant to TKI therapy or Philadelphia chromosome positive acute lymphoblastic	NCT01207440 PACE (AP24534-10-201)	SAT	ponatinib	NA	MCyR (major cytogenetic response),

ID	Drug name	Indication	Confirmatory trial identifier(s)	Design	Experimental treatment	Confirmatory control arm	Primary endpoint(s)
		leukemia resistant or intolerant to prior TKI therapy.					MaHR (major hematologic response)
66	omacetaxine mepesuccinate	Adults with chronic or accelerated phase chronic myeloid leukemia with resistance or intolerance to 2 or more TKIs.	NCT00375219 NCT00462943 CGX-635-CML-202 CGX-635-CML-203	SAT	omacetaxine mepesuccinate	NA	CHR (complete hematologic response)
67	carfilzomib	Multiple myeloma after at least 2 prior therapies including bortezomib and an immunomodulatory agent and disease progression on or within 60 days of completion of the last therapy.	NCT01080391 PX-171-009 (ASPIRE)	RCT	carfilzomib + lenalidomide + dexamethasone	lenalidomide + dexamethasone	PFS
68	selinexor	Treatment of adult patients with relapsed or refractory diffuse large B-cell lymphoma (DLBCL), not otherwise specified, including DLBCL arising from follicular lymphoma (FL), after at least 2 lines of systemic therapy.	NCT04442022 XPORT-DLBCL-030	RCT	selinexor + R-GDP (rituximab + gemcitabine + dexamethasone + cisplatin)	placebo + R-GDP (rituximab + gemcitabine + dexamethasone + cisplatin)	ORR, PFS
69	tazemetostat	Treatment of adult patients with relapsed or refractory (R/R) follicular lymphoma (FL) whose tumors are positive for an EZH2 mutation as detected by an FDA-approved test and who have received at least 2 prior systemic therapies. Treatment of adult patients with R/R FL who have no satisfactory alternative treatment options.	NCT04224493 SYMPHONY-1	RCT	tazemetostat + R2 (rituximab + lenalidomide)	placebo + R2 (rituximab + lenalidomide)	PFS
70	pembrolizumab	Treatment of adult and pediatric patients with unresectable or metastatic tumor mutational burden high (TMB H) [=10 mutations/megabase (mut/Mb)] solid tumors, as determined by an FDA-approved test, that have progressed following prior treatment and who have no satisfactory alternative treatment options.	NCT02628067 KEYNOTE-158	SAT	pembrolizumab	NA	ORR

ID	Drug name	Indication	Confirmatory trial identifier(s)	Design	Experimental treatment	Confirmatory control arm	Primary endpoint(s)
71	lurbinectedin	Treatment of adult patients with metastatic small cell lung cancer (SCLC) with disease progression on or after prior platinum-based chemotherapy.	NCT02566993 ATLANTIS	RCT	lurbinectedin + doxorubicin	CAV (cyclophosphamide + doxorubicin + vincristine) or topotecan	OS
72	rucaparib	Treatment of adult patients with a deleterious BRCA mutation (germline and/or somatic)-associated metastatic castration-resistant prostate cancer (mCRPC) who have been treated with androgen receptor-directed therapy and a taxane-based chemotherapy.	NCT02975934 TRITON3	RCT	rucaparib	abiraterone acetate/ enzalutamide/ docetaxel	PFS
73	pomalidomide	Treatment of patients with AIDS-related Kaposi's Sarcoma after failure of highly active antiretroviral therapy (HAART).	NCT04577755 NCI-2020-07565	SAT	pomalidomide	NA	DoR (duration of response)
74	pomalidomide	Treatment of Kaposi's Sarcoma in patients who are HIV-negative.	NCT04577755 NCI-2020-07565	SAT	pomalidomide	NA	DoR (duration of response)
75	selpercatinib	Adult and pediatric patients 12 years of age and older with advanced or metastatic RET-mutant medullary thyroid cancer (MTC) who require systemic therapy.	NCT04211337 LIBRETTO-531	RCT	selpercatinib	cabozantinib/ vandetanib	PFS
76	selpercatinib	Adult and pediatric patients 12 years of age and older with advanced or metastatic RET fusion-positive thyroid cancer who require systemic therapy and who are radioactive iodine-refractory (if radioactive iodine is appropriate).	NCT03157128 LIBRETTO-001	SAT	selpercatinib	NA	ORR
77	pemigatinib	Treatment of adults with previously treated unresectable locally advanced or metastatic cholangiocarcinoma with a fibroblast growth factor receptor 2 (FGFR2) fusion or other rearrangement as detected by an FDA-approved test.	NCT03656536 FIGHT-302	RCT	pemigatinib	gemcitabine + cisplatin	PFS
78	ipilimumab	In combination with nivolumab, for the treatment of patients with hepatocellular carcinoma (HCC)	NCT04039607 CheckMate 9DW	RCT	nivolumab + ipilimumab	sorafenib/ levatinib	OS

ID	Drug name	Indication	Confirmatory trial identifier(s)	Design	Experimental treatment	Confirmatory control arm	Primary endpoint(s)
		who have been previously treated with sorafenib.					
79	nivolumab	In combination with ipilimumab, for the treatment of patients with hepatocellular carcinoma (HCC) who have been previously treated with sorafenib.	NCT04039607 CheckMate 9DW	RCT	nivolumab + ipilimumab	sorafenib/ levatinib	OS
80	tazemetostat	Treatment of adults and pediatric patients aged 16 years and older with metastatic or locally advanced epithelioid sarcoma not eligible for complete resection.	NCT04204941 EZH-301	RCT	tazemetostat + doxorubicin	placebo + doxorubicin	PFS
81	zanubrutinib	Treatment of adult patients with mantle cell lymphoma (MCL) who have received at least one prior therapy.	NCT04002297 BGB-3111-306	RCT	zanubrutinib + rituximab	bendamustine + rituximab	PFS
82	entrectinib	Treatment of adult and pediatric patients 12 years of age and older with solid tumors that have a neurotrophic tyrosine receptor kinase (NTRK) gene fusion without a known acquired resistance mutation, are metastatic or where surgical resection is likely to result in severe morbidity, and have progressed following treatment or have no satisfactory alternative therapy.	EudraCT 2012-000148-88<br>NCT02097810<br>NCT02568267<br>ALKA<br>STARTRK-1<br>STARTRK-2	SAT	entrectinib	NA	ORR<br>ORR
83	larotrectinib	Treatment of adult and pediatric patients with solid tumors that have a neurotrophic receptor tyrosine kinase (NTRK) gene fusion without a known acquired resistance mutation; are metastatic or where surgical resection is likely to result in severe morbidity; and have no satisfactory alternative treatments or that have progressed following treatment.	NCT02122913<br>NCT02637687<br>NCT02576431<br>LOXO-TRK-14001<br>SCOUT<br>NAVIGATE	SAT	larotrectinib	NA	ORR<br>BOR (best overall response), DOR (duration of response), CBR (complete benefit rate), PFS, OS
84	nivolumab	In combination with ipilimumab, is indicated for the treatment of adults and pediatric patients 12	NCT04008030	RCT	nivolumab/	chemotherapy (oxaliplatin,	PFS

ID	Drug name	Indication	Confirmatory trial identifier(s)	Design	Experimental treatment	Confirmatory control arm	Primary endpoint(s)
		years and older with microsatellite instability-high (MSI-H) or DNA mismatch repair deficient (dMMR), metastatic colorectal cancer that has progressed following treatment with a fluoropyrimidine, oxaliplatin, and irinotecan.	CheckMate 8HW		nivolumab + ipilimumab	leucovorin, fluorouracil, irinotecan, bevacizumab, cetuximab)	
85	ipilimumab	In combination with nivolumab, is indicated for the treatment of adults and pediatric patients 12 years and older with microsatellite instability-high (MSI-H) or DNA mismatch repair deficient (dMMR), metastatic colorectal cancer that has progressed following treatment with a fluoropyrimidine, oxaliplatin, and irinotecan.	NCT04008030 CheckMate 8HW	RCT	nivolumab/ nivolumab + ipilimumab	chemotherapy (oxaliplatin, leucovorin, fluorouracil, irinotecan, bevacizumab, cetuximab)	PFS
86	acalabrutinib	Treatment of adult patients with mantle cell lymphoma (MCL) who have received at least one prior therapy.	NCT02972840 ECHO	RCT	acalabrutinib + bendamustine + rituximab	placebo + bendamustine + rituximab	PFS
87	nivolumab	For the treatment of adult and pediatric patients 12 years and older with microsatellite instability-high (MSI-H) or mismatch repair deficient (dMMR) metastatic colorectal cancer (CRC) that has progressed following treatment with a fluoropyrimidine, oxaliplatin, and irinotecan.	NCT04008030 CheckMate 8HW	RCT	nivolumab/ nivolumab + ipilimumab	chemotherapy (oxaliplatin, leucovorin, fluorouracil, irinotecan, bevacizumab, cetuximab)	PFS
88	nivolumab	Treatment of adult patients with classical Hodgkin lymphoma that has relapsed or progressed after: autologous hematopoietic stem cell transplantation (HSCT) and brentuximab vedotin, or 3 or more lines of systemic therapy that includes autologous HSCT.	NCT03907488 SWOG S1826	RCT	nivolumab + chemotherapy (doxorubicin, hydrochloride, vinblastine sulfate, dacarbazine, pegfilgrastim or filgrastim)	brentuximab vedotin + chemotherapy (doxorubicin, hydrochloride, vinblastine sulfate, dacarbazine, pegfilgrastim or filgrastim)	PFS
89	nivolumab	For the treatment of classical Hodgkin Lymphoma that has relapsed or progressed after autologous hematopoietic stem cell	NCT03907488 SWOG S1826	RCT	nivolumab + chemotherapy (doxorubicin, hydrochloride,	brentuximab vedotin + chemotherapy (doxorubicin	PFS

ID	Drug name	Indication	Confirmatory trial identifier(s)	Design	Experimental treatment	Confirmatory control arm	Primary endpoint(s)
		transplantation (HSCT) and post-transplantation brentuximab vedotin.			vinblastine sulfate, dacarbazine, may also receive pegfilgrastim or filgrastim)	hydrochloride, vinblastine sulfate, dacarbazine, pegfilgrastim or filgrastim)	
90	belinostat	Treatment of relapsed or refractory peripheral T-Cell Lymphoma (PTCL).	NCT06072131 CRESCENDO	RCT	belinostat + CHOP (cyclophosphamide, vincristine, doxorubicin, prednisone)	CHOP (cyclophosphamide, vincristine, doxorubicin, prednisone)	

Indications are ordered by accelerated approval status (withdrawn, verified, ongoing) and approval date. Within each status category, indications are listed from most to least recent

ORR: overall response rate; PFS: progression-free survival; RCT: randomised controlled trial; SAT: single-arm trial; OS: overall survival

Appendix table 3. Available data on overall survival.

ID	Drug name	Indication	Availability of OS benefit [a]	OS findings	Source
1	pembrolizumab	Metastatic small cell lung cancer (SCLC) with disease progression on or after platinum-based chemotherapy and at least one other prior line of therapy.	no	Treatment: 10.80 months<br>(95% CI: 9.20 to 12.90)<br>Control: 9.70 months<br>(95% CI: 8.60 to 10.70)<br>HR: 0.80 (95% CI: 0.64 to 0.98)	https://clinicaltrials.gov/study/NCT03066778<br><br>Time frame: 30.5 months
2	atezolizumab	In combination with paclitaxel protein-bound for unresectable locally advanced or metastatic triple-negative breast cancer whose tumors express PD-L1 (PD-L1 stained tumor-infiltrating immune cells of any intensity covering = 1% of the tumor area), as determined by an FDA-approved test.	no	Treatment: 22.05 months<br>(95% CI: 19.19 to 30.52)<br>Control: 28.29 months<br>(95% CI: 19.06 to NA)<br>HR: 1.11 (95% CI: 0.76 to 1.64)	https://clinicaltrials.gov/study/NCT03125902<br><br>Time frame: 26 months
3	duvelisib	Treatment of adult patients with relapsed or refractory follicular lymphoma (FL) after at least 2 prior systemic therapies.	unknown (*trial terminated*)	NA	https://clinicaltrials.gov/study/NCT02204982
4	nivolumab	Metastatic small cell lung cancer (SCLC) with progression after platinum-based chemotherapy and at least one other line of therapy.	no	Treatment: 10.18 months<br>(95% CI: 9.43 to 11.99)<br>Control: 9.56 months<br>(95% CI: 8.18 to 11.01)<br>HR: 0.81 (95% CI: 0.68 to 0.97)	https://clinicaltrials.gov/study/NCT02538666<br><br>Time frame: 73 months
5	pembrolizumab	For patients with recurrent or locally advanced or metastatic gastric or gastroesophageal junction (GEJ) adenocarcinoma whose tumors express PD-L1 [CPS ≥1] as determined by an FDA-approved test, with disease progression on/after two or more prior lines of therapy including fluoropyrimidine and platinum containing chemotherapy and if appropriate, HER2/NEU targeted therapy.	no	Treatment: 10.60 months<br>(95% CI: 7.70 to 13.80)<br>Control: 11.10 months<br>(95% CI: 9.20 to 12.80)<br>HR: 0.91 (95% CI: 0.69 to 1.18)	https://clinicaltrials.gov/study/NCT02494583<br><br>Time frame: 42 months
6	nivolumab	Hepatocellular carcinoma previously treated with sorafenib.	no	Treatment: 16.39 months<br>(95% CI: 13.93 to 18.37)<br>Control: 14.69 months<br>(95% CI: 11.89 to 17.22)<br>HR: 0.85 (95% CI: 0.71 to 1.02)	https://clinicaltrials.gov/study/NCT02576509<br><br>Time frame: 41 months (Jun 2019)

ID	Drug name	Indication	Availability of OS benefit [a]	OS findings	Source
7	durvalumab	Locally advanced or metastatic urothelial carcinoma that progressed during or following platinum-containing chemotherapy or within 12 months of neoadjuvant or adjuvant treatment with platinum-containing chemotherapy.	no	Treatment: 13.20 months (95% CI: 10.30 to 15.00)<br>Control: 12.10 months (95% CI: 10.90 to 14.00)<br>HR: 0.99 (95% CI: 0.83 to 1.17)	https://clinicaltrials.gov/study/NCT02516241<br><br>Time frame: 5 years (27 Jan 2020)
8	atezolizumab	Patients with locally advanced or metastatic urothelial carcinoma (mUC) who are not eligible for cisplatin-containing chemotherapy and whose tumors express PD-L1 as determined by an FDA approved test or who are not eligible for any platinum-containing chemotherapy regardless of PD-L1 status.	no	Treatment: 15.21 months (95% CI: 13.14 to 17.68)<br>Control: 13.34 months (95% CI: 11.89 to 15.61)<br>HR: 0.98 (95% CI: 0.82 to 1.16)	https://clinicaltrials.gov/study/NCT02807636<br><br>Time frame: 73 months
9	ibrutinib	Adult patients with marginal zone lymphoma (MZL) who require systemic therapy and have received at least one prior anti-CD20-based therapy.	unknown (*immature data*)	Treatment: 76.6% alive<br>Control: 68.5% alive<br>HR: NA	https://clinicaltrials.gov/study/NCT01974440<br><br>Time frame: 7 years
10	olaratumab	In combination with doxorubicin for adults with soft tissue sarcoma with a histologic subtype for which an anthracycline-containing regimen is appropriate and which is not amenable to curative treatment with radiotherapy or surgery.	no	Treatment: 20.37 months (95% CI: 17.84 to 22.90)<br>Control: 19.75 months (95% CI: 16.49 to 23.75)<br>HR: 1.05 (95% CI: 0.84 to 1.30)	https://clinicaltrials.gov/study/NCT02451943<br><br>Time frame: 35.8 months
11	atezolizumab	Locally advanced or metastatic urothelial carcinoma that progressed during or following platinum-containing chemotherapy or within 12 months of neoadjuvant or adjuvant treatment with platinum-containing chemotherapy.	no	Treatment: 8.60 months (95% CI: 7.80 to 9.60)<br>Control: 8.00 months (95% CI: 7.20 to 8.60)<br>HR: 0.85 (95% CI: 0.73 to 0.99)	https://clinicaltrials.gov/study/NCT02302807<br><br>Time frame: 25 months
12	panobinostat	In combination with bortezomib (BTZ) and dexamethasone (DEX) for the treatment of patients with multiple myeloma (MM) who have received at least 2 prior regimens, including BTZ and an immunomodulatory agent.	no	Treatment: 40.28 months (95% CI: 28.98 to 40.64)<br>Control: 35.78 months (95% CI: 35.02 to 44.81)<br>HR: 0.94 (95% CI: 0.78 to 1.14)	https://clinicaltrials.gov/study/NCT01023308<br><br>Time frame: 45 months
13	idelalisib	For the treatment of relapsed follicular B-cell Non-Hodgkin Lymphoma (FL) in patients who have received at least 2 prior systemic therapies and relapsed small lymphocytic lymphoma (SLL) in patients who have received at least 2 prior systemic therapies.	unknown (*single-arm trial*)	Treatment: 48.60 months (95% CI: 33.90 to 71.70)<br>Control: NA	https://clinicaltrials.gov/study/NCT01282424<br><br>Time frame: 7 years

ID	Drug name	Indication	Availability of OS benefit [a]	OS findings	Source
14	vincristine sulfate liposomal	Adults with Philadelphia (PH) chromosome negative (-) acute lymphoblastic leukemia (ALL) in second relapse or greater relapsed or whose disease has progressed following 2 or greater treatment lines of anti-leukemia therapies.	unknown (*trial terminated*)	NA	https://clinicaltrials.gov/study/NCT01439347
15	ibrutinib	For the treatment of adult patients with mantle cell lymphoma (MCL) who have received at least one prior therapy	no	Treatment: 55.0% alive<br>Control: 56.8% alive<br>HR: 1.07 (95% CI: 0.81 to 1.40)	https://clinicaltrials.gov/study/NCT01578707<br><br>Time frame: 7 years
16	ibrutinib	For the treatment of patients with mantle cell lymphoma (MCL).	no	Treatment: 55.0% alive<br>Control: 56.8% alive<br>HR: 1.07 (95% CI: 0.81 to 1.40)	https://clinicaltrials.gov/study/NCT01578707<br><br>Time frame: 7 years
17	erdafitinib	Treatment of adult patients with locally advanced or metastatic urothelial carcinoma (mUC), that has: susceptible FGFR3 or FGFR2 genetic alterations, and progressed during or following at least one line of prior platinum-containing chemotherapy, including within 12 months of neoadjuvant or adjuvant platinum-containing chemotherapy.	yes	Treatment: 12.10 months (95% CI: 10.30 to 16.40)<br>Control: 7.80 months (95% CI: 6.50 to 11.10)<br>HR: 0.64 (95% CI: 0.47 to 0.88)	Loriot, Y., et al. New England Journal of Medicine. 2023; 389 (21): 1961–1971.
18	pembrolizumab	Treatment of adults and pediatric patients with recurrent locally advanced or metastatic Merkel cell carcinoma (MCC).	unknown (*single-arm trial*)	Treatment: 24.30 months (95% CI: 12.40 to NA)<br>Control: NA	https://clinicaltrials.gov/study/NCT03783078<br><br>Time frame: 58 months
19	pembrolizumab	Treatment of patients with hepatocellular carcinoma (HCC) who have been previously treated with sorafenib.	yes	Treatment: 14.60 months (95% CI: 12.60 to 18.00)<br>Control: 13.00 months (95% CI: 10.50 to 15.10)<br>HR: 0.79 (95% CI: 0.63 to 0.99)	https://clinicaltrials.gov/study/NCT03062358<br><br>Time frame: 4 years
20	pembrolizumab	In combination with chemotherapy for locally recurrent unresectable or metastatic triple-negative breast cancer (TNBC) expressing PD L1 [CPS =10] as determined by an FDA-approved test.	yes	Treatment: 23.00 months (95% CI: 19.00 to 26.30)<br>Control: 16.10 months (95% CI: 12.60 to 18.80<br>HR: 0.73 (95% CI: 0.55 to 0.95)	https://clinicaltrials.gov/study/NCT02819518<br><br>Time frame: 53 months

ID	Drug name	Indication	Availability of OS benefit [a]	OS findings	Source
21	selpercatinib	Adult patients with metastatic RET fusion-positive non-small cell lung cancer (NSCLC).	unknown (*single-arm trial*)	NA	https://clinicaltrials.gov/study/NCT03157128
22	capmatinib	Adult patients with metastatic non-small cell lung cancer (mNSCLC) whose tumors have a mutation that leads to MET exon 14 skipping as detected by an FDA-approved test.	unknown (*single-arm trial)*	Treatment: 21.36 months (95% CI: 15.24 to 30.52) Control: NA	https://clinicaltrials.gov/study/NCT02414139 Time frame: 6 years
23	sacituzumab govitecan-hziy	Adults with metastatic triple-negative breast cancer (TNBC) following at least 2 prior therapies for metastatic disease.	yes	Treatment: 11.80 months (95% CI: 10.50 to 13.80) Control: 6.90 months (95% CI: 5.90 to 7.70) HR: 0.51 (95% CI: 0.42 to 0.63)	https://clinicaltrials.gov/study/NCT02574455 Time frame: 30.8 months
24	fam-trastuzumab deruxtecan-nxki	Adult patients with unresectable or metastatic HER2-positive breast cancer who have received two or more prior anti-HER2-based regimens in the metastatic setting.	yes	Treatment: 52.60 months (95% CI: 48.70 to NA) Control: 42.70 months (95% CI: 35.40 to NA) HR: 0.73 (95% CI: 0.56 to 0.94)	Cortés, J., et al. Nature Medicine. 2024; 30: 2208–2215.
25	enfortumab vedotin-ejfv	Adults with locally advanced or metastatic urothelial cancer who received a PD-1 or PD-L1 inhibitor and a platinum-containing chemotherapy in the neoadjuvant, locally advanced, or metastatic setting.	yes	Treatment: 12.88 months (95% CI: 10.58 to 15.21) Control: 8.97 months (95% CI: 8.05 to 10.74) HR: 0.70 (95% CI: 0.56 to 0.89)	https://clinicaltrials.gov/study/NCT03474107 Time frame: 11.1 months (15 Jul 2020)
26	pembrolizumab	In combination with lenvatinib for advanced endometrial carcinoma not MSI-H or dMMR, with progression following systemic therapy and not candidates for curative surgery or radiation.	yes	Treatment: 18.30 months (95% CI: 15.20 to 20.50) Control: 11.40 months (95% CI: 10.50 to 12.90) HR: 0.62 (95% CI: 0.51 to 0.75)	https://clinicaltrials.gov/study/NCT03517449 Time frame: 2 years 5 months (26 Oct 2020)
27	lenvatinib	In combination with pembrolizumab for advanced endometrial carcinoma not MSI-H or dMMR, with progression following systemic therapy and not candidates for curative surgery or radiation.	yes	Treatment: 18.30 months (95% CI: 15.20 to 20.50) Control: 11.40 months (95% CI: 10.50 to 12.90) HR: 0.62 (95% CI: 0.51 to 0.75)	https://clinicaltrials.gov/study/NCT03517449 Time frame: 2 years 5 months (26 Oct 2020)
28	selinexor	In combination with dexamethasone for adults with relapsed/refractory multiple myeloma after at least 4 prior	no	Treatment: 36.67 months (95% CI: 30.19 to NA)	https://clinicaltrials.gov/study/NCT03110562

ID	Drug name	Indication	Availability of OS benefit [a]	OS findings	Source
		therapies and refractory to at least 2 proteasome inhibitors, 2 immunomodulatory agents, and an anti-CD38 monoclonal antibody.		Control: 32.76 months<br>(95% CI: 27.83 to NA)<br>HR: 0.88 (95% CI: 0.63 to 1.22)	Time frame: 45 months
29	polatuzumab vedotin- piiq	In combination with bendamustine and a rituximab product for adult patients with relapsed or refractory diffuse large B-cell lymphoma (DLBCL), not otherwise specified, after at least two prior therapies.	no	Treatment: 88.70%<br>(95% CI: 85.70 to 91.60)<br>Control: 88.60%<br>(95% CI: 85.60 to 91.60)<br>HR: 0.94 (95% CI: 0.65 to 1.37)	Tilly, H., et al. New England Journal of Medicine. 2022; 386 (4): 351–363.<br><br>Time frame: 2 years
30	venetoclax	In combination with azacitidine or decitabine or low-dose cytarabine for newly diagnosed acute myeloid leukemia (AML) in adults 75 years or older, or who have comorbidities that preclude use of intensive induction chemotherapy.	yes	Treatment: 14.70 months<br>(95% CI: 12.10 to 18.70)<br>Control: 9.60 months<br>(95% CI: 7.40 to 12.70)<br>HR: 0.58 (95% CI: 0.47 to 0.72)	https://clinicaltrials.gov/study/NCT02993523<br><br>Time frame: 4.8 years (1 Dec 2021)
31	lorlatinib	ALK-positive metastatic non-small cell lung cancer (NSCLC) that has progressed on: crizotinib and at least one other ALK inhibitor for metastatic disease; or alectinib as the first ALK inhibitor therapy for metastatic disease; or ceritinib as the first ALK inhibitor therapy for metastatic disease.	unknown (*immature data*)	Treatment: NA<br>Control: NA<br>HR: 0.72 (0.41 to 1.25)	https://clinicaltrials.gov/study/NCT03052608<br><br>Time frame: 33 months
32	pembrolizumab	Adult and pediatric patients with refractory primary mediastinal large B-cell lymphoma, or who have relapsed after 2 or more prior lines of therapy.	unknown (*immature data*)	NA	https://clinicaltrials.gov/study/NCT02684292
33	pembrolizumab	Treatment of patients with recurrent or metastatic cervical cancer with disease progression on or after chemotherapy whose tumors express PD-L1 (CPS >/=1) as determined by an FDA approved test.	yes	Treatment: 28.60 months<br>(95% CI: 22.10 to 38.00)<br>Control: 16.50 months<br>(95% CI: 14.50 to 20.00)<br>HR: 0.60 (95% CI: 0.49 to 0.74)	https://clinicaltrials.gov/study/NCT03635567<br><br>Time frame: 46 months
34	pemetrexed disodium	In combination with pembrolizumab and carboplatin for first-line treatment of metastatic non-squamous non-small cell lung cancer (NSCLC).	yes	Treatment: 22.00 months<br>(95% CI: 19.50 to 25.20)<br>Control: 10.70 months<br>(95% CI: 8.70 to 13.60)<br>HR: 0.56 (95% CI: 0.45 to 0.70)	Gadgeel, S., et al. Journal of Clinical Oncology. 2020; 38 (14): 1505–1517.

ID	Drug name	Indication	Availability of OS benefit [a]	OS findings	Source
					https://doi.org/10.1200/JCO.19.03136
35	blinatumomab	Treatment of CD19-positive B-cell precursor acute lymphoblastic leukemia (ALL) in first or second complete remission with minimal residual disease (MRD) greater than or equal to 0.1% in adults and children.	yes	Treatment: 85.00% alive<br>Control: 68.00% alive<br>HR: 0.41 (95% CI: 0.23 to 0.73)	Litzow, M. R., et al. New England Journal of Medicine. 2024; 391 (4): 320-333.<br><br>Time frame: 3 years
36	bosutinib	Adults with newly diagnosed chronic phase Philadelphia chromosome positive chronic myeloid leukemia (CML).	no	Treatment: 94.50% alive<br>(95% CI: 90.80 to 96.70)<br>Control: 94.60% alive<br>(95% CI: 91.00 to 96.80)<br>HR: 0.95 (95% CI: 0.45 to 1.99)	Cortes, J. E., et al. Journal of Clinical Oncology. 2018; 36 (3): 231–237.<br><br>Time frame: 60 months
37	pembrolizumab	Treatment of adult and pediatric patients with unresectable or metastatic, microsatellite instability-high (MSI-H) or mismatch repair deficient (dMMR) solid tumors that have progressed following prior treatment and who have no satisfactory alternative treatment options, or metastatic MSI-H or dMMR colorectal cancer that have progressed following treatment with a fluoropyrimidine, oxaliplatin and irinotecan.	no	Treatment: NA months<br>(95% CI: 49.20 to NA)<br>Control: 36.70 months<br>(95% CI: 27.60 to NA)<br>HR: 0.74 (95% CI: 0.53 to 1.03)	https://clinicaltrials.gov/study/NCT02563002<br><br>Time frame: 59 months
38	pembrolizumab	Locally advanced or metastatic urothelial carcinoma ineligible for cisplatin-containing chemotherapy.	no	Treatment: 15.60 months<br>(95% CI: 12.10 to 17.90)<br>Control: 14.30 months<br>(95% CI: 12.30 to 16.70)<br>HR: 0.92 (95% CI: 0.77 to 1.11)	https://clinicaltrials.gov/study/NCT02853305<br><br>Time frame: 42 months
39	pembrolizumab	In combination with pemetrexed and carboplatin for first-line treatment of metastatic non-squamous non-small cell lung cancer (NSCLC).	yes	Treatment: 22.00 months<br>(95% CI: 19.50 to 25.20)<br>Control: 10.70 months<br>(95% CI: 8.70 to 13.60)<br>HR: 0.56 (95% CI: 0.45 to 0.70)	Gadgeel, S., et al. Journal of Clinical Oncology. 2020; 38 (14): 1505–1517.
40	avelumab	Locally advanced or metastatic urothelial carcinoma following disease progression on platinum-containing chemotherapy or disease progression within 12 months of	yes	Treatment: 23.80 months<br>(95% CI: 19.90 to 28.80)<br>Control: 15.00 months	Powles, T., et al. Journal of Clinical

ID	Drug name	Indication	Availability of OS benefit [a]	OS findings	Source
		neoadjuvant or adjuvant treatment with platinum-containing chemotherapy.		(95% CI: 13.50 to 18.20)<br>HR: 0.76 (95% CI: 0.63 to 0.91)	Oncology. 2023;41(19): 3333-3344.
41	brigatinib	Patients with ALK-positive metastatic non-small cell lung cancer (NSCLC) that have progressed or are intolerant to crizotinib.	no	Treatment: 66.00%<br>(95% CI: 56.00 to 74.00)<br>Control: 60.00%<br>(95% CI: 51.00 to 68.00)<br>HR: 0.81 (95% CI: 0.53 to 1.22)	Camidge, D. R., et al. Journal of Thoracic Oncology. 2021;16 (12):2091–2108.<br><br>Time frame: 4 years
42	avelumab	Treatment of adults and pediatrics patients 12 years and older with metastatic Merkel cell carcinoma (MCC).	unknown (*single-arm trial*)	Treatment: 20.30 months<br>(95% CI: 0.50 to 65.80)<br>Control: NA	https://clinicaltrials.gov/study/NCT02155647<br><br>Time frame: 396 weeks
43	pembrolizumab	Adult and pediatric patients with refractory classical Hodgkin Lymphoma or who have relapsed after 3 or more prior lines of therapy.	unknown (*immature data*)	NA	https://clinicaltrials.gov/study/NCT02684292
44	nivolumab	Locally advanced or metastatic urothelial carcinoma that: progressed during or following platinum-containing chemotherapy; progressed within 12 months of neoadjuvant or adjuvant platinum-containing chemotherapy.	yes	Treatment: 69.50 months<br>(95% CI: 58.10 to NA)<br>Control: 50.10 months<br>(95% CI: 38.20 to NA)<br>HR: 0.76 (95% CI: 0.61 to 0.96)	Bajorin, D. F., et al. New England Journal of Medicine. 2021; 384 (22): 2102–2114.
45	rucaparib	Deleterious BRCA mutation (germline and/or somatic) associated advanced ovarian cancer treated with 2 or more chemotherapies.	no	Treatment: 36.00 months<br>(95% CI: 32.80 to 39.40)<br>Control: 43.20 months<br>(95% CI: 38.10 to 46.90)<br>HR: 1.00 (95% CI: 0.81 to 1.22)	Coleman, R. L., et al. International Journal of Gynecological Cancer. 2021; 32(Suppl 3): A3–A4.
46	pembrolizumab	Recurrent or metastatic head and neck squamous cell carcinoma that progressed on or after platinum-containing chemotherapy.	yes	Treatment: 11.50 months<br>(95% CI: 10.30 to 13.40)<br>Control: 10.70 months<br>(95% CI: 9.30 to 11.70)<br>HR: 0.83 (95% CI: 0.70 to 0.99)	https://clinicaltrials.gov/study/NCT02358031<br><br>Time frame: 47 months
47	venetoclax	Chronic lymphocytic leukemia with 17P deletion as detected by an FDA-approved test, after at least one prior therapy.	yes	Treatment: 70.2% alive<br>(95% CI: 56.8% to 83.6%)<br>Control: 60.7% alive	https://pubmed.ncbi.nlm.nih.gov/35605176/

ID	Drug name	Indication	Availability of OS benefit [a]	OS findings	Source
				(95% CI: 46.0% to 75.5%)<br>HR: NA	Time frame: 5 years
48	nivolumab	1) In combination with ipilimumab for unresectable or metastatic melanoma to remove the restriction for treatment of only patients with BRAF wild-type melanoma;<br>2) As a single agent for BRAF V600 mutation positive unresectable or metastatic melanoma to remove the restriction that such patients should have disease progression following ipilimumab and a BRAF inhibitor.	yes	Treatment: 37.30 months<br>(95% CI: 25.40 to 51.60)<br>Control: 11.20 months<br>(95% CI: 9.60 to 13.00)<br>HR: 0.50 (95% CI: 0.40 to 0.63)	https://clinicaltrials.gov/study/NCT01721772<br><br>Time frame: 94 months
49	alectinib	ALK-positive metastatic non-small cell lung cancer (NSCLC) that progressed on or is intolerant to crizotinib.	unknown (*immature data*)	Treatment: 62.5% alive<br>(95% CI: 54.3% to 70.8%)<br>Control: 45.5% alive<br>(95% CI: 33.6% to 57.4%)	Mok, T., et al. Annals of Oncology. 2020; 31(8): 1056–1064.<br><br>Time frame: 5 years
50	daratumumab	Multiple myeloma after at least 3 prior lines of therapy including a proteasome inhibitor and an immunomodulatory agent or double refractory to a proteasome inhibitor and an immunomodulatory agent.	yes	Treatment: 67.58 months<br>(95% CI: 53.13 to 80.53)<br>Control: 51.84 months<br>(95% CI: 43.99 to 60.02)<br>HR: 0.73 (95% CI: 0.58 to 0.91)	https://clinicaltrials.gov/study/NCT02076009<br><br>Time frame: 21 months
51	osimertinib	Metastatic EGFR T790M mutation-positive non-small cell lung cancer (NSCLC), as detected by an FDA-approved test, that progressed on or after EGFR TKI therapy.	no	Treatment: 26.80 months<br>(95% CI: 23.50 to 31.50)<br>Control: 22.50 months<br>(95% CI: 20.20 to 28.80)<br>HR: 0.87 (95% CI: 0.67 to 1.13)	https://clinicaltrials.gov/study/NCT02151981<br><br>Time frame: 43 months
52	pembrolizumab	Metastatic PD-L1 positive non-small cell lung cancer (NSCLC), as determined by an FDA-approved test, with progression on or after platinum-containing chemotherapy.	yes	Treatment: 17.30 months<br>(95% CI: 11.80 to NA)<br>Control: 8.20 months<br>(95% CI: 6.40 to 10.70)<br>HR: 0.50 (0.36 to 0.70)	https://clinicaltrials.gov/study/NCT01905657<br><br>Time frame: 24 months (30 Sep 2015)
53	nivolumab	In combination with ipilimumab for BRAF V600 wild-type unresectable or metastatic melanoma.	yes	Treatment: 39.10 months<br>(95% CI: 27.50 to 84.60)<br>Control: 18.50 months<br>(95% CI: 14.10 to 22.70)<br>HR: 0.58 (95% CI: 0.46 to 0.73)	Wolchok, J. D., et al. New England Journal of Medicine. 2017; 377(14): 1345–1356.

ID	Drug name	Indication	Availability of OS benefit [a]	OS findings	Source
54	palbociclib	In combination with letrozole for postmenopausal women with ER-positive, HER2-negative advanced breast cancer as initial endocrine-based therapy for metastatic disease.	no	Treatment: 53.80 months<br>(95% CI: 49.80 to 59.20)<br>Control: 49.80 months<br>(95% CI: 42.30 to 56.40)<br>HR: 0.92 (95% CI: 0.76 to 1.12)	https://clinicaltrials.gov/study/NCT01740427<br><br>Time frame: 10.51 years
55	nivolumab	Unresectable or metastatic melanoma and progression following ipilimumab and, if BRAF V600 mutation positive, a BRAF inhibitor.	no	Treatment: 15.74 months<br>(95% CI: 12.88 to 19.88)<br>Control: 14.39 months<br>(95% CI: 11.66 to 18.17)<br>HR: 0.86 (95% CI: 0.68 to 1.08)	https://clinicaltrials.gov/study/NCT01721746<br><br>Time frame: 96 months
56	olaparib	Deleterious or suspected deleterious germline BRCA-mutated advanced ovarian cancer after treatment with 3 or more lines of chemotherapy.	no	Treatment: 52.40 months<br>(95% CI: 41.50 to 61.40)<br>Control: 37.40 months<br>(95% CI: 29.80 to 44.20)<br>HR: 0.71 (95% CI: 0.52 to 0.97)	Poveda, A., et al. Lancet Oncology. 2021;22(5): 620–631.
57	blinatumomab	Philadelphia chromosome negative relapsed or refractory B-cell precursor acute lymphoblastic leukemia.	yes	Treatment: 7.70<br>(95% CI: 5.60 to 9.60)<br>Control: 4.00 (<br>95% CI: 2.90 to 5.30)<br>HR: 0.71 (95% CI: 0.55 to 0.93)	https://clinicaltrials.gov/study/NCT02013167<br><br>Time frame: 11.7 months (4 Jan 2016)
58	pembrolizumab	Unresectable or metastatic melanoma and disease progression following ipilimumab and, if BRAF V600 mutation positive, a BRAF inhibitor.	yes	Treatment: 32.70 months<br>(95% CI: 24.50 to 41.60)<br>Control: 15.90 months<br>(95% CI: 13.30 to 22.00)<br>HR: 0.70 (95% CI: 0.58 to 0.83)	Robert, C., et al. Journal of Clinical Oncology. 2023; 41(24).
59	ceritinib	ALK-positive metastatic non-small cell lung cancer (NSCLC) that progressed on or is intolerant to crizotinib.	unknown (*immature data*)	Treatment: 70.6% alive<br>(95% CI: 62.2% to 77.5%)<br>Control: 58.2% alive<br>(95% CI: 47.6% to 67.5%)	Soria, J.-C., et al. Lancet. 2017; 389(10072), 917–929.<br><br>Time frame: 24 months
60	ibrutinib	Chronic lymphocytic leukemia after at least one prior therapy.	no	Treatment: 67.70 months<br>(95% CI: 61.00 to NA)<br>Control: 65.10 months<br>(95% CI: 50.60 to NA)<br>HR: 0.81 (95% CI: 0.60 to 1.09)	https://clinicaltrials.gov/study/NCT01578707<br><br>Time frame: 6 years

ID	Drug name	Indication	Availability of OS benefit [a]	OS findings	Source
61	dabrafenib	In combination with trametinib for unresectable or metastatic melanoma with BRAF V600E or V600K mutations, as detected by an FDA-approved test.	no	Treatment: 25.80 months (95% CI: 19.20 to 38.20)<br>Control: 18.70 months (95% CI: 15.20 to 23.10)<br>HR: 0.81 (95% CI: 0.64 to 1.02)	https://clinicaltrials.gov/study/NCT01584648<br><br>Time frame: 6 years
62	trametinib	In combination with dabrafenib for unresectable or metastatic melanoma with BRAF V600E or V600K mutations, as detected by an FDA-approved test.	no	Treatment: 25.80 months (95% CI: 19.20 to 38.20)<br>Control: 18.70 months (95% CI: 15.20 to 23.10)<br>HR: 0.81 (95% CI: 0.64 to 1.02)	https://clinicaltrials.gov/study/NCT01584648<br><br>Time frame: 6 years
63	pertuzumab	In combination with trastuzumab and docetaxel for neoadjuvant treatment of HER2-positive locally advanced inflammatory or early-stage breast cancer (either greater than 2 cm in diameter or node-positive) as part of a complete treatment regimen for early breast cancer.	no	Treatment: 92.70%<br>Control: 92.00%<br>HR: 0.83 (0.68 to 1.02)	Loibl, S., et al. Journal of Clinical Oncology. 2024; 42(31).<br><br>Time frame: 8 years
64	pomalidomide	Multiple myeloma after at least 2 prior therapies including lenalidomide and bortezomib and disease progression on or within 60 days of completion of the last therapy.	yes	Treatment: 56.10 weeks (95% CI: 47.70 to 67.40)<br>Control: 35.30 weeks (95% CI: 29.90 to 39.90)<br>HR: 0.74 (95% CI: 0.60 to 0.91)	https://clinicaltrials.gov/study/NCT01311687<br><br>Time frame: 324 weeks (29 Aug 2017)
65	ponatinib	Adults with chronic phase, accelerated phase, or blast phase chronic myeloid leukemia resistant or intolerant to TKI therapy or Philadelphia chromosome positive acute lymphoblastic leukemia resistant or intolerant to prior TKI therapy.	unknown (*single-arm trial*)	Treatment: 73.00% (66.00 to 79.00)<br>Control: NA	Cortes, J. E., et al. Blood. 2018; 132(4): 393–404.<br><br>Time frame: 5 years
66	omacetaxine mepesuccinate	Adults with chronic or accelerated phase chronic myeloid leukemia with resistance or intolerance to 2 or more TKIs.	unknown (*single-arm trial*)	Treatment: 49.31 months (95% CI: 24.97 to NA)<br>Control: NA<br>(*chronic phase*)	https://clinicaltrials.gov/study/NCT00375219<br><br>Time frame: 4 years
67	carfilzomib	Multiple myeloma after at least 2 prior therapies including bortezomib and an immunomodulatory agent and disease progression on or within 60 days of completion of the last therapy.	yes	Treatment: 48.80 months<br>Control: 42.30 months<br>HR: 0.79 (95% CI: 0.62 to 0.99)	Siegel, D. S., et al. Journal of Clinical Oncology. 2018; 36(8).
68	selinexor	Treatment of adult patients with relapsed or refractory diffuse large B-cell lymphoma (DLBCL), not otherwise	unknown (*trial recruiting*)	NA	https://clinicaltrials.gov/study/NCT04442022

ID	Drug name	Indication	Availability of OS benefit [a]	OS findings	Source
		specified, including DLBCL arising from follicular lymphoma (FL), after at least 2 lines of systemic therapy.			
69	tazemetostat	Treatment of adult patients with relapsed or refractory (R/R) follicular lymphoma (FL) whose tumors are positive for an EZH2 mutation as detected by an FDA-approved test and who have received at least 2 prior systemic therapies. Treatment of adult patients with R/R FL who have no satisfactory alternative treatment options.	unknown (*trial recruiting*)	NA	https://clinicaltrials.gov/study/NCT04224493
70	pembrolizumab	Treatment of adult and pediatric patients with unresectable or metastatic tumor mutational burden high (TMB H) [=10 mutations/megabase (mut/Mb)] solid tumors, as determined by an FDA-approved test, that have progressed following prior treatment and who have no satisfactory alternative treatment options.	unknown (*single-arm trial*)	Treatment: 11.70 months (95% CI: 9.10 to 19.10) Control: NA	Marabelle, A., et al. Lancet Oncology. 2020; 21(10): 1353–1365.
71	lurbinectedin	Treatment of adult patients with metastatic small cell lung cancer (SCLC) with disease progression on or after prior platinum-based chemotherapy.	no	Treatment: 8.60 months (95% CI: 7.10 to 9.40) Control: 7.60 months (95% CI: 6.60 to 8.20) HR: 0.97 (95% CI: 0.82 to 1.15)	https://clinicaltrials.gov/study/NCT02566993<br><br>Time frame: 3.5 years
72	rucaparib	Treatment of adult patients with a deleterious BRCA mutation (germline and/or somatic)-associated metastatic castration-resistant prostate cancer (mCRPC) who have been treated with androgen receptor-directed therapy and a taxane-based chemotherapy.	unknown (*immature data*)	Treatment: 24.30 months (95% CI: 19.90 to 25.70) Control: 20.80 months (95% CI: 16.30 to 23.10) HR: 0.81 (95% CI: 0.58 to 1.12)	https://clinicaltrials.gov/study/NCT02975934<br><br>Time frame: 5 years
73	pomalidomide	Treatment of patients with AIDS-related Kaposi's Sarcoma after failure of highly active antiretroviral therapy (HAART).	unknown (*not measured*)	NA	https://clinicaltrials.gov/study/NCT04577755
74	pomalidomide	Treatment of Kaposi's Sarcoma in patients who are HIV-negative.	unknown (*not measured*)	NA	https://clinicaltrials.gov/study/NCT04577755
75	selpercatinib	Adult and pediatric patients 12 years of age and older with advanced or metastatic RET-mutant medullary thyroid cancer (MTC) who require systemic therapy.	unknown (*immature data*)	Treatment: 95.5% alive (95% CI: 90.1% to 98.0%) Control: 92.8% alive (95% CI: 83.0% to 97.1%) HR: 0.37 (95% CI: 0.15 to 0.95)	Hadoux, J., et al. New England Journal of Medicine. 2023; 389(20): 1851-1861.<br><br>Time frame: 18 months

ID	Drug name	Indication	Availability of OS benefit [a]	OS findings	Source
76	selpercatinib	Adult and pediatric patients 12 years of age and older with advanced or metastatic RET fusion-positive thyroid cancer who require systemic therapy and who are radioactive iodine-refractory (if radioactive iodine is appropriate).	unknown (*immature data*)	Treatment: NA months (95% CI: 25.30 to NA) Control: NA	Wirth, L. J., et al. Journal of Clinical Oncology. 2024; 42(27): 3187–3195.
77	pemigatinib	Treatment of adults with previously treated unresectable locally advanced or metastatic cholangiocarcinoma with a fibroblast growth factor receptor 2 (FGFR2) fusion or other rearrangement as detected by an FDA-approved test.	unknown (*single-arm trial*)	NA	https://clinicaltrials.gov/study/NCT03656536
78	ipilimumab	In combination with nivolumab, for the treatment of patients with hepatocellular carcinoma (HCC) who have been previously treated with sorafenib.	yes	Treatment: 23.70 months (95% CI: 18.80 to 29.40) Control: 20.60 months (95% CI: 17.50 to 22.50) HR: 0.79 (95% CI: 0.65 to 0.96)	Galle, P. R., et al. Journal of Clinical Oncology. 2024; 42(17_suppl).
79	nivolumab	In combination with ipilimumab, for the treatment of patients with hepatocellular carcinoma (HCC) who have been previously treated with sorafenib.	yes	Treatment: 23.70 months (95% CI: 18.80 to 29.40) Control: 20.60 months (95% CI: 17.50 to 22.50) HR: 0.79 (95% CI: 0.65 to 0.96)	Galle, P. R., et al. Journal of Clinical Oncology. 2024; 42(17_suppl).
80	tazemetostat	Treatment of adults and pediatric patients aged 16 years and older with metastatic or locally advanced epithelioid sarcoma not eligible for complete resection.	unknown (*trial recruiting*)	NA	https://clinicaltrials.gov/study/NCT04204941
81	zanubrutinib	Treatment of adult patients with mantle cell lymphoma (MCL) who have received at least one prior therapy.	unknown (*trial ongoing*)	NA	https://clinicaltrials.gov/study/NCT04002297
82	entrectinib	Treatment of adult and pediatric patients 12 years of age and older with solid tumors that have a neurotrophic tyrosine receptor kinase (NTRK) gene fusion without a known acquired resistance mutation, are metastatic or where surgical resection is likely to result in severe morbidity, and have progressed following treatment or have no satisfactory alternative therapy.	unknown (*single-arm trial*)	Treatment: 37.10 months (95% CI: 27.20 to NA) Control: NA	Krzakowski, M. J., et al. Journal of Clinical Oncology. 2022; 40(16_suppl).
83	larotrectinib	Treatment of adult and pediatric patients with solid tumors that have a neurotrophic receptor tyrosine kinase (NTRK) gene fusion without a known acquired resistance mutation; are metastatic or where surgical resection is likely to result in severe morbidity; and have no	unknown (*single-arm trial*)	Treatment: 48.70 months (95% CI: 38.50 to NA) Control: NA	Hong, D. S., et al. Journal of Clinical Oncology. 2023; 41(16_suppl).

ID	Drug name	Indication	Availability of OS benefit [a]	OS findings	Source
		satisfactory alternative treatments or that have progressed following treatment.			
84	nivolumab	In combination with ipilimumab, is indicated for the treatment of adults and pediatric patients 12 years and older with microsatellite instability-high (MSI-H) or DNA mismatch repair deficient (dMMR), metastatic colorectal cancer that has progressed following treatment with a fluoropyrimidine, oxaliplatin, and irinotecan.	unknown (*trial recruiting*)	NA	https://clinicaltrials.gov/study/NCT06072131
85	ipilimumab	In combination with nivolumab, is indicated for the treatment of adults and pediatric patients 12 years and older with microsatellite instability-high (MSI-H) or DNA mismatch repair deficient (dMMR), metastatic colorectal cancer that has progressed following treatment with a fluoropyrimidine, oxaliplatin, and irinotecan.	unknown (*trial recruiting*)	NA	https://clinicaltrials.gov/study/NCT04008030
86	acalabrutinib	Treatment of adult patients with mantle cell lymphoma (MCL) who have received at least one prior therapy.	unknown (*trial recruiting*)	Treatment: 59.20 months (95% CI: 36.50 to NA)<br>Control: NA	Le Gouill, S., et al. Haematologica. 2023; 109(1), 343–350.
87	nivolumab	For the treatment of adult and pediatric patients 12 years and older with microsatellite instability-high (MSI-H) or mismatch repair deficient (dMMR) metastatic colorectal cancer (CRC) that has progressed following treatment with a fluoropyrimidine, oxaliplatin, and irinotecan.	unknown (*not available*)	NA	https://clinicaltrials.gov/study/NCT04008030
88	nivolumab	Treatment of adult patients with classical Hodgkin lymphoma that has relapsed or progressed after: autologous hematopoietic stem cell transplantation (HSCT) and brentuximab vedotin; or 3 or more lines of systemic therapy that includes autologous HSCT.	no	Treatment: 99.00%<br>(95% CI: 96.00 to 99.00)<br>Control: 98.00%<br>(95% CI: 97.00 to 100.00)<br>HR: 0.39 (95% CI: 0.15 to 1.03)	Herrera, A. F., et al. New England Journal of Medicine. 2024; 391(15), 1379–1389.<br><br>Time frame: 2 years
89	nivolumab	For the treatment of classical Hodgkin Lymphoma that has relapsed or progressed after autologous hematopoietic stem cell transplantation (HSCT) and post-transplantation brentuximab vedotin.	no	Treatment: 99.00%<br>(95% CI: 96.00 to 99.00)<br>Control: 98.00%<br>(95% CI: 97.00 to 100.00)<br>HR: 0.39 (95% CI: 0.15 to 1.03)	Herrera, A. F., et al. New England Journal of Medicine. 2024; 391(15), 1379–1389.<br><br>Time frame: 2 years
90	belinostat	Treatment of relapsed or refractory peripheral T-Cell Lymphoma (PTCL).	unknown (*not available*)	Treatment: 7.90 months<br>(95% CI: 6.10 to 13.90)	O'Connor, O. A., et al. Journal of Clinical

ID	Drug name	Indication	Availability of OS benefit [a]	OS findings	Source
				Control: NA	Oncology. 2015; 33(23), 2492–2499.

Indications are ordered by accelerated approval status (withdrawn, verified, ongoing) and approval date. Within each status category, indications are listed from most to least recent.

[a] Availability of statistically significant evidence of overall survival benefit.

Appendix table 4. List of drugs and comparators considered for the calculation of Medicare spending.

ID	Drug name	Confirmatory trial name	Confirmatory trial identifier	Trial arm	Drug
1	pembrolizumab	KEYNOTE-604	NCT03066778	Treatment Arm 1	Pembrolizumab, Etoposide, Carboplatin
				Treatment Arm 2	Pembrolizumab, Etoposide, Cisplatin
				Control Arm 1	Etoposide, Carboplatin
				Control Arm 2	Etoposide, Cisplatin
2	atezolizumab	MO39196 (IMpassion131)	NCT03125902	Treatment Arm	Atezolizumab, Paclitaxel
				Control Arm	Paclitaxel
3	duvelisib	DYNAMO +R	NCT02204982	Treatment Arm	Duvelisib, Rituximab
				Control Arm	Rituximab
4	nivolumab	CheckMate-451	NCT02538666	Treatment Arm	Nivolumab
				Control Arm	Placebo
5	pembrolizumab	KEYNOTE-062	NCT02494583	Treatment Arm	Pembrolizumab
				Control Arm	Cisplatin, Fluorouracil, Capecitabine
6	nivolumab	CheckMate-459	NCT02576509	Treatment Arm	Nivolumab
				Control Arm	Sorafenib
7	durvalumab	DANUBE	NCT02516241	Treatment Arm	Durvalumab
				Control Arm 1	Gemcitabine, Cisplatin
				Control Arm 2	Gemcitabine, Carboplatin
8	atezolizumab	IMvigor130	NCT02807636	Treatment Arm 1	Atezolizumab, Gemcitabine, Carboplatin
				Treatment Arm 2	Atezolizumab, Gemcitabine, Cisplatin
				Control Arm 1	Gemcitabine, Carboplatin

				Control Arm 2	Gemcitabine, Cisplatin
9	ibrutinib	SELENE	NCT01974440	Treatment Arm 1	Ibrutinib, Rituximab, Bendamustine
				Treatment Arm 2	Ibrutinib, Rituximab, Cyclophosphamide, Doxorubicin, Vincristine, Prednisone
				Control Arm 1	Rituximab, Bendamustine
				Control Arm 2	Rituximab, Cyclophosphamide, Doxorubicin, Vincristine, Prednisone
10	olaratumab	ANNOUNCE	NCT02451943	Treatment Arm	Olaratumab, Doxorubicin
				Control Arm	Doxorubicin
11	atezolizumab	GO29294	NCT02302807	Treatment Arm	Atezolizumab
				Control Arm 1	Vinflunine
				Control Arm 2	Paclitaxel
				Control Arm 3	Docetaxel
12	panobinostat	withdrawn indication, no confirmatory trial	Pivotal study: NCT01023308	Treatment Arm	Panobinostat, Bortezomib, Dexamethasone
				Control Arm	Bortezomib, Dexamethasone
13	idelalisib	withdrawn indication, no confirmatory trial	Pivotal study: NCT01282424	Treatment Arm	Idelalisib
			Comparator trial: NCT01059630	Comparator Control	Bendamustine
14	vincristine sulfate liposomal	TTX404 (Terminated)	NCT01439347	Treatment Arm	Vincristine Sulfate Injection
				Comparator Control	Vincristine
15	ibrutinib	PCI-32765MCL3002 (SHINE)	NCT01776840 NCT01776840	Treatment Arm	Ibrutinib, Bendamustine, Rituximab
				Control Arm	Bendamustine, Rituximab
16	ibrutinib	PCI-32765MCL3002 (SHINE)	NCT01776840	Treatment Arm	Ibrutinib, Bendamustine, Rituximab
				Control Arm	Bendamustine, Rituximab
17	erdafitinib	Study BLC3001	NCT03390504	Treatment Arm	Erdafitinib

				Control Arm	Vinflunine, Docetaxel
18	pembrolizumab	KEYNOTE-913	NCT03783078	Treatment Arm	Pembrolizumab
				Comparator Control	Avelumab
19	pembrolizumab	KEYNOTE-394	NCT03062358	Treatment Arm	Pembrolizumab
				Control Arm	Best supportive care
20	pembrolizumab	KEYNOTE-355	NCT02819518	Treatment Arm 1	Pembrolizumab, Nab-paclitaxel
				Treatment Arm 2	Pembrolizumab, paclitaxel
				Treatment Arm 3	Pembrolizumab, Gemcitabine, Carboplatin
				Control Arm 1	Nab-paclitaxel
				Control Arm 2	Paclitaxel
				Control Arm 3	Gemcitabine, Carboplatin
21	selpercatinib	LIBRETTO-001	NCT03157128	Treatment Arm	Selpercatinib
		LIBRETTO-431	NCT04194944	Comparator Control 1	Pemetrexed, Carboplatin
			NCT04194944	Comparator Control 2	Pemetrexed, Carboplatin, Pembrolizumab
			NCT04194944	Comparator Control 3	Pemetrexed, Cisplatin
			NCT04194944	Comparator Control 4	Pemetrexed, Cisplatin, Pembrolizumab
22	capmatinib	GEOMETRY mono-1	NCT02414139	Treatment Arm	Capmatinib
				Comparator Control	Docetaxel
23	sacituzumab govitecan-hziy	ASCENT	NCT02574455	Treatment Arm	Sacituzumab Govitecan
				Control Arm 1	Eribulin
				Control Arm 2	Capecitabine
				Control Arm 3	Gemcitabine

				Control Arm 4	Vinorelbine
24	fam-trastuzumab deruxtecan-nxki	DESTINY-Breast03	NCT03529110	Treatment Arm	Trastuzumab deruxtecan
				Control Arm	Ado-trastuzumab emtansine
25	enfortumab vedotin-ejfv	EV-301	NCT03474107	Treatment Arm	Enfortumab Vedotin
				Control Arm 1	Docetaxel
				Control Arm 2	Vinflunine
				Control Arm 3	Paclitaxel
26	pembrolizumab	KEYNOTE-775	NCT03517449	Treatment Arm	Pembrolizumab, Lenvatinib
				Control Arm 1	Paclitaxel
				Control Arm 2	Doxorubicin
27	lenvatinib	KEYNOTE-775	NCT03517449	Treatment Arm	Lenvatinib, Pembrolizumab
				Control Arm 1	Paclitaxel
				Control Arm 2	Doxorubicin
28	selinexor	KCP-330-023 (BOSTON)	NCT03110562	Treatment Arm	Selinexor, Bortezomib, Dexamethasone
				Control Arm	Bortezomib, Dexamethasone
29	polatuzumab vedotin-piiq	GO39942 (POLARIX)	NCT03274492	Treatment Arm	Polatuzumab Vedotin, Rituximab, Cyclophosphamide, Doxorubicin, Prednisone
				Control Arm	Rituximab, Cyclophosphamide, Doxorubicin, Prednisone
30	venetoclax	VIALE-A (M15-656)	NCT02993523	Treatment Arm	Venetoclax, Azacitidine
				Control Arm	Azacitidine
31	lorlatinib	CROWN (B7461006)	NCT03052608	Treatment Arm	Lorlatinib
				Control Arm	Crizotinib
32	pembrolizumab	KEYNOTE-204	NCT02684292	Treatment Arm	Pembrolizumab

				Control Arm	Brentuximab Vedotin
33	pembrolizumab	KEYNOTE-826	NCT03635567	Treatment Arm 1	Pembrolizumab, Paclitaxel, Cisplatin
				Treatment Arm 2	Pembrolizumab, Paclitaxel, Cisplatin, Bevacizumab
				Treatment Arm 3	Pembrolizumab, Paclitaxel, Carboplatin
				Treatment Arm 4	Pembrolizumab, Paclitaxel, Carboplatin, Bevacizumab
				Control Arm 1	Paclitaxel, Cisplatin
				Control Arm 2	Paclitaxel, Cisplatin, Bevacizumab
				Control Arm 3	Paclitaxel, Carboplatin
				Control Arm 4	Paclitaxel, Carboplatin, Bevacizumab
34	pemetrexed disodium	KEYNOTE-189	NCT02578680	Treatment Arm 1	Pemetrexed, Pembrolizumab, Cisplatin
				Treatment Arm 2	Pemetrexed, Pembrolizumab, Carboplatin
				Control Arm 1	Pemetrexed, Cisplatin
				Control Arm 2	Pemetrexed, Carboplatin
35	blinatumomab	AALL1331	NCT02003222	Treatment Arm	Blinatumomab, Cyclophosphamide, Cytarabine, Daunorubicin, Dexamethasone, Etoposide, Mercaptopurine, Methotrexate, Pegaspargase, Prednisone, Vincristine Sulfate IV, Leucovorin Calcium
				Control Arm	Cyclophosphamide, Cytarabine, Daunorubicin, Dexamethasone, Etoposide, Mercaptopurine, Methotrexate, Pegaspargase, Prednisone, Vincristine Sulfate IV, Leucovorin Calcium
36	bosutinib	BFORE	NCT02130557	Treatment Arm	Bosutinib
				Control Arm	Imatinib
37	pembrolizumab	KEYNOTE-177	NCT02563002	Treatment Arm	Pembrolizumab
				Control Arm 1	Leucovorin calcium, Fluorouracil, Oxaliplatin, Cetuximab

				Control Arm 2	Fluorouracil, Leucovorin calcium, Irinotecan
38	pembrolizumab	KEYNOTE-361	NCT02853305	Treatment Arm	Pembrolizumab
				Control Arm 1	Cisplatin, Gemcitabine
				Control Arm 2	Carboplatin, Gemcitabine
39	pembrolizumab	KEYNOTE-189	NCT02578680	Treatment Arm 1	Pembrolizumab, Pemetrexed, Cisplatin
				Treatment Arm 2	Pembrolizumab, Pemetrexed, Carboplatin
				Control Arm 1	Pemetrexed, Cisplatin
				Control Arm 2	Pemetrexed, Carboplatin
40	avelumab	JAVELIN Bladder 100	NCT02603432	Treatment Arm	Avelumab
				Control Arm	Best supportive care
41	brigatinib	ALTA 1L	NCT02737501	Treatment Arm	Brigatinib
				Control Arm	Crizotinib
42	avelumab	JAVELIN Merkel 200	NCT02155647	Treatment Arm	Avelumab
				Comparator Control	Pembrolizumab
43	pembrolizumab	KEYNOTE-204	NCT02684292	Treatment Arm	Pembrolizumab
				Control Arm	Brentuximab Vedotin
44	nivolumab	CHECKMATE-274	NCT02632409	Treatment Arm	Nivolumab
				Control Arm	Placebo
45	rucaparib	ARIEL3	NCT01968213	Treatment Arm	Rucaparib
				Control Arm	Placebo
46	pembrolizumab	KEYNOTE-048	NCT02358031	Treatment Arm	Pembrolizumab
				Control Arm 1	Cetuximab, Cisplatin, Fluorouracil
				Control Arm 2	Cetuximab, Carboplatin, Fluorouracil

47	venetoclax	MURANO (GO28667)	NCT02005471	Treatment Arm	Venetoclax, Rituximab
				Control Arm	Bendamustine, Rituximab
48	nivolumab	CHECKMATE-066	NCT01721772	Treatment Arm	Nivolumab
				Control Arm	Dacarbazine
49	alectinib	ALEX	NCT02075840	Treatment Arm	Alectinib
				Control Arm	Crizotinib
50	daratumumab	MMY3003 (POLLUX)	NCT02076009	Treatment Arm	Daratumumab, Lenalidomide, Dexamethasone
				Control Arm	Lenalidomide, Dexamethasone
51	osimertinib	AURA3	NCT02151981	Treatment Arm	Osimertinib
				Control Arm 1	Pemetrexed, Carboplatin
				Control Arm 2	Pemetrexed, Cisplatin
52	pembrolizumab	KEYNOTE-010	NCT01905657	Treatment Arm	Pembrolizumab
				Control Arm	Docetaxel
53	nivolumab	CHECKMATE-067	NCT01844505	Treatment Arm	Nivolumab, Ipilimumab
				Control Arm	Ipilimumab
54	palbociclib	PALOMA-2	NCT01740427	Treatment Arm	Palbociclib, Letrozole
				Control Arm	Letrozole
55	nivolumab	CHECKMATE-037	NCT01721746	Treatment Arm	Nivolumab
				Control Arm 1	Dacarbazine
				Control Arm 2	Carboplatin, Paclitaxel
56	olaparib	D0818C00002 (SOLO-2)	NCT01874353	Treatment Arm	Olaparib
				Control Arm	Placebo
57	blinatumomab	TOWER	NCT02013167	Treatment Arm	Blinatumomab

				Control 1	Fludarabine, Cytarabine, Filgrastim/Pegfilgrastim, Idarubicin
				Control 2	Cytarabine,asparaginase/PEG-asparaginase, vincristine, melphalan
				Control 3	Methotrexate, asparaginase/PEG-asparaginase, vincristine, melphalan
				Control 4	Clofarabine
58	pembrolizumab	KEYNOTE-006 (Ipilimumab-Naive Melanoma)	NCT01866319	Treatment Arm	Pembrolizumab
				Control Arm	Ipilimumab
59	ceritinib	ASCEND-4	NCT01828099	Treatment Arm	Ceritinib
				Control Arm 1	Pemetrexed, Cisplatin
				Control Arm 2	Pemetrexed, Carboplatin
60	ibrutinib	PCYC-1112-CA (RESONATE)	NCT01578707	Treatment Arm	ibrutinib
				Control Arm	Ofatumumab
61	dabrafenib	MEK115306 (COMBI-d)	NCT01584648	Treatment Arm	Dabrafenib, Trametinib
				Control Arm	Dabrafenib
62	trametinib	MEK115306 (COMBI-d)	NCT01584648	Treatment Arm	Trametinib, Dabrafenib
				Control Arm	Dabrafenib
63	pertuzumab	BO25126 (APHINITY)	NCT01358877	Treatment Arm 1	Pertuzumab + Trastuzumab + 5-Fluorouracil + Doxorubicin + Cyclophosphamide + Docetaxel
				Treatment Arm 2	Pertuzumab + Trastuzumab + Epirubicin + Cyclophosphamide + Paclitaxel
				Control Arm 1	Trastuzumab + 5-Fluorouracil + Doxorubicin + Cyclophosphamide + Docetaxel
				Control Arm 2	Trastuzumab + Epirubicin + Cyclophosphamide + Paclitaxel
64	pomalidomide	CC-4047-MM-003	NCT01311687	Treatment Arm	Pomalidomide, Dexamethasone
				Control Arm	Dexamethasone

65	ponatinib	PACE (AP24534-10-201)	NCT01207440	Treatment Arm	Ponatinib
			NCT00261846	Comparator Control	Bosutinib
66	Synribo (omacetaxine mepesuccinate)	CGX-635-CML-300 (no clinicaltrials.gov identifier)	PIVOTAL TRIAL: NCT00375219	Treatment Arm	Omacetaxine mepesuccinate
				Control Arm	Not available
67	carfilzomib	PX-171-009	NCT01080391	Treatment Arm	Carfilzomib, Lenalidomide, Dexamethasone
				Control Arm	Lenalidomide, Dexamethasone
68	selinexor	XPORT-DLBCL-030	NCT04442022	Treatment Arm	Selinexor, Rituximab, Gemcitabine, Dexamethasone, Cisplatin
				Control Arm	Rituximab, Gemcitabine, Dexamethasone, Cisplatin
69	tazemetostat	SYMPHONY-1/EZH-302	NCT04224493	Treatment Arm	Tazemetostat, Lenalidomide, Rituximab
				Control Arm from Stage 2	Lenalidomide, Rituximab
70	pembrolizumab	KEYNOTE-158	Pivotal Trial: NCT02628067	Treatment Arm	Pembrolizumab
				Comparator Control	Bevacizumab
71	lurbinectedin	ATLANTIS	NCT02566993	Treatment Arm	Lurbinectedin, Doxorubicin
				Control Arm 1	Cyclophosphamide, Doxorubicin
				Control Arm 2	Vincristine, Topotecan
72	rucaparib	TRITON3	NCT02975934	Treatment Arm	Rucaparib
				Control Arm 1	Abiraterone Acetate
				Control Arm 2	Enzalutamide
				Control Arm 3	Docetaxel
73	pomalidomide	Pivotal Trial: Study 12-C-0047	Pivotal Trial: NCT01495598	Treatment Arm	Pomalidomide
			Comparator trial: NCT00003350	Comparator Control	Paclitaxel
74	pomalidomide		Pivotal Trial: NCT01495598	Treatment Arm	Pomalidomide

		Pivotal Trial: Study 12-C-0047	Comparator trial: NCT00003350	Comparator Control	Paclitaxel
75	selpercatinib	LIBRETTO-531	NCT04211337	Treatment Arm	Selpercatinib
				Control Arm 1	Cabozantinib
				Control Arm 2	Vandetanib
76	selpercatinib	LIBRETTO-001 (Pivotal Trial)	NCT03157128 (Pivotal Trial)	Treatment Arm	Selpercatinib
			Comparator trial: NCT04211337	Comparator Control 1	Cabozantinib
			Comparator trial: NCT04211337	Comparator Control 2	Vandetanib
77	pemigatinib	FIGHT-302	NCT03656536	Treatment Arm	Pemigatinib
				Control Arm	Gemcitabine, Cisplatin
78	ipilimumab	CheckMate-9DW	NCT04039607	Treatment Arm	Ipilimumab, Nivolumab
				Control Arm 1	Sorafenib
				Control Arm 2	Lenvatinib
79	nivolumab	CheckMate-9DW	NCT04039607	Treatment Arm	Nivolumab, Ipilimumab
				Control Arm 1	Sorafenib
				Control Arm 2	Lenvatinib
80	tazemetostat	Phase 1b/3 trial	NCT04204941	Treatment Arm	Tazemetostat, Doxorubicin
				Control Arm	Doxorubicin
81	zanubrutinib	BGB-3111-306 (Brukinsa for untreated MCL)	NCT04002297	Treatment Arm	Zanubrutinib, Rituximab
				Control Arm	Bendamustine, Rituximab
82	entrectinib	ALKA, STARTRK-1, STARTRK-2	ALKA-372-001 NCT02097810 NCT02568267	Treatment Arm	Entrectinib
			Comparator trial: NCT02576431	Comparator Control	Larotrectinib

83	larotrectinib	LOXO-TRK-14001<br>SCOUT<br>NAVIGATE	NCT02122913, NCT02637687, NCT02576431	Treatment Arm	Larotrectinib
				Comparator Control	Entrectinib
84	nivolumab	CheckMate -8HW	NCT04008030	Treatment Arm	Nivolumab, Ipilimumab
				Control Arm	mFOLFOX6 and Cetuximab
				Control Arm	FOLFIRI alone
85	ipilimumab	CheckMate -8HW	NCT04008030	Treatment Arm	Nivolumab, Ipilimumab
				Control Arm	mFOLFOX6 and Cetuximab
				Control Arm	FOLFIRI alone
86	acalabrutinib	ECHO/ACE-LY-308 (acalabrutinib for naïve MCL)	NCT02972840	Treatment Arm	Acalabrutinib, Bendamustine, Rituximab
				Control Arm	Bendamustine, Rituximab
87	nivolumab	CheckMate -8HW	NCT04008030	Treatment Arm	Nivolumab
				Control Arm	mFOLFOX6 and Cetuximab
				Control Arm	FOLFIRI alone
88	Opdivo (nivolumab)	SWOG S1826	NCT03907488	Treatment Arm 1	Nivolumab, Doxorubicin, Vinblastine Sulfate, Dacarbazine, Filgrastim
				Treatment Arm 2	Nivolumab, Doxorubicin, Vinblastine Sulfate, Dacarbazine, Pegfilgrastim
				Treatment Arm 3	Nivolumab, Doxorubicin, Vinblastine Sulfate, Dacarbazine
				Control Arm 1	Brentuximab Vedotin, Doxorubicin, Vinblastine Sulfate, Dacarbazine, Filgrastim
				Control Arm 2	Brentuximab Vedotin, Doxorubicin, Vinblastine Sulfate, Dacarbazine, Pegfilgrastim
				Control Arm 3	Brentuximab Vedotin, Doxorubicin, Vinblastine Sulfate, Dacarbazine
89	nivolumab	SWOG S1826	NCT03907488	Treatment Arm 1	Nivolumab, Doxorubicin, Vinblastine Sulfate, Dacarbazine, Filgrastim
				Treatment Arm 2	Nivolumab, Doxorubicin, Vinblastine Sulfate, Dacarbazine, Pegfilgrastim

				Treatment Arm 3	Nivolumab, Doxorubicin, Vinblastine Sulfate, Dacarbazine
				Control Arm 1	Brentuximab Vedotin, Doxorubicin, Vinblastine Sulfate, Dacarbazine, Filgrastim
				Control Arm 2	Brentuximab Vedotin, Doxorubicin, Vinblastine Sulfate, Dacarbazine, Pegfilgrastim
				Control Arm 3	Brentuximab Vedotin, Doxorubicin, Vinblastine Sulfate, Dacarbazine
90	belinostat	CRESCENDO	NCT06072131	Treatment Arm	Belinostat Injection, Cyclophosphamide, Doxorubicin, Vincristine, Prednisone
				Control Arm	Cyclophosphamide, Doxorubicin, Vincristine, Prednisone

CHEERS 2022 Checklist

Topic	No.	Item	Location where item is reported
**Title**			
	1	Identify the study as an economic evaluation and specify the interventions being compared.	Page 1
**Abstract**			
	2	Provide a structured summary that highlights context, key methods, results, and alternative analyses.	Page 2
**Introduction**			
**Background and objectives**	3	Give the context for the study, the study question, and its practical relevance for decision making in policy or practice.	Pages 4-5
**Methods**			
**Health economic analysis plan**	4	Indicate whether a health economic analysis plan was developed and where available.	Not applicable
**Study population**	5	Describe characteristics of the study population (such as age range, demographics, socioeconomic, or clinical characteristics).	Page 6
**Setting and location**	6	Provide relevant contextual information that may influence findings.	Page 8
**Comparators**	7	Describe the interventions or strategies being compared and why chosen.	Page 8
**Perspective**	8	State the perspective(s) adopted by the study and why chosen.	Page 8
**Time horizon**	9	State the time horizon for the study and why appropriate.	Page 8
**Discount rate**	10	Report the discount rate(s) and reason chosen.	Page 8
**Selection of outcomes**	11	Describe what outcomes were used as the measure(s) of benefit(s) and harm(s).	Page 8
**Measurement of outcomes**	12	Describe how outcomes used to capture benefit(s) and harm(s) were measured.	Page 7
**Valuation of outcomes**	13	Describe the population and methods used to measure and value outcomes.	Not applicable
**Measurement and valuation of resources and costs**	14	Describe how costs were valued.	Page 8
**Currency, price date, and conversion**	15	Report the dates of the estimated resource quantities and unit costs, plus the currency and year of conversion.	Page 8

Topic	No.	Item	Location where item is reported
**Rationale and description of model**	16	If modelling is used, describe in detail and why used. Report if the model is publicly available and where it can be accessed.	Page 8
**Analytics and assumptions**	17	Describe any methods for analysing or statistically transforming data, any extrapolation methods, and approaches for validating any model used.	Page 8
**Characterising heterogeneity**	18	Describe any methods used for estimating how the results of the study vary for subgroups.	Page 8
**Characterising distributional effects**	19	Describe how impacts are distributed across different individuals or adjustments made to reflect priority populations.	Not applicable
**Characterising uncertainty**	20	Describe methods to characterise any sources of uncertainty in the analysis.	Page 8
**Approach to engagement with patients and others affected by the study**	21	Describe any approaches to engage patients or service recipients, the general public, communities, or stakeholders (such as clinicians or payers) in the design of the study.	Page 9
**Results**			
**Study parameters**	22	Report all analytic inputs (such as values, ranges, references) including uncertainty or distributional assumptions.	Supplementary appendix
**Summary of main results**	23	Report the mean values for the main categories of costs and outcomes of interest and summarise them in the most appropriate overall measure.	Page 11
**Effect of uncertainty**	24	Describe how uncertainty about analytic judgments, inputs, or projections affect findings. Report the effect of choice of discount rate and time horizon, if applicable.	Page 11
**Effect of engagement with patients and others affected by the study**	25	Report on any difference patient/service recipient, general public, community, or stakeholder involvement made to the approach or findings of the study	Not applicable
**Discussion**			
**Study findings, limitations, generalisability, and current knowledge**	26	Report key findings, limitations, ethical or equity considerations not captured, and how these could affect patients, policy, or practice.	Page 12
**Other relevant information**			
**Source of funding**	27	Describe how the study was funded and any role of the funder in the identification, design, conduct, and reporting of the analysis	Page 15
**Conflicts of interest**	28	Report authors conflicts of interest according to journal or International Committee of Medical Journal Editors requirements.	Page 15

From: Husereau D, Drummond M, Augustovski F, de Bekker-Grob E, Briggs A H, Carswell C et al. Consolidated Health Economic Evaluation Reporting Standards 2022 (CHEERS 2022) statement: updated reporting guidance for health economic evaluations. *BMJ.* 2022;376:e067975.
