# Supplementary material for: Costs and benefits of early access to new cancer drugs through the US Food and Drug Administration’s accelerated approval pathway: retrospective observational study and economic evaluation
Source: BMJ Med. 2025 Dec 16;4(1):e001934. doi: 10.1136/bmjmed-2025-001934 (PMC12718571; doi:10.1136/bmjmed-2025-001934)
Supplement: Supplementary file 2 [file bmjmed-2025-001934.reviewer_comments.pdf]

PEER REVIEW HISTORY

BMJ Medicine publishes all reviews undertaken for accepted manuscripts. Reviewers are asked to complete a checklist review form and are provided with free text boxes to elaborate on their assessment. These free text comments are reproduced below.

This paper was submitted to another journal from BMJ but declined for publication following peer review. The authors addressed the reviewers' comments and submitted the revised paper to BMJ Medicine. The paper was subsequently accepted for publication at BMJ Medicine.

ARTICLE DETAILS

**TITLE (PROVISIONAL)**	Costs and Benefits of Early Access to New Cancer Drugs through the U.S. Food and Drug Administration Accelerated Approval Pathway
**AUTHORS**	Naci, Huseyin; Shahzad, Mahnum; Murphy, Peter; Zhang, Yichen; Costa, Rebecca; Ross, Joseph; Wagner, Anita

VERSION 1 - REVIEW

**REVIEWER NAME**	Bryan, Michael
**REVIEWER AFFILIATION**	University of Oxford
**REVIEWER CONFLICT OF INTEREST**	None
**DATE REVIEW RETURNED**	29-Jun-2025

**GENERAL COMMENTS**	This paper evaluates whether the U.S. FDA's accelerated approval pathway for cancer drugs delivers meaningful survival benefits relative to its costs. Analysing Medicare claims and FDA data, the authors find that many drugs granted accelerated approval fail to show survival advantages, yet remain on the market, accrue high costs, and are often converted to full approval based on surrogate endpoints. The study highlights a system that prioritises rapid access over rigorous evidence, with patients exposed to expensive treatments that may not extend life, and billions in Medicare spending directed towards therapies of uncertain value.<br><br>To address these concerns, the authors propose strengthening post -approval trial requirements, shifting toward survival-based endpoints, linking drug pricing to demonstrated clinical benefit, and improving transparency with doctors and patients. At a time of debate about accelerated approvals, this paper makes a timely and important contribution, offering an analysis that balances the need for early access with the ethical obligation for evidence-based care.<br><br>I suggest improvements mainly in clarifying the framing of policy trade-offs, transparency about modelling assumptions, improving the discussion of opportunity costs and patient-centred outcomes, and slightly refining language for clarity and precision.<br>-<br><br>Major Comments:<br><br>Abstract<br>• Reframe the objective more directly around patients and costs

(e.g., “It remains unclear whether the FDA’s accelerated approval pathway for cancer drugs provides meaningful survival benefits relative to its costs”) would better engage readers and more clearly define the policy problem.
Introduction
• Lines 20-30: The second paragraph lists multiple important concerns (inconsistent confirmatory trials, delays in withdrawal, surrogate endpoint reliance) but compresses them into a single block. This risks overwhelming the reader. Consider either splitting into two focused paragraphs or adding clearer transitions between ideas to improve logical flow.
• Lines 50-51: The current explanation of the counterfactual (“inherent to this trade-off is the counterfactual…”) is abstract and could be difficult for non-specialist readers to follow. Suggest rephrasing in simpler language to make it more accessible.

Methods
• Lines 6-7: The Methods could more clearly explain how comparator treatments were selected when no randomised control group was available (e.g., best available therapy at the time). Although the Limitations touch on this, clearer upfront description would strengthen understanding of a key source of potential bias in survival and spending estimates.
• Lines 35-38, 45-46: The modelling assumes a constant hazard over time by fitting exponential curves to median survival data. This could misestimate survival gains for cancers with non-constant risk patterns (e.g., early mortality vs late separation). Acknowledging this limitation would help readers interpret the precision and potential biases in the results.
• Line 37: The Methods note that the model used the latest available survival data, even when findings were not statistically significant. Clarifying that this was a deliberate modelling choice (to reflect real-world uncertainty) would strengthen this.
• Lines 41-43: The reference to “still ongoing as of 30 November 2024” could confuse readers since the main patient follow-up ends in 2022. If 2024 refers only to administrative tracking of confirmatory trial status, this should be clearly explained.
• The sensitivity analyses are thorough but technically dense. It would help to introduce this section by highlighting which assumptions were expected to have the largest potential impact on results (e.g., comparator assumptions, allocation methods, survival modelling, duration of early access).

Results
• Lines 8-10, 31-34: The description of the 90 indications is clear, but the list of cancer types feels mechanical. Briefly explain why these cancers are important (e.g., high-burden, high-cost cancers in Medicare).
• Line 22: The Results refer to 11 indications with “unknown” survival benefit. It is unclear whether this reflects immature, ongoing, or methodologically limited data. A short explanation would clarify the nature of this uncertainty and strengthen the findings.
• Lines 39-41: The finding that more patients received treatments for indications later withdrawn could be misinterpreted as evidence of declining approval standards. It would strengthen the Results to clarify that this trend may instead reflect delays in the completion of confirmatory trials and subsequent regulatory action, rather than a deterioration in the initial quality of accelerated approvals.
• Lines 52-53: The Results report survival gains, but it is not made sufficiently clear that these estimates are modelled from trial data

	applied to Medicare utilisation, rather than directly measured real-world survival outcomes. Clarifying this distinction would prevent misinterpretation.<br>• Line 39: The phrase "spending per life-year lost" could confuse readers, as it is not a standard metric. Brief clarification is needed to explain that this reflects substantial Medicare spending despite no observed survival benefit.<br>• Lines 44-48: While sensitivity analyses are described, the authors should briefly highlight that principal findings remained stable across a range of assumptions, to strengthen reader confidence in the robustness of the results.<br><br>Discussion<br>• Line 5: The Discussion does not fully highlight the real-world significance for patients and policymakers. The phrasing could be strengthened to emphasise how early access via accelerated approval translated into real survival gains and additional costs for Medicare beneficiaries, not just abstract metrics.<br>• Lines 17-19: While the section summarises results well, it would benefit from 1-2 sentences clearly framing the key policy trade-off: Medicare incurred substantial costs, but survival returns were uneven, raising questions about evidence standards and resource allocation.<br>• Line 36: The manuscript could more explicitly discuss the broader opportunity costs: that inefficient spending on low-value therapies displaces potential investment in prevention, early detection, or curative innovation, not just representing financial "waste."<br>• The Discussion could acknowledge that Medicare beneficiaries are older and more medically complex, and may face disproportionate harms (toxicity, reduced quality of life) from therapies offering little or no survival benefit.<br>• While the authors mention that only survival was assessed, they should more provide additional context for important outcomes such as quality of life (QoL), symptom control, and patient-reported outcomes. Demonstrating from the literature, for example, that QoL improvements are rare among accelerated approvals would strengthen their argument rather than weaken it.<br>• The Discussion currently frames early access largely as a systemic failure, but it could be more balanced by acknowledging that for many patients facing life-threatening cancers, accepting uncertainty may be rational. Strengthening communication standards (e.g., similar to the European Medicines Agency's conditional approval framework requiring explicit disclosure of uncertainty) could be proposed as a complementary reform.<br>• The critique of surrogate endpoints could be nuanced by noting that in settings like adjuvant immunotherapy or cancer vaccines, scientifically plausible immune-based surrogates (e.g., T-cell responses) may offer valid interim markers. Acknowledging that not all surrogate use is equivalent would reflect current scientific realities.<br>• Regulatory frameworks increasingly accept well-designed real-world evidence (RWE) to supplement trial data. The Limitations could be strengthened by acknowledging the growing use of RWE (e.g., synthetic control arms) and suggesting that future accelerated approvals require structured RWE plans with predefined validation timelines.<br>• Excessively tightening accelerated approval could inadvertently suppress innovation, particularly for small biotech firms developing first-in-class therapies. Acknowledging this would allow the paper to advocate for a more nuanced reform approach: tightening standards

	for marginal therapies while preserving pathways for responsible innovation backed by strong biological plausibility and time-bound validation.<br><br>Conclusion<br>• While accurately summarising usage numbers, costs, and survival outcomes, it reads somewhat dry and administrative. The authors could briefly emphasise that these figures represent real patients often exposed to high-cost treatments with limited or unproven survival benefit. Highlighting the policy concern — that substantial Medicare spending occurred before strong clinical benefit was confirmed — would provide a more impactful close.<br>• In addition, the Conclusion should more directly frame the underlying trade-off between early access to therapies and the risks of clinical uncertainty, tying the findings back to broader regulatory and health system implications.<br><br>Minor Comments:<br><br>Title:<br>• The title could be sharpened for focus. Currently, it combines many concepts (costs, benefits, Medicare, cancer drugs, accelerated approval, FDA), which risks diluting the main message.<br>• The phrase “access to” could imply that beneficiaries actively choose treatments, whereas access is system-mediated (via FDA approval, prescribing, and coverage). A minor rewording could clarify this distinction.<br><br>Abstract:<br>• Line 15–16: Clarify the endpoint description for approvals: revise to “…conversion to regular approval, withdrawal, or still under accelerated approval as of 31 December 2020.”<br>• Line 24: Suggest rewording for clarity: “…were followed for survival and outcomes until 31 December 2022.”<br>• Line 32: Typographical correction: “…resulted in an estimated 76,164 life-years gained” (insert hyphen).<br>• Line 36: Grammatical correction: “costs… were $20.1 billion” (costs = plural).<br>• Line 49: Typographical correction: “…most beneficiaries deriving no or only small survival gains.” (Note: similar phrasing error appears multiple times; recommend a global search and correction.)<br>• Line 48–51: Sentence structure could be improved for clarity: split into two sentences — “…despite most beneficiaries deriving no or only small survival gains from these treatments. Only three drugs accounted for over two-thirds of the total life-year gains among beneficiaries.”<br><br>Introductions:<br>• Lines 36–37: Wording improvement: “drugs that show improvements on surrogate endpoints often do not lead to real survival gains or improvements in how patients feel or function.” This avoids the weakened phrasing caused by the double qualifier.<br>• Line 50: Typographical correction: “substantial life-year gains” (add hyphen).<br><br>Methods<br>• Line 15: Replace "collected" with "extracted" to more accurately describe use of existing Medicare data.<br>• Line 17-20: Break a long sentence into two for clarity when describing survival gains and spending differences between groups.

• Line 22: Small phrasing improvement: "…the first year for which Medicare spending data were available."
• Line 25: Typographical correction: "conversial" → "conversion".
• Lines 24–26: Replace "i.e." with "meaning" for more natural flow.
• Lines 28–29: Specify which aspects were reported according to CHEERS (e.g., model structure, costing methods).
• Line 46: Minor wording edit: "We sought to estimate…" → "We estimated…"
• Lines 7–11: Smooth out mechanical flow caused by multiple "We…" sentences; introduce linking phrases.
• Lines 19–20: Reword a complex sentence about attribution of indications to improve readability and flow.
• Lines 38–42: Clarify whether identification of confirmatory trial-linked publications was systematic or manual.
• Lines 56–57: Minor rewording: "…categorised each indication based on whether a statistically significant overall survival benefit was demonstrated…"
• Line 19: Correct grammar: "spending…was available" (not "were").
• Lines 29–31: Suggest softening blunt phrasing from "alive and dead" to "alive and deceased".
• Lines 35–36 and 45–46: Recommend explicitly acknowledging the simplification inherent in the exponential curve fitting method.
• Line 37: Suggest rephrasing to clarify that including non-significant survival results was a deliberate modelling decision.
• Lines 46–47: Recommend briefly explaining why the model did not discount future life-years and costs, as discounting is standard in cost-effectiveness analyses.
• Lines 50–51: Minor stylistic improvement: "we ran 10,000 Monte Carlo simulation iterations for each drug indication to capture uncertainty."

Results
• Line 7: Typographical clarification: reword to "excluding 7 indications, corresponding to 5 drugs, for which Medicare spending data were unavailable."
• Lines 19–22: Reword for precision: "while the remaining 26 indications were converted to regular approval based on surrogate endpoints, despite lacking evidence of overall survival benefit or having unknown benefit."
• Lines 25–26: Simplify phrasing: "Randomised controlled trials found no evidence of overall survival benefit for 30 indications (33.3%), including 11 that were later converted to regular approval."
• Lines 31–32: Strengthen framing: "178,708 beneficiaries received drugs through early access via the FDA's accelerated approval pathway, often before benefits were confirmed."
• Line 39: Typographical correction: "18.0%%" → "18.0%."
• Lines 36–39: Reword for clarity: "142,760 beneficiaries for indications eventually converted to regular approval, 5,574 for indications still under accelerated approval, and 30,374 for indications later withdrawn."
• Lines 46–48: Recommend directly quantifying the difference in beneficiaries receiving drugs with vs. without survival benefit (e.g., "about 17,000 vs. 9,000 in 2020") to make the comparison more tangible.
• Line 53: Typographical correction: "life-years gained" (insert hyphen).
• Lines 7–8: Clarify phrasing: "resulted in no observed life-year gains in survival outcomes."
• Line 14: Grammar correction: "costs…were $20.1 billion."
• Line 23: Recommend clarifying that many indications converted to

	regular approval did so without strong survival evidence.<br><br>Discussion:<br>• Line 5: Wording improvement for impact: "We quantified the life-year gains and costs of use of new cancer drugs for indications granted FDA accelerated approval" → "We evaluated how early access to new cancer drugs through the FDA's accelerated approval pathway translated into real survival gains and additional costs for Medicare beneficiaries."<br>• Line 9: Minor phrasing edit: "…at an additional cost to the Medicare program…" → "…at an added cost of approximately $20.1 billion to Medicare."<br>• Lines 8–10: Reword for better nuance: "…treatments to reach patients sooner, though often with greater uncertainty than under traditional approval processes."<br>• Lines 17–19: Improve phrasing for clarity: "…many conversions to regular approval occurred without evidence of improved survival."<br>• Lines 17–19: Minor edit for sharper phrasing: "…would better align Medicare spending with treatments that offer proven clinical benefit."<br>• Lines 31–36: Suggest briefly referencing an example of value-based pricing or outcomes-based reimbursement initiatives to strengthen proposals.<br>• Line 42: Improve clarity: "…which can lead to continued patient exposure to ineffective treatments and undermine trust in clinical recommendations."<br>• Lines 44–53: The international comparison feels slightly rushed; recommend adding a summarising sentence to emphasise that without harmonised standards, uncertainty about benefit may persist globally.<br>• Lines 4–5: Wording improvement to avoid self-promotion: rephrase "makes an important and novel contribution" to "provides a new, integrated analysis of survival benefits, spending, and approval outcomes for cancer drugs under the accelerated approval pathway."<br>• Lines 9–10: Softer phrasing to avoid overstating: "extends previous analyses to provide a broader evaluation of…" instead of "comprehensively evaluate…"<br><br>Conclusion:<br>• Lines 50–51: Wording improvement: "…with just three drug indications responsible for over two-thirds of the total life-year gains" for smoother and more active phrasing.<br><br>Overall, the authors have produced a clear and timely analysis of an important regulatory and policy issue. This paper is a strong contribution and merits acceptance following minor revision.

**REVIEWER NAME**	Glatz, Toivo
**REVIEWER AFFILIATION**	Charité - Universitätsmedizin Berlin
**REVIEWER CONFLICT OF INTEREST**	Toivo Glatz received funding for the Peer Review training initiative Peerspectives from the Volkswagen Foundation.
**DATE REVIEW RETURNED**	29-Jun-2025

**GENERAL COMMENTS**	In this study, Naci and colleagues compared the gains in overall survival against the incremental costs of new cancer drugs that received early access due to an accelerated approval by the FDA. This was specifically investigated in the U.S. Medicare setting, where accelerated approvals mark a growing share of the drug

	expenses. This study raises the question of whether it is sufficient to assess clinical effectiveness based on surrogate parameters. While the study topic is certainly highly relevant and the inclusion of all 90 cancer drugs in accelerated approval makes it comprehensive, the methodological approach often seems simplistic and its reporting is inadequate. The following comments should be considered to improve the main messages of the manuscript:<br><br>Major comments:<br>1) Overall lack of clarity and reporting regarding the rationale for the choice of methods.<br><br>a) Synthesis of the data from the respective RCTs (several trial identifiers) for the 90 drugs and the extrapolation thereof was not described; it seems that 90 meta-analyses may have been conducted, but were not reported in the manuscript.<br><br>b) In line with the BMJ open code policy, the statistical code for the analysis should be provided to aid interpretation and reproducibility.<br><br>c) The research question is not clearly stated (p. 2, l. 5-9; p. 5, l. 16-18) and the main conclusion remains vague throughout the manuscript (p. 2, l. 42-44; p.3, l. 15-16; p. 14, l. 43).<br><br>d) The comparison groups are not clearly explained, especially for the cost data from Medicare.<br><br>e) The authors should add more explanation to the Medicare system & Medicare Advantage in the introduction section, as this may not be entirely clear to an international audience.<br><br>f) Sufficient reasoning for the executed sensitivity analysis are not provided and the results in Fig. 7 are not informative for the underlying research question. Other sensitivity analyses are recommended (see below, e.g., discount rates, trial type, drug prices, effect size).<br><br>2) Some essential components of the cost-effectiveness analysis are missing, which very likely underestimates costs and overestimates effectiveness, therefore overestimating overall the true cost-effectiveness.<br><br>a) Only life-years gained, but no QALYs were considered. Even if such data is not available, the authors could provide a range of common utilities for novel cancer drugs based on the literature. Depending on side-effects, cost per QALY may be 20-70% higher than the cost per LYG reported. The authors also don't report a reference for the 150.000$ per LYG gained as cost-effectiveness threshold and I suspect this was set for QALYs and not LYG.<br><br>b) The fit of the exponential function within the two-stage model seems overly simplistic. Some trials only provide data for 1-2 years, but it seems like survival is extrapolated up until 10 years. I would suggest a sensitivity analysis limiting survival estimation to the actual trial durations. The exponential function can also not accommodate any plateaus or other dynamics in the tail of the distribution. I would suggest the authors report sensitivity analyses with other, more flexible parametric alternatives.<br><br>c) No discounting was applied and no reasoning for this decision

	has been provided. This will again underestimate the true costs compared to a standard cost-effectiveness approach.<br><br>d) Information as requested by the CHEERS Checklist is not fully provided (e.g. No. 1, No. 5, No. 10).<br><br>3) The algorithm extrapolating from the selective Medicare Advantage population to the broader Medicare population is not explained at all and the provided references do not explain it in detail either. While the authors claim that no differences in cancer prevalence were found between Medicare Advantage vs. traditional Medicare this does not automatically translate to no differences in prescription and filling/billing of treatments investigated here. Contrary to the authors claims there are also systematic differences between these groups (e.g., in geographic distribution, proportion of non-white individuals, education, income, etc.) which indicates a clear selection bias (also see https://www.kff.org/report-section/disparities-in-health-measures-by-race-and-ethnicity-among-beneficiaries-in-medicare-advantage-report/).<br><br>a) It would help if the authors provide some baseline characteristics of the study population, e.g. a descriptive analysis of the Medicare Advantage population.<br><br>b) The Medicare Advantage population of a single commercial health insurer is likely not generalizable to the entire Medicare population. This should be discussed in the Limitation section.<br><br>Minor comments:<br><br>Abstract:<br><br>4) General: The abstract does not clarify the methods and analyses done to evaluate the cost-effectiveness analysis (e.g., interventions are not clearly stated).<br><br>5) Participants: (1) Please add more information about the study population (+65 years) as this may not be clear to readers outside the US. (2) p. 2, l. 15: I recommend writing “through to” instead of “through” for clarity.<br><br>What this study adds:<br><br>6) P. 3, l. 20: How do the authors conclude that the additional spending was “substantial”? Please include the WTP threshold that was used to interpret the findings and explain whether the threshold is commonly used in the context of overall survival or rather applied to other endpoints (like QoL).<br><br>Introduction:<br><br>7) P. 4, l. 47: This is the same sentence already mentioned in the “participants section” of the abstract and is not clearly phrased.<br><br>8) P. 4, l. 46: This sentence is not clearly phrased. Maybe it should read "some cancer drugs" or "most cancer drugs"?<br><br>9) P. 5, l. 13: The authors could include some proportions for the growing share of increase in cancer drugs.

	Methods:<br><br>10) Please provide information on the software used for the calculations and modeling.<br><br>11) How do drugs/indications based on single-arm trials enter the results? How are the life-years gained estimated in the absence of a control group?<br><br>12) How was the availability of OS benefit classified, e.g. Appendix Table 3: for drug ID 1, the OS benefit was classified as "no", but the HR CI does not contain 1 and indicates a protective effect of the intervention.<br><br>13) P. 6, l. 19: The people receiving control treatments are not listed as participants, but their data seems to have been used, please explain.<br><br>14) P. 6, l. 23-24: Given the different follow-up periods starting in 2012 and ending in 2020, 2022 and 2024, respectively, the authors should consider creating a timeline figure that shows the study design to help interpretation.<br><br>15) P. 6, l. 29: Please add a reference to the appendix for the CHEERS Checklist.<br><br>Results:<br><br>16) Please mention the results of the probabilistic sensitivity analysis (apart from Fig. 7).<br><br>17) Figure 7: Please describe in the Figure or caption how the base case and the scenarios are defined.<br><br>18) Figure 4: The Figure is well presented, but would benefit from CIs. Please also consider adding CIs to the Figures 1, 2 & 7.<br><br>19) Please check whether some percentages have been calculated correctly, e.g., p. 10. l. 39/l.54, Table 1 (5.6% instead of 5.5% for multiple myeloma, 17.8% instead of 17.7% for accelerated approval status: withdrawn).<br><br>Discussion:<br><br>20) P. 12, l. 12: Please mention the cost-effectiveness threshold and provide a reference.<br><br>21) P. 12, l. 15: The authors should discuss the implications for pricing after drugs receive regular approval.<br><br>22) P. 12, l. 46-49: The authors report that the duration between accelerated approval and conversion/withdrawal was longer for indications without survival benefit compared to those with survival benefit (36 months vs. 16 months). Please add this information to the results and methods section.<br><br>Limitations:<br><br>23) The authors should discuss that accelerated approval drugs may show long-term benefits that are not fully captured at the

	chosen cut-off and that clinical trials provide limited data on that. That is why extrapolation is often used in models to account for this limitation.<br><br>COI STATEMENT:<br>This peer review was done under the Peer Review and Biomedical Editing Training Initiative (Peerspectives), which has been developed in cooperation with the BMJ. In total, six trainee reviewers and one experienced mentor reviewed this paper. All participants have agreed to the BMJ reviewer policies.<br><br>Trainee reviewers/PhD Students (alphabetical order): Antonia Bartz, Johannes Hengelbrock, Cana Kussmaul, Julia Mayer, Daniela Skalt, Levin Wiebelt<br><br>Mentor (takes responsibility for the review): Toivo Glatz<br><br>Potential competing interests: Toivo Glatz received funding for the Peer Review training initiative Peerspectives from the Volkswagen Foundation. The trainees report no conflicts of interest.

VERSION 1 – AUTHOR RESPONSE

Reviewer: 1

Comments:

This paper evaluates whether the U.S. FDA's accelerated approval pathway for cancer drugs delivers meaningful survival benefits relative to its costs. Analysing Medicare claims and FDA data, the authors find that many drugs granted accelerated approval fail to show survival advantages, yet remain on the market, accrue high costs, and are often converted to full approval based on surrogate endpoints. The study highlights a system that prioritises rapid access over rigorous evidence, with patients exposed to expensive treatments that may not extend life, and billions in Medicare spending directed towards therapies of uncertain value.

To address these concerns, the authors propose strengthening post-approval trial requirements, shifting toward survival-based endpoints, linking drug pricing to demonstrated clinical benefit, and improving transparency with doctors and patients. At a time of debate about accelerated approvals, this paper makes a timely and important contribution, offering an analysis that balances the need for early access with the ethical obligation for evidence-based care.

Authors: We thank the Reviewer for their positive assessment of our paper.

I suggest improvements mainly in clarifying the framing of policy trade-offs, transparency about modelling assumptions, improving the discussion of opportunity costs and patient-centred outcomes, and slightly refining language for clarity and precision.

Authors: We thank the Reviewer for these detailed comments. Addressing them has strengthened the paper.

Major Comments:

Abstract
• Reframe the objective more directly around patients and costs (e.g., “It remains unclear whether the FDA’s accelerated approval pathway for cancer drugs provides meaningful survival benefits relative to its costs”) would better engage readers and more clearly define the policy problem.

Authors: We have revised the objective to more directly emphasise patient outcomes and costs, as recommended. The revised objective now reads (**page 2, line 3**):

> *“**Objective:** To evaluate the survival gains and additional Medicare spending associated with early access to new cancer drugs granted accelerated approval by the U.S. Food and Drug Administration (FDA), compared with access following completion of confirmatory trials.”*

Introduction
• Lines 20-30: The second paragraph lists multiple important concerns (inconsistent confirmatory trials, delays in withdrawal, surrogate endpoint reliance) but compresses them into a single block. This risks overwhelming the reader. Consider either splitting into two focused paragraphs or adding clearer transitions between ideas to improve logical flow.

Authors: We thank the Reviewer for this suggestion. In response, we have revised the paragraph to improve clarity and logical flow. This paragraph now reads (**page 4, line 17**):

> *“To mitigate this uncertainty, the FDA requires post-marketing confirmatory trials to verify the clinical benefit of drugs granted accelerated approval. However, the implementation of this requirement has been inconsistent.[9–12] Between 1992 and 2020, nearly half of the drug indications that have received accelerated approval had not completed their confirmatory trials.[13] Delays in trial completion are common,[14] and some drugs remain on the market for extended periods without verified benefit. Even when confirmatory trials are completed, concerns persist regarding the quality of evidence used to establish clinical benefit. In more than half of accelerated approvals, surrogate endpoints were used not only to grant accelerated approval but also to establish benefit in confirmatory trials.[15] Delays have also occurred in regulatory action following the completion of confirmatory trials, including in cases where trials failed to demonstrate clinical benefit.[16]”*

• Lines 50-51: The current explanation of the counterfactual (“inherent to this trade-off is the counterfactual…”) is abstract and could be difficult for non-specialist readers to follow. Suggest rephrasing in simpler language to make it more accessible.

Authors: This sentence is longer included in our revised manuscript.

Methods
• Lines 6-7: The Methods could more clearly explain how comparator treatments were selected when no randomised control group was available (e.g., best available therapy at the time). Although the

Limitations touch on this, clearer upfront description would strengthen understanding of a key source of potential bias in survival and spending estimates.

Authors: We thank the Reviewer for this comment. In response, we have clarified our approach to comparator selection in several parts of the paper:

In the Methods Overview section, we now state (**page 6, line 11**):

> *"We determined whether cancer drug indications initially granted accelerated approval had demonstrated overall survival benefit compared to control treatments in clinical trials."*

We clarified our data extraction process (**page 8, line 12**):

> *"We extracted information on treatment and control groups, study design, primary endpoint(s), and study results from pivotal and confirmatory trials."*

We revised the description of our spending calculations to emphasise the role of comparator treatments (**page 8, line 30**):

> *"We calculated as additional spending the difference in Medicare spending between the treatment strategy including the accelerated approval drug and the standard of care strategy without the accelerated approval drug, as tested in the control arms in clinical trials."*

To address single-arm trials, we added (**page 8, line 36**):

> *"When only single-arm trials were available, we assumed no available treatments in our base-case analysis."*

We further clarified the following (**page 9, line 50**):

> *"In the base-case analysis, we assumed no relevant comparator treatment when a drug was evaluated in a single-arm trial. For a scenario analysis (spending scenario 1), we identified potentially relevant comparators by searching ClinicalTrials.gov using the drug name and its accelerated approval indication, and by reviewing FDA medical review documents available on Drugs@FDA for references to existing treatment alternatives."*

Finally, we added a supplementary table listing comparator treatments, indicating which were deemed relevant for drugs evaluated in single-arm trials.

• Lines 35-38, 45-46: The modelling assumes a constant hazard over time by fitting exponential curves to median survival data. This could misestimate survival gains for cancers with non-constant risk patterns (e.g., early mortality vs late separation). Acknowledging this limitation would help readers interpret the precision and potential biases in the results.

Authors: We have added further details about our modelling approach. As recommended, we have also acknowledged this issue explicitly in both the Methods and Limitations sections of the manuscript.

In the Methods section, we added (**page 9, line 8**):

> *"We estimated the proportion of patients alive over time by fitting an exponential curve to the median survival data, a pragmatic approach for long-term extrapolation in the absence of individual participant-level data. The exponential distribution is widely used in economic evaluations of cancer treatments. We assumed proportional hazards throughout the model to reflect a constant treatment effect on survival."*

In the Limitations section, we added (**page 16, line 5**):

> *"Second, our extrapolation of survival data relied on the exponential distribution. While this approach is standard in economic evaluations of treatments for advanced cancers, alternative parametric distributions may provide a better fit to the underlying data. However, applying such alternatives would require individual participant-level data and clinical validation, which were not available. In many cases, even Kaplan-Meier curves were not available, for example when the most recent overall survival data were extracted from clinicaltrials.gov. Additionally, the model adopted a limited time horizon, which may have led to underestimation of total life-years gained."*

• Line 37: The Methods note that the model used the latest available survival data, even when findings were not statistically significant. Clarifying that this was a deliberate modelling choice (to reflect real-world uncertainty) would strengthen this.

Authors: We thank the Reviewer for this suggestion. We have revised the statement to clarify our modelling approach, which now reads (**page 9, line 14**):

"To maximise use of available data, the model incorporated the most recent overall survival data, irrespective of the statistical significance of the findings."

• Lines 41-43: The reference to "still ongoing as of 30 November 2024" could confuse readers since the main patient follow-up ends in 2022. If 2024 refers only to administrative tracking of confirmatory trial status, this should be clearly explained.

Authors: We have clarified the statement by making the following revision (revised text underlined) (**page 6, line 20**):

"The study period began in 2012, with Medicare spending data available from that year. We considered accelerated approvals up to 31 December 2020 to ensure at least 4 years of follow-up data on overall survival from confirmatory trials (<u>up to 30 November 2024</u>)."

We have also added a timeline figure to the supplementary materials, as recommended by Reviewer 2.

• The sensitivity analyses are thorough but technically dense. It would help to introduce this section by highlighting which assumptions were expected to have the largest potential impact on results (e.g., comparator assumptions, allocation methods, survival modelling, duration of early access).

Authors: We have added an introductory paragraph to the scenario analyses section to highlight the assumptions expected to have the greatest impact on results.

This section reads (**page 9, line 39**):

> *"To assess the robustness of our findings to data limitations, we conducted several scenario analyses. These included: (1) varying comparator treatments for indications supported by single-arm trials or trials with multiple comparators, which affected estimated incremental spending; (2) reallocating drug utilisation across similar indications to account for limitations in claims data, which influenced both spending and survival outcomes; and (3) modelling survival benefits in the absence of overall survival data, which affected estimated life-years gained. We also varied the definitions of the duration of early access, which we hypothesised would have the greatest impact on results, as this assumption applied to all indications in our sample."*

Results

• Lines 8-10, 31-34: The description of the 90 indications is clear, but the list of cancer types feels mechanical. Briefly explain why these cancers are important (e.g., high-burden, high-cost cancers in Medicare).

Authors: The list of cancer types reflects the 90 indications that received FDA accelerated approval and therefore defines our study sample. This summary was not intended to convey the relative importance of these cancers to Medicare. We provide detailed Medicare spending figures by cancer type later in the Results section.

• Line 22: The Results refer to 11 indications with "unknown" survival benefit. It is unclear whether this reflects immature, ongoing, or methodologically limited data. A short explanation would clarify the nature of this uncertainty and strengthen the findings.

Authors: We thank the Reviewer for this suggestion. We have now added the following information to clarify (**page 11, line 17**):

> *"Of the 51 indications converted to regular approval, 25 had evidence of overall survival benefit, while the remaining 26 were converted to regular approval based on surrogate endpoints: 15 with no evidence of overall survival benefit and 11 with unknown overall survival benefit (6 due to single-arm trial designs and 5 due to immature survival data)."*

This information is also summarised in appendix tables 2 and 3, which are referenced at the beginning of the paragraph.

• Lines 39-41: The finding that more patients received treatments for indications later withdrawn could be misinterpreted as evidence of declining approval standards. It would strengthen the Results to clarify that this trend may instead reflect delays in the completion of confirmatory trials and subsequent regulatory action, rather than a deterioration in the initial quality of accelerated approvals.

Authors: We have addressed this point in the Discussion section. As we cannot be certain about the underlying cause of this pattern, we now acknowledge that the observed increase in patient exposure to drugs for indications later withdrawn may reflect several factors.

The new sentence reads (**page 13, line 17**):

> *"There was also a growing trend in the use of drugs for indications that were later withdrawn following negative confirmatory trials. This pattern might reflect declining evidence standards at the time of accelerated approval, delays in completing confirmatory trials, or delays in regulatory action."*

• Lines 52-53: The Results report survival gains, but it is not made sufficiently clear that these estimates are modelled from trial data applied to Medicare utilisation, rather than directly measured real-world survival outcomes. Clarifying this distinction would prevent misinterpretation.

Authors: We thank the Reviewer for this suggestion. We have revised the Results section to explicitly state that survival gains were modelled using trial data applied to Medicare utilisation. The revised sentence now reads (**page 11, line 48**):

> *"Our model, which applied trial data to Medicare utilisation, estimated that use of cancer drugs for indications that initially received accelerated approval resulted in 76,164 life years gained among Medicare beneficiaries."*

• Line 39: The phrase "spending per life-year lost" could confuse readers, as it is not a standard metric. Brief clarification is needed to explain that this reflects substantial Medicare spending despite no observed survival benefit.

Authors: We have revised this statement to improve clarity. The updated sentence now reads (**page 12, line 36**):

> *"Additional Medicare spending for lymphoma indications was not associated with life-year gains and potential loss of life."*

• Lines 44-48: While sensitivity analyses are described, the authors should briefly highlight that principal findings remained stable across a range of assumptions, to strengthen reader confidence in the robustness of the results.

Authors: We have added the following sentence to the Results section (**page 12, line 43**):

“Our findings remained stable across a range of assumptions.”

Discussion

• Line 5: The Discussion does not fully highlight the real-world significance for patients and policymakers. The phrasing could be strengthened to emphasise how early access via accelerated approval translated into real survival gains and additional costs for Medicare beneficiaries, not just abstract metrics.

Authors: We thank the Reviewer for this helpful suggestion. In response, we have revised the first paragraph of the Discussion section. The revised paragraph now reads (**page 13, line 3**):

> *“Between 2012 and 2020, early access to 90 cancer drug indications through the accelerated approval pathway enabled an estimated 178,708 Medicare beneficiaries to receive treatment sooner than they otherwise would have. This early access was associated with an estimated 76,164 additional life-years. However, these gains came at a substantial cost: approximately $20.1 billion in additional Medicare spending. On average, the additional cost to Medicare was $263,371 per life-year gained, significantly exceeding conventional cost-effectiveness thresholds in the U.S. healthcare system.”*

• Lines 17-19: While the section summarises results well, it would benefit from 1-2 sentences clearly framing the key policy trade-off: Medicare incurred substantial costs, but survival returns were uneven, raising questions about evidence standards and resource allocation.

Authors: We have added the following sentences to more clearly frame the key policy trade-off (**page 13, line 42**):

> *“Early access through accelerated approval yielded uneven survival returns, and Medicare incurred substantial costs for treatments that, in some cases, failed to deliver meaningful clinical benefit. The variation in survival gains across indications meant that substantial Medicare spending sometimes resulted in minimal or no life-year gains. For example, we estimated that 31,245 Medicare beneficiaries who accessed drugs for breast cancer indications between accelerated approval and conversion to regular approval, withdrawal, or December 2020, gained only 1,648 life-years by the end of 2022 at an additional cost of $7.4 billion, resulting in $4.5 million per life-year gained. These findings raise important questions about the value Medicare receives for its investment in cancer drug indications approved through the accelerated approval pathway and the evidence standards used to support continued coverage.”*

• Line 36: The manuscript could more explicitly discuss the broader opportunity costs: that inefficient spending on low-value therapies displaces potential investment in prevention, early detection, or curative innovation, not just representing financial "waste."

Authors: We thank the Reviewer for this point. Although we did not use the term "financial waste" in the paper, we have revised the Discussion section to more explicitly address the broader opportunity costs associated with Medicare spending on accelerated approval drugs. We now state (**page 14, line 1**):

> *"There are important health opportunity costs: resources allocated to treatments with uncertain or limited benefit may divert investment from other more effective interventions, or contribute to higher premiums and out-of-pocket costs for beneficiaries, which can have adverse health consequences."*

• The Discussion could acknowledge that Medicare beneficiaries are older and more medically complex, and may face disproportionate harms (toxicity, reduced quality of life) from therapies offering little or no survival benefit.

Authors: As recommended by the Reviewer, we have added the following sentence to the Discussion section (**page 16, line 28**):

> *"Medicare beneficiaries are older and have greater medical complexity, and may be disproportionately affected by treatment-related toxicities. "*

• While the authors mention that only survival was assessed, they should more provide additional context for important outcomes such as quality of life (QoL), symptom control, and patient-reported outcomes. Demonstrating from the literature, for example, that QoL improvements are rare among accelerated approvals would strengthen their argument rather than weaken it.

Authors: While we had previously noted that quality-of-life improvements are rare, we have ow expanded this point to provide additional context from the literature (**page 16, line 19**):

> *"Previous research indicates that quality-of-life improvements are rare." We have now expanded this to show the magnitude of this: "Improvement for quality-of-life endpoints was demonstrated in fewer than 5 percent of FDA cancer drug approvals between 2000 and 2020."*

• The Discussion currently frames early access largely as a systemic failure, but it could be more balanced by acknowledging that for many patients facing life-threatening cancers, accepting uncertainty may be rational. Strengthening communication standards (e.g., similar to the European Medicines Agency's conditional approval framework requiring explicit disclosure of uncertainty) could be proposed as a complementary reform.

Authors: We thank the Reviewer for this suggestion. We have addressed this comment in 2 ways:

Firstly, we have added balance to the Discussion section by acknowledging the heterogeneity in patient preferences. The revised paragraph now reads (**page 14, line 7**):

> *"The FDA's accelerated approval pathway was established to provide earlier access to treatments for patients with serious or life-threatening conditions, particularly when no alternative treatment options exist. It was designed to balance the potential benefits of earlier treatment access against the risks of clinical uncertainty associated with the use of surrogate endpoints.[4] This pathway allows treatments to become available sooner than they would under traditional approval processes.[26,56] While accelerated approval can lead to the use of therapies that later prove to be ineffective or only marginally effective, some patients with advanced cancers may choose to accept this uncetainty. Waiting for complete information on clinical benefits may harm patients due to delayed access to potentially effective treatments.[57] However, individuals vary in their preferences: many patients express a strong desire for certainty that a cancer drug will improve survival, and some are willing to wait longer for that evidence.[58] Balancing the risks of clinical uncertainty with the potential harms of delayed access remains central to the rationale for the accelerated approval pathway.[24,59]"*

Secondly, we have proposed an additional reform focused on improving communication standards. We added the following paragraph (**page 14, line 40**):

> *"The FDA could further strengthen the accelerated approval pathway by improving communication about the sources of uncertainty. The agency should explicitly disclose both the nature and magnitude of the uncertainty associated with surrogate endpoints and unverified clinical benefits. Currently, FDA-approved labeling for drugs, including those granted accelerated approval, often does not clearly communicate this uncertainty, limiting clinicians' and patients' ability to make fully informed treatment decisions.[51]"*

• The critique of surrogate endpoints could be nuanced by noting that in settings like adjuvant immunotherapy or cancer vaccines, scientifically plausible immune-based surrogates (e.g., T-cell responses) may offer valid interim markers. Acknowledging that not all surrogate use is equivalent would reflect current scientific realities.

Authors: We thank the Reviewer for this important point. In response, we have added information to the Discussion section about how surrogate endpoints that form the basis of regulatory decisions should be evaluated.

This section now reads (**page 14, line 30**):

> *"Therefore, surrogate endpoints used for accelerated approval should have a reliable and strong association with overall survival, ideally evaluated using meta-analysis methods.[52] Bivariate meta-analysis, which accounts for both the correlation between treatment effects*

and uncertainty, is the preferred approach.[53] In addition, confirmatory trials should measure overall survival whenever feasible, and avoid features such as treatment switching that may compromise survival assesment.[40]"

• Regulatory frameworks increasingly accept well-designed real-world evidence (RWE) to supplement trial data. The Limitations could be strengthened by acknowledging the growing use of RWE (e.g., synthetic control arms) and suggesting that future accelerated approvals require structured RWE plans with predefined validation timelines.

Authors: We agree that real-world evidence is increasingly used to support regulatory decision making. However, based on our earlier research, we have concerns about the reliability of real-world evidence in the absence of randomised controlled trials. In response, we have added the following statement to the Discussion section (**page 14, line 35**):

"Although there is growing interest in using real-world evidence for regulatory decision making, current claims and electronic health record systems are not sufficient,[53] and results from non-randomised studies may differ from those of randomised trials in unpredictable ways.[54]"

• Excessively tightening accelerated approval could inadvertently suppress innovation, particularly for small biotech firms developing first-in-class therapies. Acknowledging this would allow the paper to advocate for a more nuanced reform approach: tightening standards for marginal therapies while preserving pathways for responsible innovation backed by strong biological plausibility and time-bound validation.

Authors: We hope that the revisions made in response to the Reviewer's earlier comment have improved the balance of our discussion around the accelerated approval pathway. We have clarified that our recommendations such as requiring timely completion of confirmatory trials that measure overall survival are intended to strengthen the pathway.

Conclusion

• While accurately summarising usage numbers, costs, and survival outcomes, it reads somewhat dry and administrative. The authors could briefly emphasise that these figures represent real patients often exposed to high-cost treatments with limited or unproven survival benefit. Highlighting the policy concern — that substantial Medicare spending occurred before strong clinical benefit was confirmed — would provide a more impactful close.

• In addition, the Conclusion should more directly frame the underlying trade-off between early access to therapies and the risks of clinical uncertainty, tying the findings back to broader regulatory and health system implications.

Authors: We thank the Reviewer for this helpful suggestion. In response, we have revised the Conclusion section, which now reads (**page 16, line 36**):

"From 2012 to 2020, nearly 200,000 Medicare beneficiaries received new cancer drugs during the period between accelerated approval and either conversion to regular approval or withdrawal. More than half of this early access was for drug indications that ultimately did not demonstrate survival benefits, despite some receiving conversion to regular approval. Many patients were therefore exposed to therapies with limited or unverified clinical benefit. Access to new cancer drugs through the accelerated approval pathway accounted for an estimated $20 billion in additional Medicare spending. Yet most beneficiaries derived minimal or no survival gains, with only 3 drug indications accounting for more than two-thirds of the total life-years gained. These findings highlight the trade-off between earlier access to new therapies and substantial clinical uncertainty at significant cost. Strengthening evidence standards for timely completion of confirmatory trials may help ensure that early access delivers meaningful benefit to both patients, Medicare beneficiaries, and American taxpayers."

Minor Comments:

Title:
• The title could be sharpened for focus. Currently, it combines many concepts (costs, benefits, Medicare, cancer drugs, accelerated approval, FDA), which risks diluting the main message.

Authors: We have revised the title to read (**page 1, line 1**):

"Costs and Benefits of Early Access to New Cancer Drugs through the U.S. Food and Drug Administration Accelerated Approval Pathway"

• The phrase "access to" could imply that beneficiaries actively choose treatments, whereas access is system-mediated (via FDA approval, prescribing, and coverage). A minor rewording could clarify this distinction.

Authors: This terminology is widely used in the literature and policy discussions to describe the availability of treatments following regulatory approval. We would prefer to retain it as written.

Abstract:
• Line 15–16: Clarify the endpoint description for approvals: revise to "…conversion to regular approval, withdrawal, or still under accelerated approval as of 31 December 2020."

Authors: We thank the Reviewer for this suggestion. However, the proposed revision would not accurately describe the participants. We identified Medicare beneficiaries who used the cancer drug indications of interest during the period between accelerated approval and the earliest of the following: conversion to regular approval, withdrawal, or December 31, 2020.

To clarify, we have revised this section of the Abstract to read (**page 2, line 9**):

“Medicare beneficiaries who received new cancer drug indications from the time of FDA accelerated approval to conversion to regular approval, withdrawal, or 31 December 2020.”

• Line 24: Suggest rewording for clarity: “…were followed for survival and outcomes until 31 December 2022.”

Authors: We have made the suggested change (**page 2, line 17**):

“Beneficiaries who received accelerated approval indications were followed for survival and spending outcomes until 31 December 2022.”

• Line 32: Typographical correction: “…resulted in an estimated 76,164 life-years gained” (insert hyphen).

Authors: We thank the Reviewer noting this. We have made the change (**page 2, line 26**).

• Line 36: Grammatical correction: “costs… were $20.1 billion” (costs = plural).

Authors: We have made the correction.

• Line 49: Typographical correction: “…most beneficiaries deriving no or only small survival gains.” (Note: similar phrasing error appears multiple times; recommend a global search and correction.)

Authors: We have revised this statement throughout. It now reads (**page 3, line 12**):

“Most Medicare beneficiaries received treatments that yielded minimal or no overall survival gains.”

• Line 48–51: Sentence structure could be improved for clarity: split into two sentences — “…despite most beneficiaries deriving no or only small survival gains from these treatments. Only three drugs accounted for over two-thirds of the total life-year gains among beneficiaries.”

Authors: We have rewritten the Conclusions section of the Abstract, and these statements are no longer included.

Introductions:

• Lines 36–37: Wording improvement: “drugs that show improvements on surrogate endpoints often

do not lead to real survival gains or improvements in how patients feel or function.” This avoids the weakened phrasing caused by the double qualifier.

Authors: We thank the Reviewer for this suggestion. We have revised this statement to read (**page 4, line 33**):

> *“Therefore, drugs that show improvements on surrogate endpoints often do not lead to survival gains or improvements in how patients feel or function.”*

• Line 50: Typographical correction: “substantial life-year gains” (add hyphen).

Authors: This statement is longer included in the Introduction section.

Methods
• Line 15: Replace "collected" with "extracted" to more accurately describe use of existing Medicare data.

Authors: This change has been made.

• Line 17-20: Break a long sentence into two for clarity when describing survival gains and spending differences between groups.

Authors: We have revised this sentence to read (**page 6, line 15**):

> *“In the model, patients receiving treatment with accelerated approval drugs could accrue survival benefits. They could also incur additional Medicare spending compared to those receiving control treatments during the same period.”*

• Line 22: Small phrasing improvement: “…the first year for which Medicare spending data were available.”

Authors: We thank the Reviewer for this suggestion. We have now revised the sentence to read (**page 6, line 20**):

> *“The study period began in 2012, the first year for which Medicare spending data were available.”*

• Line 25: Typographical correction: “conversial” → “conversion”.

Authors: This change has been made.

• Lines 24–26: Replace “i.e.” with “meaning” for more natural flow.

Authors: This change has been made.

• Lines 28–29: Specify which aspects were reported according to CHEERS (e.g., model structure, costing methods).

Authors: We have clarified that CHEERS is not directly applicable to our study. We now note in the Methods section (**page 6, line 26**):

> *“As our analysis compares accelerated versus regular approval of cancer drug indications, rather than evaluating a specific health intervention, no single reporting guideline is directly applicable. However, our reporting aligns with the relevant items in the Consolidated Health Economic Evaluation Reporting Standards 2022 (CHEERS 2022) Statement.35”*

• Line 46: Minor wording edit: “We sought to estimate…” → “We estimated…”

Authors: This change has been made.

• Lines 7–11: Smooth out mechanical flow caused by multiple “We…” sentences; introduce linking phrases.

Authors: We have revised this paragraph as follows (**page 7, line 13**):

> *“We used OptumInsights Clinformatics to extract de-identified medical and pharmacy claims for each drug annually from 1 January 2012 to 31 December 2020. To link each patient to a specific indication, we identified the proportion of new patients taking the drug for a given indication each year. This involved first distinguishing patients who were new to the therapy from those who had used the drug in prior years. For the subpopulation of new users, we then calculated indication-specific shares using the first claim for each therapy.”*

• Lines 19–20: Reword a complex sentence about attribution of indications to improve readability and flow.

Authors: We have revised this sentence as follows (**page 7, line 24**):

> *"In cases where a single dispensing could be linked to multiple indications, we attributed utilisation to the indication associated with an overall survival benefit. If no indication had an overall survival benefit, utilisation was attributed to the indication with the earliest approval date."*

• Lines 38–42: Clarify whether identification of confirmatory trial-linked publications was systematic or manual.

Authors: We have clarified that identification of confirmatory trial-linked publications was systematic (**page 8, line 3**).

> *"We then systematically reviewed approval letters in the Drugs@FDA database to identify post-marketing confirmatory trials requested at the time of accelerated approval and their projected completion dates."*

• Lines 56–57: Minor rewording: "…categorised each indication based on whether a statistically significant overall survival benefit was demonstrated…"

Authors: This change has been made.

• Line 19: Correct grammar: "spending…was available" (not "were").

Authors: This change has been made.

• Lines 29–31: Suggest softening blunt phrasing from "alive and dead" to "alive and deceased".

Authors: We can confirm that "alive" and "dead" are standard terms used in partitioned survival modelling to define health states.

• Lines 35–36 and 45–46: Recommend explicitly acknowledging the simplification inherent in the exponential curve fitting method.

Authors: We have fully acknowledged this point in the revised manuscript. We now state (**page 9, line 8**):

> *"We estimated the proportion of patients alive over time by fitting an exponential curve to the median survival data, a pragmatic approach for long-term extrapolation in the absence of individual participant-level data. The exponential distribution is widely used in economic evaluations of cancer treatments. We assumed proportional hazards throughout the model to reflect a constant treatment effect on survival."*

• Line 37: Suggest rephrasing to clarify that including non-significant survival results was a deliberate modelling decision.

Authors: We have made this change (**page 9, line 14**).

> *"To maximise use of available data, the model incorporated the most recent overall survival data, irrespective of the statistical significance of the findings."*

• Lines 46–47: Recommend briefly explaining why the model did not discount future life-years and costs, as discounting is standard in cost-effectiveness analyses.

Authors: We have now explained why the model did not discount life-years and costs. Specifically, we now write (**page 9, line 23**):

> *"Given the retrospective nature of the analysis (estimating life-year gains and costs through December 2022), the model did not discount life-year gains or additional spending."*

• Lines 50–51: Minor stylistic improvement: "we ran 10,000 Monte Carlo simulation iterations for each drug indication to capture uncertainty."

Authors: We have revised this sentence to read (**page 9, line 33**):

> *"To estimate uncertainty, we conducted probabilistic sensitivity analyses using Monte-Carlo simulations with 10,000 iterations per drug indication."*

Results

• Line 7: Typographical clarification: reword to "excluding 7 indications, corresponding to 5 drugs, for which Medicare spending data were unavailable."

Authors: This change has been made (**page 11, line 5**).

• Lines 19–22: Reword for precision: "while the remaining 26 indications were converted to regular approval based on surrogate endpoints, despite lacking evidence of overall survival benefit or having unknown benefit."

Authors: We have revised this sentence to read as follows (**page 11, line 18**):

> *"the remaining 26 were converted to regular approval based on surrogate endpoints: 15 with no evidence of overall survival benefit and 11 with unknown overall survival benefit (6 due to single-arm trial designs and 5 due to immature survival data)."*

• Lines 25–26: Simplify phrasing: "Randomised controlled trials found no evidence of overall survival benefit for 30 indications (33.3%), including 11 that were later converted to regular approval."

Authors: This change has been made.

• Lines 31–32: Strengthen framing: "178,708 beneficiaries received drugs through early access via the FDA's accelerated approval pathway, often before benefits were confirmed."

Authors: We have revised this sentence to read (**page 11, line 28**):

> *"An estimated 178,708 Medicare beneficiaries received early access to new cancer drugs following the FDA's accelerated approval."*

• Line 39: Typographical correction: "18.0%%" → "18.0%."

Authors: We have made this correction.

• Lines 36–39: Reword for clarity: "142,760 beneficiaries for indications eventually converted to regular approval, 5,574 for indications still under accelerated approval, and 30,374 for indications later withdrawn."

Authors: This change has been made.

• Lines 46–48: Recommend directly quantifying the difference in beneficiaries receiving drugs with vs. without survival benefit (e.g., "about 17,000 vs. 9,000 in 2020") to make the comparison more tangible.

Authors: We have now added this information. The revised sentence reads (**page 11, line 42**):

> *"In 2020, 17,455 beneficiaries received drugs for indications that had no overall survival benefit, which was nearly double the 9.517 beneficiaries who received drugs for indications with demonstrated overall survival benefit."*

• Line 53: Typographical correction: "life-years gained" (insert hyphen).

Authors: This change has been made.

• Lines 7–8: Clarify phrasing: "resulted in no observed life-year gains in survival outcomes."

Authors: As "life-year gains" already encapsulate survival outcomes, we have not made this change.

• Line 14: Grammar correction: "costs…were $20.1 billion."

Authors: We have made this correction.

• Line 23: Recommend clarifying that many indications converted to regular approval did so without strong survival evidence.

Authors: As these results area already summarised in paragraph 3 of the Results section, we suggest maintaining the current structure to avoid unnecessary repetition.

Discussion:
• Line 5: Wording improvement for impact: "We quantified the life-year gains and costs of use of new cancer drugs for indications granted FDA accelerated approval" → "We evaluated how early access to new cancer drugs through the FDA's accelerated approval pathway translated into real survival gains and additional costs for Medicare beneficiaries."

Authors: We thank the Reviewer for this suggestion. While the original sentence removed, the updated paragraph in the Discussion closely reflects the intent of the Reviewer's proposed revision. Specifically, we now state (**page 13, line 4**):

> *"This early access was associated with an estimated 76,164 additional life-years. However, these gains came at a substantial cost: approximately $20.1 billion in additional Medicare spending."*

• Line 9: Minor phrasing edit: "…at an additional cost to the Medicare program…" → "…at an added cost of approximately $20.1 billion to Medicare."

Authors: This sentence is no longer included in the revised Discussion section.

• Lines 8–10: Reword for better nuance: "…treatments to reach patients sooner, though often with greater uncertainty than under traditional approval processes."

Authors: This sentence is no longer included in the revised Discussion section.

• Lines 17–19: Improve phrasing for clarity: "…many conversions to regular approval occurred without evidence of improved survival."

Authors: We have revised this sentence as follows (**page 13, line 12**):

> *"Although the majority of use and spending occurred in indications that were ultimately verified, the FDA often converted accelerated approvals to regular approvals without evidence of overall survival benefit, consistent with findings from prior studies."*

• Lines 17–19: Minor edit for sharper phrasing: "…would better align Medicare spending with treatments that offer proven clinical benefit."

Authors: This change has been made.

• Lines 31–36: Suggest briefly referencing an example of value-based pricing or outcomes-based reimbursement initiatives to strengthen proposals.

Authors: We have added a supporting reference, as suggested by the Reviewer (**page 15, line 3**).

• Line 42: Improve clarity: "…which can lead to continued patient exposure to ineffective treatments and undermine trust in clinical recommendations."

Authors: This change has been made (**page 15, line 10**).

• Lines 44–53: The international comparison feels slightly rushed; recommend adding a summarising

sentence to emphasise that without harmonised standards, uncertainty about benefit may persist globally.

Authors: We have added the following transition sentence (**page 15, line 13**):

> *"These challenges are not confined to the U.S. healthcare system, as regulatory decisions made by the FDA often influence drug availability and clinical practice in other countries."*

• Lines 4–5: Wording improvement to avoid self-promotion: rephrase "makes an important and novel contribution" to "provides a new, integrated analysis of survival benefits, spending, and approval outcomes for cancer drugs under the accelerated approval pathway."

• Lines 9–10: Softer phrasing to avoid overstating: "extends previous analyses to provide a broader evaluation of…" instead of "comprehensively evaluate…"

Authors: We appreciate the Reviewer's suggestions regarding tone and phrasing. We have carefully considered these points. Our current wording was chosen to reflect the scope and integration of multiple dimensions (survival outcomes, spending, and regulatory outcomes) within a single analysis. We believe this framing accurately conveys the contribution of our study without overstating its impact. As such, we have retained the original phrasing.

Conclusion:
• Lines 50–51: Wording improvement: "…with just three drug indications responsible for over two-thirds of the total life-year gains" for smoother and more active phrasing.

Authors: This change has been made.

Overall, the authors have produced a clear and timely analysis of an important regulatory and policy issue. This paper is a strong contribution and merits acceptance following minor revision.

Authors: We thank the Reviewer for this positive assessment of our paper.

Reviewer: 2

Recommendation:

Comments:
In this study, Naci and colleagues compared the gains in overall survival against the incremental costs of new cancer drugs that received early access due to an accelerated approval by the FDA. This was specifically investigated in the U.S. Medicare setting, where accelerated approvals mark a growing share of the drug expenses. This study raises the question of whether it is sufficient to assess clinical effectiveness based on surrogate parameters. While the study topic is certainly highly relevant and the inclusion of all 90 cancer drugs in accelerated approval makes it comprehensive, the methodological approach often seems simplistic and its reporting is inadequate.

Authors: We thank the Reviewers for their detailed assessment of our manuscript. While our study applies principles of economic evaluation, it is not a conventional cost-effectiveness analysis of a single health intervention. Rather, it is a policy evaluation that examines the trade-offs associated with the FDA's accelerated approval pathway across 90 cancer drug indications. As such, reporting standards developed for economic evaluations of individual interventions are not directly applicable to our study design or objectives.

Our analysis necessarily involves simplifying assumptions to enable a comprehensive evaluation across all 90 cancer drug indications granted accelerated approval by the FDA between 2012 and 2020. We appreciate the Reviewers' comments, which have helped us improve the clarity with which we communicate these assumptions in the revised manuscript.

Importantly, our approach prioritises clinical and policy relevance over detailed modelling of individual treatments, which would not be feasible given the scope of our analysis. This design allows us to assess systemic patterns in survival gains and Medicare spending when new cancer drugs receive accelerated approval, compared to a counterfactual scenario in which approval occurs at the time of confirmatory trial completion.

We believe this approach represents an important advance over prior studies on accelerated approvals. By quantifying the broader trade-offs in survival and spending, our study provides a more comprehensive evaluation of the accelerated approval pathway and its implications for patients and the healthcare system.

The following comments should be considered to improve the main messages of the manuscript:

Major comments:
1) Overall lack of clarity and reporting regarding the rationale for the choice of methods.
a) Synthesis of the data from the respective RCTs (several trial identifiers) for the 90 drugs and the extrapolation thereof was not described; it seems that 90 meta-analyses may have been conducted, but were not reported in the manuscript.

Authors: The trial identifiers listed in appendix table 2 refer to the confirmatory trials required by the FDA at the time of accelerated approval. In some cases, the FDA requested more than one trial to address outstanding clinical uncertainties. However, for each cancer drug indication included in our analysis, there was a maximum of one RCT that reported overall survival data. As such, we did not conduct any meta-analyses. Instead, we extracted overall survival data directly from the relevant RCT (or single-arm trial) for each indication. These data, along with their precise sources, are detailed in appendix table 3. We have now more prominently referenced this table in the Results section of the revised manuscript (**page 11, line 17**).

b) In line with the BMJ open code policy, the statistical code for the analysis should be provided to aid interpretation and reproducibility.

Authors: We appreciate the Reviewers' emphasis on transparency and reproducibility. All data extracted from publicly available sources, including accelerated approval status, confirmatory trial characteristics, and overall survival findings, are included in the supplementary appendix, as noted in our Data sharing statement (**page 18, line 2**):

> *"All data extracted from publicly available sources (accelerated approval status and confirmatory trial characteristics of drug indications in our sample, overall survival findings) are available in the supplementary appendix. Access to data from the Medicare Advantage plans can be directly requested from the data vendor, and requires a data use agreement."*

In addition, we would be happy to make our economic model available via our institutional website. We have added this to the Data sharing statement (**page 18, line 5**):

> "The economic model will be made available via our institutional website upon publication."

c) The research question is not clearly stated (p. 2, l. 5-9; p. 5, l. 16-18) and the main conclusion remains vague throughout the manuscript (p. 2, l. 42-44; p.3, l. 15-16; p. 14, l. 43).

Authors: We have revised the objective section of the abstract to clarify the research question. The updated objective now reads (**page 2, line 3**):

> *"To evaluate the survival gains and additional Medicare spending associated with earlier access to new cancer drugs granted accelerated approval by the U.S. Food and Drug Administration (FDA), compared with the regular approval process."*

We have revised the concluding sentence of the Introduction section (**page 5, line 14**):

"In this study, we evaluated the expected survival benefits and additional Medicare spending associated with earlier access to new cancer drug indications granted accelerated approval by the FDA, compared with access following regular approval."

We have also revised the main conclusion throughout the manuscript (**page 2, line 36**).

"Accelerated approval yielded uneven survival returns, with a small number of drugs accounting for the majority of life-year gains. Medicare incurred substantial costs for treatments that, in over half of cases, did not provide an overall survival benefit."

d) The comparison groups are not clearly explained, especially for the cost data from Medicare.

Authors: As noted in our response to the first Reviewer's question regarding comparison groups, we have added further detail and supporting data to clarify our approach.

In the Methods Overview section, we now state (**page 6, line 11**):

"We determined whether cancer drug indications initially granted accelerated approval had demonstrated overall survival benefit compared to control treatments in clinical trials."

To clarify our data extraction process, we added (**page 8, line 12**):

"We extracted information on treatment and control groups, study design, primary endpoint(s), and study results from pivotal and confirmatory trials."

We also revised the description of our spending calculations to emphasise the role of comparator treatments (**page 8, line 30**):

"We calculated as additional spending the difference in Medicare spending between the treatment strategy including the accelerated approval drug and the standard of care strategy without the accelerated approval drug, as tested in the control arms in clinical trials."

To address how we handled single-arm trials, we added (**page 8, line 35**):

"When only single-arm trials were available, we assumed no available treatments in our base-case analysis."

We further clarified this in the scenario analysis (**page 9, line 50**):

> *"In the base-case analysis, we assumed no relevant comparator treatment when a drug was evaluated in a single-arm trial. For a scenario analysis (spending scenario 1), we identified potentially relevant comparators by searching ClinicalTrials.gov using the drug name and its accelerated approval indication, and by reviewing FDA medical review documents available on Drugs@FDA for references to existing treatment alternatives."*

Finally, we added a supplementary table listing comparator treatments, indicating which were deemed relevant for drugs evaluated in single-arm trials.

e) The authors should add more explanation to the Medicare system & Medicare Advantage in the introduction section, as this may not be entirely clear to an international audience.

Authors: We have expanded the description of the Medicare program in the Introduction and added a supporting reference (**page 5, line 4**):

> *"This question is particularly important for the federal Medicare program, which provides health insurance for Americans 65 years and older. Medicare consists of two main components: traditional Medicare, which is government-administered, and Medicare Advantage, which is offered by private insurers.[31]"*

f) Sufficient reasoning for the executed sensitivity analysis are not provided and the results in Fig. 7 are not informative for the underlying research question. Other sensitivity analyses are recommended (see below, e.g., discount rates, trial type, drug prices, effect size).

Authors: As recommended by the first Reviewer, we have added a paragraph in the Methods section to introduce and justify our scenario analyses more clearly. These analyses were designed to test the robustness of our findings under alternative assumptions. This paragraph reads (**page 9, line 39**):

> *"To assess the robustness of our findings to data limitations, we conducted several scenario analyses. These included: (1) varying comparator treatments for indications supported by single-arm trials or trials with multiple comparators, which affected estimated incremental spending; (2) reallocating drug utilisation across similar indications to account for limitations in claims data, which influenced both spending and survival outcomes; and (3) modeling survival benefits in the absence of overall survival data, which affected estimated life-years gained. We also varied the definitions of the duration of early access, which we hypothesised would have the greatest impact on results, as this assumption applied to all indications in our sample."*

We address the Reviewer's suggestions regarding additional sensitivity analyses in response to their specific comments below. We believe our current set of analyses captures the key uncertainties relevant to our policy-focused evaluation.

2) Some essential components of the cost-effectiveness analysis are missing, which very likely underestimates costs and overestimates effectiveness, therefore overestimating overall the true cost-effectiveness.

Authors: We appreciate the Reviewers' comment and would like to reiterate that our study is not a conventional cost-effectiveness analysis of a single health intervention. Rather, our objective is to evaluate the FDA's accelerated approval pathway a policy mechanism to enable earlier access to new cancer drugs compared to access in traditional approval. To do so, we compared 'early' vs 'regular' approval across 90 cancer drug indications granted accelerated approval during the study period. Given this policy-level focus, our approach necessarily deviates from traditional cost-effectiveness analyses, which typically assess an individual intervention against its comparators.

a) Only life-years gained, but no QALYs were considered. Even if such data is not available, the authors could provide a range of common utilities for novel cancer drugs based on the literature. Depending on side-effects, cost per QALY may be 20-70% higher than the cost per LYG reported. The authors also don't report a reference for the 150.000$ per LYG gained as cost-effectiveness threshold and I suspect this was set for QALYs and not LYG.

Authors: We fully acknowledge that our analysis does not incorporate QALYs, as such data are not available for the vast majority of products included in our study. In fact, only 5% of cancer drug indications approved by the FDA during our study period had publicly available data on quality of life, which would be even fewer for drug indications granted accelerated approvals. We therefore refrained from making assumptions about the quality-of-life benefits of these treatments, which would have introduced additional uncertainty into our estimates. We have added this to the Limitations section of our revised paper (**page 16, line 18**):

> *"Fourth, our assessment of benefit was limited to overall survival, and we did not consider potential quality-of-life improvements for drug indications without a survival benefit. Previous research indicates that quality-of-life improvements are rare.[72] Fewer than 5 percent of cancer drugs approved by the FDA between 2000 and 2020 demonstrated quality-of-life benefits.[73]"*

Regarding the cost-effectiveness threshold, the $150,000 benchmark is typically applied to cost per QALY. However, we note that the Institute for Clinical and Economic Review (ICER) also applies this threshold to "equal-value life-years gained (evLYG), which are conceptually similar to unadjusted life-years gained when quality-of-life data are unavailable. We have now added a reference to ICER's value assessment framework to clarify this point in the manuscript (**page 9, line 27**).

> *"We compared the estimated spending per life-year gained against commonly used U.S. cost-effectiveness thresholds, which range from $100,000 to $150,000 per quality-adjusted life-year (QALY) or equal-value life-year gained, as recommended by the Institute for Clinical and Economic Review.[53,54] Since no formal threshold exists for life-years gained, we use this range as a proxy for what a decision maker might be willing to pay for survival benefits."*

b) The fit of the exponential function within the two-stage model seems overly simplistic. Some trials only provide data for 1-2 years, but it seems like survival is extrapolated up until 10 years. I would suggest a sensitivity analysis limiting survival estimation to the actual trial durations. The exponential function can also not accommodate any plateaus or other dynamics in the tail of the distribution. I would suggest the authors report sensitivity analyses with other, more flexible parametric alternatives.

Authors: We thank the Reviewers for this comment. Given the scope of our study, which includes 90 cancer drug indications, we required a consistent and parsimonious method for extrapolating survival in the absence of individual participant level data or Kaplan-Meier curves.

We have clarified our modelling approach in the revised manuscript and provided additional justification for our use of the exponential distribution to extrapolate survival beyond the trial duration.

In the Methods section, we now write (**page 9, line 7**):

> *"We tracked life-year gains with the accelerated approval drug and control treatment based on overall survival data extracted from clinical trials. We estimated the proportion of patients alive over time by fitting an exponential curve to the median survival data, a pragmatic approach for long-term extrapolation in the absence of individual participant-level data. The exponential distribution is widely used in economic evaluations of cancer treatments. We assumed proportional hazards throughout the model to reflect a constatnt treatment effect on survival."*

We have also explicitly acknowledged the limitations of this approach (**page 16, line 5**):

> *"Second, our extrapolation of survival data relied on the exponential distribution. While this approach is standard in economic evaluations of treatments for advanced cancers, alternative parametric distributions may provide a better fit to the underlying data. However, applying such alternatives would require individual participant-level data and clinical validation, which were not available. In many cases, even Kaplan-Meier curves were not available, for example when the most recent overall survival data were extracted from clinicaltrials.gov. Additionally, the model adopted a limited time horizon, which may have led to underestimation of total life-years gained."*

c) No discounting was applied and no reasoning for this decision has been provided. This will again underestimate the true costs compared to a standard cost-effectiveness approach.

Authors: Unlike traditional cost-effectiveness analyses that project future outcomes over extended time horizons (where discounting of costs and benefits is standard practice) our analysis is retrospective. As such, we intentionally did not apply discounting.

To ensure comparability over time, we adjusted all spending figures for inflation and report results in constant 2022 US dollars. We have clarified this rationale in the revised Methods section. We now clarify this approach in our revised Methods section (**page 9, line 23**):

> *"Given the retrospective nature of the analysis (estimating life-year gains and costs through December 2022), the model did not discount life-year gains or additional spending."*

d) Information as requested by the CHEERS Checklist is not fully provided (e.g. No. 1, No. 5, No. 10).

Authors: As noted earlier, our analysis is not a conventional cost-effectiveness analysis of a single health intervention. Therefore, the CHEERS 2022 checklist is only partially applicable to our study design and objectives.

We have clarified this in the Methods section of the revised manuscript with the following statement (**page 6, line 26**):

> *"As our analysis compares accelerated versus regular approval of cancer drug indications, rather than evaluating a specific health intervention, no single reporting guideline is directly applicable. However, our reporting aligns with the relevant items in the Consolidated Health Economic Evaluation Reporting Standards 2022 (CHEERS 2022) Statement.[35]"*

3) The algorithm extrapolating from the selective Medicare Advantage population to the broader Medicare population is not explained at all and the provided references do not explain it in detail either. While the authors claim that no differences in cancer prevalence were found between Medicare Advantage vs. traditional Medicare this does not automatically translate to no differences in prescription and filling/billing of treatments investigated here. Contrary to the authors claims there are also systematic differences between these groups (e.g., in geographic distribution, proportion of non-white individuals, education, income, etc.) which indicates a clear selection bias (also see https://www.kff.org/report-section/disparities-in-health-measures-by-race-and-ethnicity-among-beneficiaries-in-medicare-advantage-report/).

Authors: We have elaborated further on these points in our revised paper. First, we have added the following example to illustrate our approach more concretely for readers who are not familiar with this context and may appreciate more details (**page 7, line 37**):

“For example, in 2013, the first year with CMS data available for brentuximab vedotin, we estimated that 85% of patients were new users, and 50% of those new users received the drug for an accelerated approval indication. CMS reported that 645 Medicare beneficiaries received the drug in 2013. Based on our estimates, 548 of these were new users (85% of 645), and 274 of those were treated for the accelerated approval indication (50% of 548). Because this is a Part B drug and CMS data reflect only traditional Medicare, we adjusted for the fact that traditional Medicare accounted for 72% of total Medicare enrollment in 2013. The adjusted denominator was therefore 896, and the estimated number of new patients using the drug for the accelerated approval indication was 381.”

Secondly, we have added more detail about the differences between Medicare Advantage and traditional Medicare (**page 7, line 7**):

“We assumed that utilisation patterns of accelerated approval drugs are broadly similar between beneficiaries in Medicare Advantage and traditional Medicare. Although these populations differ in important ways, including geographic distribution, race and ethnicity, income and education, earlier research has found no systematic differences in cancer prevalence or injectable drug spending between them.[38]”

Third, we have expanded our limitations section (**page 15, line 42**):

“First, we estimated the share of patients receiving therapy with cancer drugs in their accelerated approval indications using data from Medicare Advantage plans administered by a large insurer. While previous research has found no systematic differences in cancer prevalence or treatment dynamics between Medicare Advantage and traditional Medicare populations, including injectable drug spending,[38,70] important differences remain that may influence treatment patterns. For example, a recent study suggested that utilisation of high-cost cancer drugs may be lower in Medicare Advantage for indications with low-cost treatment alternatives.[71] Although this is unlikely to substantially affect our estimates (accelerated approval indications involve novel therapies for which low-cost alternatives are often unavailable), the findings may not be fully generalisable to the broader Medicare population. Additionally, reliance on data from a single insurer may further limit generalisability. Nonetheless, our approach is consistent with prior work that use the same data source.”

a) It would help if the authors provide some baseline characteristics of the study population, e.g. a descriptive analysis of the Medicare Advantage population.

Authors: We appreciate the Reviewers’ suggestion. However, providing baseline characteristics of the Medicare Advantage population is outside the scope of our analysis. Moreover, descriptive data on the Medicare Advantage population are widely available in existing literature and public reports.

b) The Medicare Advantage population of a single commercial health insurer is likely not

generalizable to the entire Medicare population. This should be discussed in the Limitation section.

Authors: We have now added this point to the limitations section of the manuscript (**page 16, line 2**):

> *"Additionally, reliance on data from a single insurer may further limit generalisability. Nonetheless, our approach is consistent with prior work that use the same data source."*

Minor comments:

Abstract:

4) General: The abstract does not clarify the methods and analyses done to evaluate the cost-effectiveness analysis (e.g., interventions are not clearly stated).

Authors: Our revised objective now clarifies that the comparison is between accelerated versus regular approval for each cancer drug indication that received accelerated approval. To further clarify our approach, we have now added the following sentence to the abstract (**page 2, line 3**):

> *"**Objective:** To evaluate the survival gains and additional Medicare spending associated with early access to new cancer drugs granted accelerated approval by the U.S. Food and Drug Administration (FDA), compared with access following completion of confirmatory trials."*

5) Participants: (1) Please add more information about the study population (+65 years) as this may not be clear to readers outside the US. (2) p. 2, l. 15: I recommend writing "through to" instead of "through" for clarity.

Authors: We have now incorporated this into the revised Abstract (**page 2, line 7**):

> *"**Setting:** U.S. Medicare program, which provides health insurance for adults aged 65 years and older."*

We have also used "through to", as recommended by the Reviewers.

What this study adds:

6) P. 3, l. 20: How do the authors conclude that the additional spending was "substantial"? Please include the WTP threshold that was used to interpret the findings and explain whether the threshold is commonly used in the context of overall survival or rather applied to other endpoints (like QoL).

Authors: We now explain that our interpretation of "substantial" spending is based on commonly used U.S. cost-effectiveness thresholds. Specifically, we refer to the threshold range of $100,000-$150,000 per QALY, as used by the Institute for Clinical and Economic Review (ICER). Importantly, ICER also applies this threshold to equal-value life-years gained" (evLYGs), which are conceptually similar to unadjusted life-years gained.

Our revised Methods section now includes the following (**page 9, line 27**):

> *"We compared the estimated spending per life-year gained against commonly used U.S. cost-effectiveness thresholds, which range from $100,000 to $150,000 per quality-adjusted life-year (QALY) or equal-value life-year gained, as recommended by the Institute for Clinical and Economic Review.[53,54] Since no formal threshold exists for life-years gained, we use this range as a proxy for what a decision maker might be willing to pay for survival benefits."*

Introduction:

7) P. 4, l. 47: This is the same sentence already mentioned in the "participants section" of the abstract and is not clearly phrased.

Authors: We thank the Reviewers for this comment. We have now revised this sentence, which reads (**page 4, line 43**):

> *"Earlier access has benefited some patients for whom the survival gains associated with treatment were substantial, such as those treated with imatinib which transformed outcomes for patients with chronic myeloid leukemia.[27]"*

8) P. 4, l. 46: This sentence is not clearly phrased. Maybe it should read "some cancer drugs" or "most cancer drugs"?

Authors: We have revised this sentence to improve clarity. It now reads (**page 4, line 41**):

> *"According to the FDA, this pathway has enabled cancer drugs to enter the market sooner than they otherwise would."*

9) P. 5, l. 13: The authors could include some proportions for the growing share of increase in cancer drugs.

Authors: We appreciate the Reviewers' suggestion. The paragraph already includes several data points characterising trends in cancer drug spending in Medicare. We believe the current level of detail sufficiently supports the context and focus of our analysis, and have therefore not added further proportions.

Methods:

10) Please provide information on the software used for the calculations and modeling.

Authors: We have clarified the software used in the revised Methods section. All statistical analyses were conducted using Stata, and the partitioned survival modelling was implemented in Microsoft Excel.

The revised Methods section includes the following (**page 7, line 47**):

> *"This stepwise approach was implemented using Stata (version 18) and allowed us to estimate the total number of patients receiving new cancer drugs for indications that initially received FDA accelerated approval in all of Medicare each year."*

In addition, we added (**page 6, line 14**):

> *"To estimate life-year gains and additional spending for new cancer drug indications granted FDA accelerated approval, we developed a partitioned survival model in Microsoft Excel with two states: alive and dead (**appendix figure 2**).[43]"*

11) How do drugs/indications based on single-arm trials enter the results? How are the life-years gained estimated in the absence of a control group?

Authors: We appreciate the opportunity to clarify our methodological assumptions regarding single-arm trials. In such trials, where no concurrent control group is available, we assumed that the comparator arm would have the same overall survival as the intervention arm.

In the Methods section, we stated (**page 9, line 49**):

> *"In the base-case analysis, we assumed no relevant comparator treatment when a drug was evaluated in a single-arm trial."*

To further clarify, we also noted (**page 10, line 17**):

> *"In our base-case analysis, we assumed patients receiving drugs for indications with no overall survival data (e.g., due to single-arm trial designs) derived no life-year gains from earlier access."*

12) How was the availability of OS benefit classified, e.g. Appendix Table 3: for drug ID 1, the OS benefit was classified as "no", but the HR CI does not contain 1 and indicates a protective effect of the intervention.

Authors: Our classification of overall survival benefit was based on established conventions for determining statistical significance, which consider both the 95% CI and the p-value of the hazard ratio, as well as any pre-specified thresholds for significance. In the case of drug ID 1, although the HR CI did not cross 1, the result did not meet the pre-specified p-value threshold for statistical significance. In addition, the FDA concluded that the indication was not associated with a survival benefit, which led to the decision to withdraw the drug indication from the market.

13) P. 6, l. 19: The people receiving control treatments are not listed as participants, but their data seems to have been used, please explain.

Authors: To clarify, the comparison in our analysis was between early and regular approval of each cancer drug indication, rather than between intervention and control arms within clinical trials. The extracted data on the difference between intervention and control arms from clinical trials were used to estimate the magnitude of survival benefit that could be expected for patients receiving each drug indication. These trial-derived estimates informed our modelling of the potential impact of earlier versus later access to accelerated approval drugs.

14) P. 6, l. 23-24: Given the different follow-up periods starting in 2012 and ending in 2020, 2022 and 2024, respectively, the authors should consider creating a timeline figure that shows the study design to help interpretation.

Authors: We thank the Reviewers for this suggestion. We have now created a timeline figure, which is available in the supplementary materials.

15) P. 6, l. 29: Please add a reference to the appendix for the CHEERS Checklist.

Authors: We can confirm that the appendix already includes a reference for the CHEERS Checklist.

Results:

16) Please mention the results of the probabilistic sensitivity analysis (apart from Fig. 7).

Authors: We can confirm that the 95% confidence intervals reported throughout the Results section are obtained from the probabilistic sensitivity analysis. This analysis is relevant for the results of the modelling used to estimate life-year gains and additional Medicare spending.

17) Figure 7: Please describe in the Figure or caption how the base case and the scenarios are defined.

Authors: We thank the Reviewers for this suggestion. The definitions of the base case and scenario analyses are currently described in the Methods section. If the editors agree, we would be happy to also include this information as a footnote to Figure 7.

18) Figure 4: The Figure is well presented, but would benefit from CIs. Please also consider adding CIs to the Figures 1, 2 & 7.

Authors: Figures 1, 2, 4, and 7 present results from deterministic analyses, which do not incorporate parameter uncertainty and therefore do not include CIs. As such, we report 95% CIs only for results derived from the probabilistic sensitivity analysis, where uncertainty is explicitly modelled. This is reflected in the figures and results where applicable.

19) Please check whether some percentages have been calculated correctly, e.g., p. 10. l. 39/l.54, Table 1 (5.6% instead of 5.5% for multiple myeloma, 17.8% instead of 17.7% for accelerated approval status: withdrawn).

Authors: We can confirm that the percentages are correct and reflect rounding to ensure that category totals sum to 100%.

Discussion:

20) P. 12, l. 12: Please mention the cost-effectiveness threshold and provide a reference.

Authors: We have now included the cost-effectiveness threshold in the manuscript (**page 9, line 27**).

> *"We compared the estimated spending per life-year gained against commonly used U.S. cost-effectiveness thresholds, which range from $100,000 to $150,000 per quality-adjusted life-year (QALY) or equal-value life-year gained, as recommended by the Institute for Clinical and Economic Review.[53,54] Since no formal threshold exists for life-years gained, we use this range as a proxy for what a decision maker might be willing to pay for survival benefits."*

21) P. 12, l. 15: The authors should discuss the implications for pricing after drugs receive regular approval.

Authors: We have now elaborated further on the association between drug benefit and price in the accelerated approval pathway.

In the revised Discussion section, we now write (**page 14, line 48**):

> *"First, drug prices should reflect the magnitude of clinical uncertainty.[30] Currently, there is no consistent association between drug prices and demonstrated clinical benefit.[58] Even when confirmatory trials report improvements in overall survival, prices for cancer drugs granted accelerated approval do not increase accordingly, limiting financial incentives for companies to generate evidence on overall survival.[59] CMS could implement reimbursement policies that*

lower payment rates until drugs demonstrate clinical benefit, thereby incentivising manufacturers to conduct rigorous confirmatory trials.[60]"

22) P. 12, l. 46-49: The authors report that the duration between accelerated approval and conversion/withdrawal was longer for indications without survival benefit compared to those with survival benefit (36 months vs. 16 months). Please add this information to the results and methods section.

Authors: The comparison of time to conversion or withdrawal (36 months vs. 16 months) was conducted specifically to provide additional context for the breast cancer indications and was not systematically examined across all indications in our dataset. Given the already extensive data presented in the manuscript, we would prefer not to add further analyses to the Methods and Results sections.

Limitations:

23) The authors should discuss that accelerated approval drugs may show long-term benefits that are not fully captured at the chosen cut-off and that clinical trials provide limited data on that. That is why extrapolation is often used in models to account for this limitation.

Authors: We thank the Reviewers for this point. We have now acknowledged that our analysis may underestimate the benefit in some cases due to the follow-up period ending in 2022. Our revised limitations section now reads (**page 16, line 11**):

"Additionally, the model adopted a limited time horizon, which may have led to underestimation of total life-years gained."
